# A scalable unified framework of total and allele-specific counts for cis-QTL, fine-mapping, and prediction

**DOI:** 10.1038/s41467-021-21592-8

**Published:** 2021-03-03

**Authors:** Yanyu Liang, François Aguet, Alvaro N. Barbeira, Kristin Ardlie, Hae Kyung Im

**Affiliations:** 1 Section of Genetic Medicine, The University of Chicago, Chicago, IL USA; 2 The Broad Institute of MIT and Harvard, Cambridge, MA USA

**Keywords:** Gene expression, Gene regulation, Genomics

## Abstract

Genetic studies of the transcriptome help bridge the gap between genetic variation and phenotypes. To maximize the potential of such studies, efficient methods to identify expression quantitative trait loci (eQTLs) and perform fine-mapping and genetic prediction of gene expression traits are needed. Current methods that leverage both total read counts and allele-specific expression to identify eQTLs are generally computationally intractable for large transcriptomic studies. Here, we describe a unified framework that addresses these needs and is scalable to thousands of samples. Using simulations and data from GTEx, we demonstrate its calibration and performance. For example, mixQTL shows a power gain equivalent to a 29% increase in sample size for genes with sufficient allele-specific read coverage. To showcase the potential of mixQTL, we apply it to 49 GTEx tissues and find 20% additional eQTLs (FDR < 0.05, per tissue) that are significantly more enriched among trait associated variants and candidate cis-regulatory elements comparing to the standard approach.

## Abstract (web summary)

Methods for eQTL mapping from total and allele-specific gene expression typically are too computationally heavy to apply to large scale studies. Here, the authors describe a computationally efficient method to identify eQTLs, fine-map and predict expression from total and allele-specific gene expression

## Introduction

Genome-wide association studies (GWAS) have identified tens of thousands of genomic loci associated with complex traits, but most of these loci lie in noncoding regions of the genome, indicating transcriptome regulation as a potential key driver of disease biology. Multiple methods have been developed to integrate GWAS results with expression quantitative trait loci (eQTLs) and inform mechanisms underlying GWAS loci. Two strategies are commonly employed: (1) association-based approaches including PrediXcan^1^, fusion^2^, and smr^3^; and (2) colocalization-based approaches including coloc^4^, eCAVIAR^5^, and enloc^6^. Association-based approaches correlate genetic predictors of gene expression with complex traits of interest. Colocalization-based approaches rely on high-quality eQTL mapping and fine-mapping results to identify potentially causal genes.

In addition to gene expression levels measured by total read counts, allele-specific expression (the relative expression difference between the two haplotypes) provides valuable additional information that can be leveraged to improve eQTL mapping and fine-mapping. Several methods have been proposed to combine total and allele-specific read counts for QTL mapping, such as TReCASE^7^, WASP^8^, and RASQUAL^9^). However, running these methods on sample sizes beyond a few hundred is generally computationally intractable, and as a result they have not been applied to large-scale studies like GTEx, which includes over 15,000 samples across 49 tissues. For fine-mapping, two approaches that combine both allele-specific expression and eQTL mapping via meta-analysis have been recently proposed^10,11^. However, to our knowledge, no existing method provides a scalable unified framework combining total and allele-specific counts with explicit multi-SNP modeling for QTL mapping, fine-mapping, and prediction.

By assuming a log-linear model for transcript expression levels with independent reads from each haplotype and weak genetic effects, as proposed in ref. ^12^, we derive two approximately independent equations for allelic imbalance (read count ratio between the two haplotypes) and total read count. In this work, we develop a unified framework and computationally efficient algorithms combining total and allele-specific reads for QTL mapping, fine-mapping, and prediction. We demonstrate the resulting gain in performance with simulations under a range of different settings, applications to GTEx v8 data^13^, and comparisons to a large-scale eQTL meta-analysis from eQTLGen^14^. We also generated mixQTL results for the full set of GTEx data and make this resource publicly available. The software, simulation, data preprocessing, and analysis pipelines can be found at https://github.com/hakyimlab/mixqtl^15^, https://github.com/liangyy/mixqtl-pipeline^16^, and https://github.com/liangyy/mixqtl-gtex^17^. A computationally efficient GPU-based implementation of mixQTL has been embedded in tensorQTL https://github.com/broadinstitute/tensorqtl^18^.

## Results

### Overview of the statistical model

To develop a computationally efficient approach that integrates total and allele-specific count data, we assumed multiplicative cis-regulatory effects and noise, similarly to the model proposed in ref. ^12^. For a given gene, we modeled the haplotypic read count ${Y}_{i}^{h}$, which is the number of reads from haplotype *h* of individual *i* as

1 $${Y}_{i}^{h}={L}_{i}\cdot {\theta }_{0,i}\cdot \exp (\beta \cdot {X}_{i}^{h})\cdot \exp ({\epsilon }_{i}^{h}),$$

where *L*_*i*_ is the library size for individual *i*, *θ*_0,*i*_ is the baseline abundance (for a haplotype with the reference allele), $\exp (\beta )$ is the cis-regulatory effect (allelic fold change due to the presence of the alternative allele), ${X}_{i}^{h}$ indicates the dosage of the variant (0 if the individual has the reference allele, and 1 if they have the alternative one), and $\exp ({\epsilon }_{i}^{h})$ is the multiplicative noise.

Calculating the total read count as the sum of the two haplotypic counts and assuming weak cis-regulatory effects, we derived an approximately linear model for the logarithm of the haplotypic and total read counts (see details in Methods and Supplementary Notes 1). In practice, we only observe the allele-specific reads that include a heterozygous site denoted as ${Y}_{i}^{(h)\,\text{obs}\,}={\alpha }_{i}\cdot {Y}_{i}^{h}$, which is a fraction of the total haplotypic count. To take this partial readout into account, we modeled the observed total and allele-specific counts as

2 $${\mathrm{log}}\,{Y}_{i}^{(1)\,\text{obs}\,} 	= {\mathrm{log}}\,{L}_{i}+{\mathrm{log}}\,{\alpha }_{i}+{\mathrm{log}}\,{\theta }_{0,i}+{X}_{i}^{1}\beta +{\epsilon }_{i}^{(1)}\hfill\\ {\mathrm{log}}\,{Y}_{i}^{(2)\,\text{obs}\,} 	= {\mathrm{log}}\,{L}_{i}+{\mathrm{log}}\,{\alpha }_{i}+{\mathrm{log}}\,{\theta }_{0,i}+{X}_{i}^{2}\beta +{\epsilon }_{i}^{(2)}\hfill\\ {\mathrm{log}}\,\,\frac{{Y}_{i}^{\,\text{total}\,}}{2} 	\approx {\mathrm{log}}\,{L}_{i}+{\mathrm{log}}\,{\theta }_{0,i}+\frac{{X}_{i}^{1}+{X}_{i}^{2}}{2}\beta +{\epsilon }_{i}^{\,\text{trc}\,}$$

where the error terms are ${\epsilon }_{i}^{\,\text{trc}\,} \sim N(0,\frac{{\sigma }^{2}}{{Y}_{i}^{\,\text{total}\,}})$, ${\epsilon }_{i}^{(h)} \sim N(0,\frac{{\sigma }^{2}}{{Y}_{i}^{(h)\,\text{obs}\,}})$ and the errors of the two haplotypes are independent: *ϵ*^(1)^⊥⊥*ϵ*^(2)^. Here, we let the *ϵ* terms have variance inversely proportional to the actual count and by doing so, we ensure that the variance of the count scales approximately linearly to the mean of the count as demonstrated in Supplementary Notes 1.2.

We further simplified the models by combining the two allele-specific counts and defining the baseline abundance variation as a random effect *z*_*i*_ ($\mathrm{log}\,{\theta }_{0,i}$ = population mean + *z*_*i*_). Then, we merge the total count term ${\epsilon }_{i}^{\,\text{trc}\,}$ and *z*_*i*_ into one term ${\widetilde{z}}_{i}$ (since ${\epsilon }_{i}^{\,\text{asc}\,}$ is approximately independent from both of them. See Methods and Supplementary Notes 4.1). The final model is

3 $${\mathrm{log}}\,\frac{{Y}_{i}^{(1)\,{\text{obs}}\,}}{{Y}_{i}^{(2)\,{\text{obs}}\,}}\ =\ ({X}_{i}^{1}-{X}_{i}^{2})\beta \ +{\epsilon }_{i}^{\,\text{asc}\,}\ \,{({\rm{allelic}}\ {\rm{imbalance}}\ {\rm{eq.}})}\,$$

4 $${\mathrm{log}}\,\frac{{Y}_{i}^{\,\text{total}\,}}{2{L}_{i}}\ \approx {\mu }_{0}+\ \frac{{X}_{i}^{1}+{X}_{i}^{2}}{2}\beta \ +{\widetilde{z}}_{i}\ \,{({\rm{total}}\ {\rm{read}}\ {\rm{count}}\ {\rm{eq.}})}\,$$

where ${\widetilde{z}}_{i} \sim N(0,{\widetilde{\sigma }}_{0}^{2})$ and ${\epsilon }_{i}^{\,\text{asc}\,} \sim N(0,{\sigma }^{2}\cdot (\frac{1}{{Y}_{i}^{(1)\,\text{obs}\,}}+\frac{1}{{Y}_{i}^{(2)\,\text{obs}\,}}))$ and ${\widetilde{z}}_{i}$ is approximately independent from *ϵ*^asc^.

This single-SNP model extends to multiple SNPs in a straightforward manner by using a vector of allelic dosages (*X*_*i*1_, ⋯ , *X*_*ip*_) and genetic effects (*β*_1_, ⋯ , *β*_*p*_) instead of the scalar values above. Here, *p* represents the number of genetic variants in the cis-window of the gene under consideration (Supplementary Notes 3 and 5).

For cis-QTL mapping, we took advantage of the approximate independence of the allelic imbalance and the total read counts in Eqs. (3) and (4), solving them as separate linear regressions (for computational efficiency) and combining the results via inverse-variance weighted meta-analysis. We call this method mixQTL.

For the fine-mapping and prediction problems, we also leveraged the approximate independence of the allelic imbalance and total read count equations. We used a two-step approach in which we first scale the two equations so that they become independent data points with equal variances. In the second step, we combined these data points into an augmented dataset and applied the existing algorithms SuSiE^19^ and elastic net^20^. We term these methods mixFine and mixPred, for fine-mapping and prediction, respectively.

### Simulation of total and allele-specific reads

**Fig. 1:**
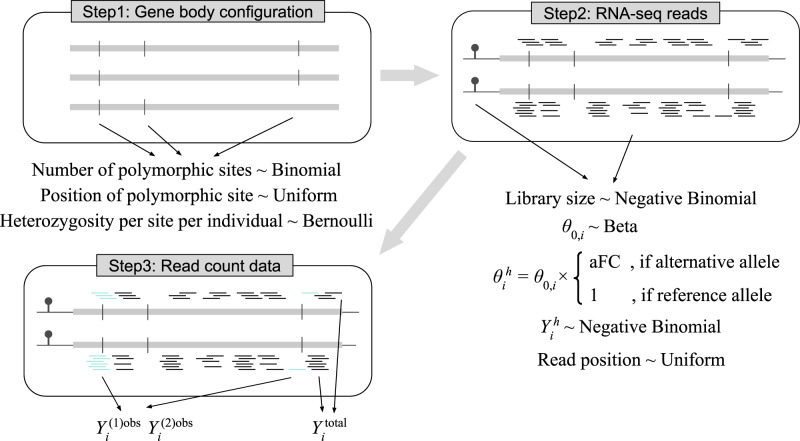
Simulation scheme for total and allele-specific read counts. Step 1 simulates a gene body configuration by first simulating the number of polymorphic sites of the gene followed by positioning these polymorphic sites uniformly across the gene body. For each individual, the heterozygosity of these polymorphic sites is drawn from a Bernoulli distribution. Step 2 simulates the haplotypic reads by first simulating Negative Binomial library size *L*_*i*_, Beta baseline abundance *θ*_0,*i*_, and the genetic effect *β*. These parameters determine the abundance ${\theta }_{i}^{h}$ for each haplotypic transcript, in which the allelic fold change, aFC, equals *e*^*β*^ in our parameterization. Then, the haplotypic read count ${Y}_{i}^{h}$ is generated using a Negative Binomial distribution given the expected count ${L}_{i}\times {\theta }_{i}^{h}$, where the reads are distributed uniformly across the gene body. In Step 3, the gene-level allele-specific counts ${Y}_{i}^{(h)\,\text{obs}\,}$ are determined by counting the reads that overlap heterozygous sites. ${Y}_{i}^{\,\text{total}\,}$ is calculated as the sum of the two haplotypic counts ${Y}_{i}^{1}$ and ${Y}_{i}^{2}$.

To assess the benefits of this unified framework over using only total read counts or allele-specific expression, we simulated haplotypic reads according to the framework illustrated in Fig. 1, with additional details in Methods and Supplementary Notes 6. For mixQTL, we simulated data with a single causal variant and for mixPred and mixFine, we simulated data with 1–3 causal variants.

For all simulation settings, we set an average library size of 94 million reads (to approximately match GTEx v8 library sizes) and used a series of expression levels (expected value of *θ*_0,*i*_ in Eq. (1)): from 50 to 1 read per million, corresponding to *θ* = 5 × 10^−5^–10^−6^. The fraction of allele-specific reads was kept at consistent levels across simulations by using the same distribution of polymorphic sites per individual.

### Combining total and allele-specific read counts improves cis-eQTL mapping

To assess the gain in power of combining total and allele-specific read counts, we simulated 200 replicates with allelic fold change varying among 1, 1.01, 1.05, 1.1, 1.25, 1.5, 2, 3. We compared mixQTL with two methods: using either only allele-specific counts (ascQTL) or total counts (trcQTL). See details in Supplementary Notes 4.1.

**Fig. 2:**
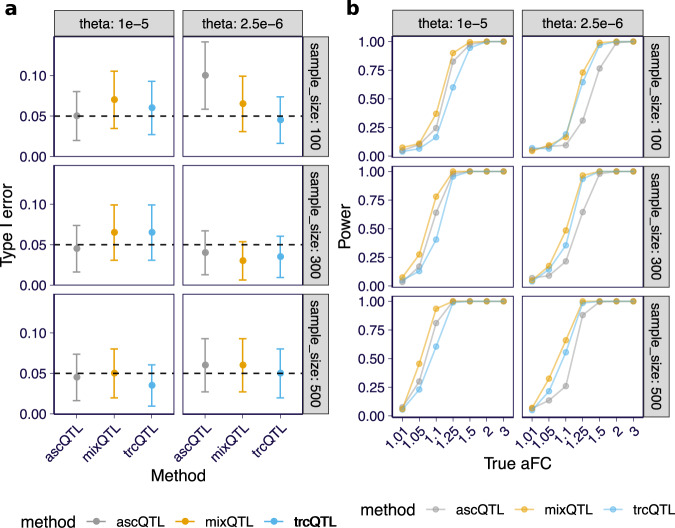
QTL mapping performance for mixQTL and approaches based on either total reads (trcQTL) or allele-specific reads (ascQTL) on simulated data. Each panel presents the results for two relative abundances of the gene, *θ*, and three sample sizes. **a** Type I error (*y*-axis) at a 5% significance level across methods (*x*-axis) are shown. The dashed line represents the desired error rate under the null hypothesis. The error bar indicates the 95% confidence interval of the estimated error rate from 200 replicates. **b** Power (*y*-axis) at a 5% significance level across methods under a range of true aFC values (*x*-axis) are shown. Power is defined as the fraction of eQTLs passing the significance threshold.

All three methods had calibrated type I errors (Fig. 2a and Supplementary Fig. 1). mixQTL outperformed both trcQTL and ascQTL in all simulation settings, demonstrating the benefits of combining total and allele-specific counts for cis-eQTL mapping (Fig. 2b and Supplementary Fig. 2).

The power of ascQTL was sensitive to the number of allele-specific reads, as expected. As shown in Fig. 2b, with *θ* controlling the expression level, ascQTL yielded much higher power for higher expression levels. In contrast, trcQTL was less sensitive to the number of reads observed under the range of read counts in our simulation settings. Such sensitivity differences between ascQTL and trcQTL are consistent with the nature of count data, where the magnitude of the noise is inversely related to the count.

### Combining total and allele-specific read count improves fine-mapping

**Fig. 3:**
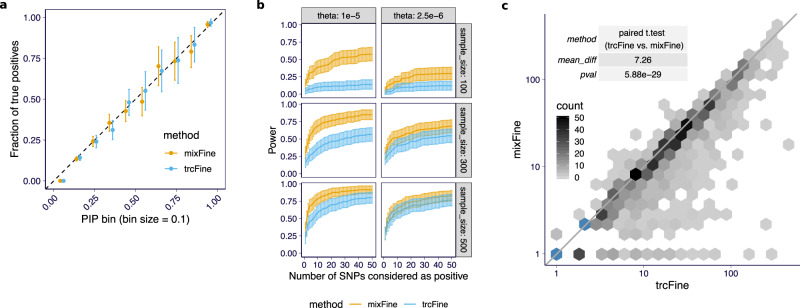
Fine-mapping performance of the combined (mixFine) and total read-based (trcFine) approaches on simulated data. **a** The observed fraction of true signals within SNPs binned by PIP are shown (aggregated across all simulation settings) for both mixFine (orange) and trcFine (blue). The plot is based on 10,211,200 simulations across the grid of simulation parameters. From left to right, the bin sizes for mixFine are 10,206,540, 2554, 742, 335, 234, 128, 57, 56, 67, 487 and the bin sizes for trcFine are 10,208,066, 1790, 495, 241, 152, 69, 52, 38, 48, 249. The error bars indicate the 95% confidence interval of the estimated fraction. **b** The power at a PIP cutoff (on *y*-axis) is plotted against the number of variants passing the PIP cutoff (on *x*-axis) for mixFine and trcFine. In each panel, the curve is based on 200 simulation replicates with 100 simulations having signals and 100 simulations being drawn from the null. The solid curves indicate the mean power (recall rate) among the 100 simulation replicates with signals and the error bars indicate the 95% confidence interval. **c** For the true signals captured in both mixFine and trcFine, the sizes of the 95% credible sets in the two methods are plotted (trcFine on *x*-axis and mixFine on *y*-axis). The table shows the average difference of the size (trcFine vs. mixFine) along with the *p*-value under paired *t*-test (two-sided). The color of a hexagonal bin indicates the count of data points in the bin. The blue bins have more than 50 counts.

To realistically simulate LD structure, we used the genotypes of European individuals from the 1000 Genomes projects phase 3^21^ within ±1 MB cis-windows of 100 randomly selected genes. We applied mixFine and trcFine (which uses total read counts only; Supplementary Notes 5.3) to the simulated data and characterized the fine-mapping results with two metrics: (1) power curve, defined as the proportion of detected variants among causal ones versus the number of detected variants, where detection was defined as the variant having posterior inclusion probability (PIP) > threshold (which is varied to get the desired number of detected SNPs); (2) the size of the 95% credible set (CS), which contains the causal variant. The PIPs of both trcFine and mixFine were consistent with the proportion of true causal variants within each PIP bin (Fig. 3a). By combining total and allele-specific reads, mixFine achieved higher power than trcFine (Fig. 3b and Supplementary Fig. 4) across almost all simulation settings. mixFine achieved the highest improvement relative to trcFine at a high expression level (*θ*), corresponding to high-quality allele-specific signals. The gain in power decreased with larger sample sizes. The increased power was also reflected in the number and size of 95% CSs containing the true signals. As shown in Fig. 3c and Supplementary Fig. 5, mixFine identified more true positive 95% CSs, and these 95% CSs were generally smaller than the ones of trcFine (paired *t*-test *p* = 5.88 × 10^−29^) demonstrating that mixFine can pinpoint causal SNPs more accurately.

Overall, the combined method was more powerful for identifying causal variants, which is consistent with recent reports^10,11^.

### Combining total and allele-specific read count improves prediction

Using the data from the fine-mapping simulation, we tested the performance of mixPred and trcPred (Supplementary Notes 5.3) on held-out test data. Specifically, we split each simulation replicate into training (4/5) and test (1/5) sets. We trained prediction models using training data and evaluated the prediction performance on test data using Pearson correlation between predicted and true responses. For each dataset, we repeated the splitting-training-evaluation procedure twice to reduce the stochasticity introduced by splitting.

**Fig. 4:**
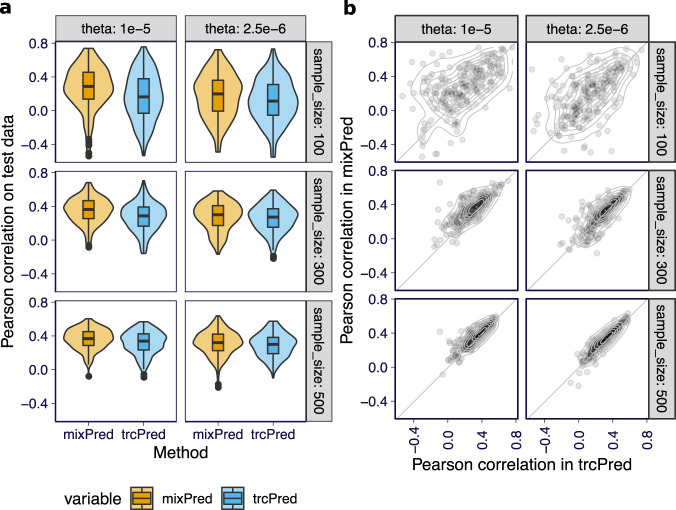
Prediction performance of the combined (mixPred) and total read-based (trcPred) methods on simulated data. **a** The overall distribution of Pearson correlations between predicted and observed total count abundance in log scale, i.e., ${\mathrm{log}}\,({Y}_{i}^{\,\text{total}\,}/{L}_{i})$, for mixPred (orange) and trcPred (blue) across all data splits are shown. For each panel, the plot is based on 200 simulation replicates. In the boxplots, the lower and upper hinges show the first and third quartiles and the middle line shows the median. The whiskers extend from the hinge to the maximum and minimum at most 1.5× the interquartile range. All data points beyond the end of the whiskers are plotted individually. **b** For each split, the prediction performance of mixPred (*y*-axis) is plotted against the prediction performance of trcPred (*x*-axis).

Overall, mixPred achieved higher prediction accuracy than trcPred (Fig. 4 and Supplementary Figs. 6 and 7). The gain in performance was more apparent when the expression level *θ* was higher and as a consequence the allele-specific count was larger.

### mixQTL outperforms standard eQTL mapping in GTEx data

Next, we compared mixQTL to the standard eQTL mapping approach (denoted here simply as eQTL) used by the GTEx consortium^13^, using 670 whole-blood RNA-seq samples from the v8 release (see Methods). We included variants within a ±1 Mb cis-window around the transcription start site of each gene. Although mixQTL can be applied to all genes regardless of the number of allele-specific counts, we focus on examining the benefit of integrating allele-specific information and therefore limit these comparisons to genes with sufficient allele-specific counts, based on the following criteria: (1) at least 15 samples having at least 50 allele-specific counts for each haplotype; and (2) at least 500 samples having a total read count of at least 100. Five thousand seven hundred and thirty four (28%) genes passed these filters. We then stratified these genes by their median expression level (read counts) into low, medium, and high expression tertiles. For genes with below-threshold allele-specific counts, the calculation can be performed using total read counts only, such that all genes considered using the standard approach are also tested in mixQTL. Performance for these genes was similar to the standard eQTL approach (Supplementary Fig. 8).

All three approaches mixQTL, aseQTL, and trcQTL were relatively well-calibrated when permuting data in four randomly selected genes (Supplementary Fig. 9). The estimated effect sizes were consistent with allelic fold change estimates from the main GTEx v8 analysis (Supplementary Fig. 10).

To further compare the performance of the methods, we used eQTLGen^14^, a large-scale meta-analysis of over 30,000 blood samples, as our “ground truth” eQTL discovery reference (Supplementary Notes 8). We selected a random subset of 100,000 variant/gene pairs tested by eQTLGen with FDR < 0.05 as the set of “ground truth” eQTLs. We also selected a random set of 100,000 variant/gene pairs with *p* > 0.50 as a background set of “non-significant” eQTLs. Among these pairs, 96,660 and 78,691 of the “ground truth” and “non-significant” pairs had matching data in GTEx.

**Fig. 5:**
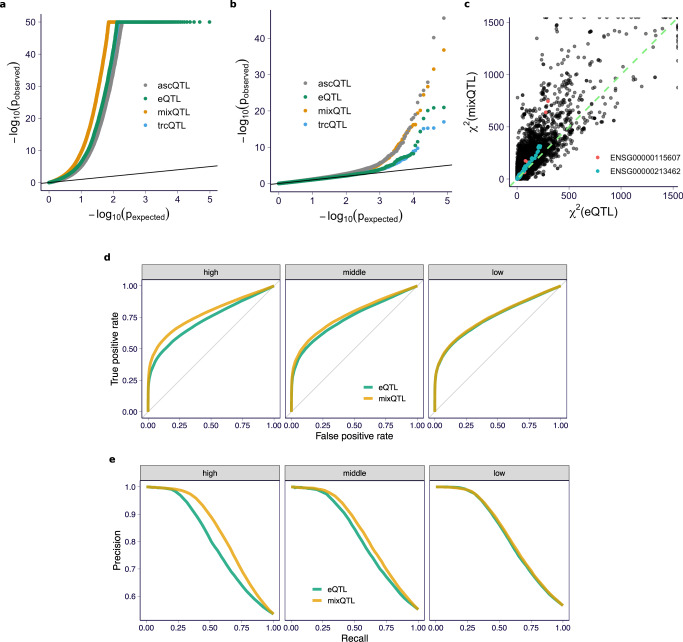
Performance of mixQTL on GTEx v8 whole-blood RNA-seq. **a** QQ-plot of nominal *p*-values for a random subset (size = 96,660) of cis-eQTLs (FDR < 0.05) reported in eQTLGen. **b** QQ-plot of nominal *p*-values for a random subset (size = 78,691) of variant/gene pairs with *p*-value > 0.5 in eQTLGen. **c** *χ*^2^ statistics from eQTL analysis (*x*-axis) and mixQTL analysis (*y*-axis) among a random subset (size = 96,660) of cis-eQTLs (FDR < 0.05) reported in eQTLGen. Two randomly selected genes (ENSG00000115607 and ENSG00000213462) are highlighted in red and green, respectively. **d**, **e** ROC and PR curves for mixQTL and the standard eQTL method measured in eQTLGen. Each panel shows the results of genes stratified by expression level tertiles.

For the “ground truth” eQTLs, mixQTL yielded more significant *p*-values compared to the standard eQTL, ascQTL, and trcQTL approaches (Fig. 5). The “non-significant” variant/gene pairs showed moderate enrichment for small *p*-values for all methods (Fig. 5b), likely reflecting a combination of false negatives in eQTLGen and potential false positives in our analysis. Overall, we found that mixQTL achieves increased power compared to standard eQTL mapping on real data for the set of genes with sufficient total and allele-specific read counts.

As an intuitive measure of improved performance, we estimated the effective sample size gain of mixQTL compared to standard eQTL mapping as the median of the ratio between mixQTL *χ*^2^ statistics and eQTL *χ*^2^ statistics. mixQTL showed a 29% increase in effective sample size compared to the standard eQTL mapping approach (Fig. 5c).

To account for the trade-off between true- and false-positive rates, as well as between precision and power, we used receiver operating characteristic (ROC) and precision-recall (PR) curves to compare the performance of mixQTL and standard eQTL approaches using the eQTLGen “ground truth” and “non-significant” eQTLs. We found that mixQTL achieves higher performance in both ROC (Fig. 5d) and PR curves (Fig. 5e). Consistent with simulation results, this gain is more significant for genes with higher expression levels.

To determine whether the eQTLGen-based analysis above depended on the selected random subset of cis-eQTLs, we repeated the analysis for multiple samplings of eQTLGen results and found no substantive differences in the results.

### mixQTL is scalable to full GTEx eQTL analysis

To compare the performance and computational cost of mixQTL and the existing QTL mapping approaches which can leverage both total and allele-specific counts, we ran RASQUAL on two of the GTEx tissues, kidney cortex (sample size = 73; a subset of 4596 genes) and whole blood (a subset of 192 genes; Supplementary Notes 9). We observed concordant effect size estimates (Supplementary Fig. 11A). As expected, because RASQUAL models counts directly instead of approximating them with a log-linear model, it yielded more significant results than mixQTL (Supplementary Fig. 11B). On average, RASQUAL took 47 seconds per gene in kidney cortex and 826 seconds per gene in whole blood whereas mixQTL took 0.065 seconds (723 times faster) and 0.33 seconds (2480 times faster), respectively.

Given this computational efficiency, we decided to run mixQTL on the 49 tissues from the GTEx v8 release. This corresponded to 15,201 samples in total, and took ~54 CPU hours in total (without permutations).

mixQTL’s runtime scaled linearly as a function of sample size (Supplementary Fig. 12A), with the tissue with the largest sample size (skeletal muscle, *n* = 706) taking 0.34 seconds per gene on average.

At FDR cutoff 0.05, on average, mixQTL identified 1440 more genes and about 618,000 more eQTLs than the standard eQTL approach (Supplementary Fig. 12B and C). The full summary statistics of mixQTL are publicly available (Supplementary Data 1).

### Fine-mapping and prediction model building in GTEx data

We applied mixFine to the GTEx v8 whole-blood RNA-seq data, using the same subset of genes with high expression and allelic counts that were used in the comparison of mixQTL vs. standard eQTL approach above. We compared mixFine to the SuSiE fine-mapping approach^19^, applied to inverse normal transformed expression values in the standard eQTL mapping pipeline^13^. We corrected for sex, five genetic principal components, WGS platform, WGS library prep protocol (PCR), and 60 PEER factors. We refer to the latter as the “standard approach” below for simplicity.

To compare the power of causal variant detection, we performed a subsampling analysis on a random subset of 1000 genes. First, we defined “consensus SNPs” as the variants with PIP > 0.5 in both mixFine and the “standard approach” using all samples. Similarly, a variant was defined as “top SNP” if it was the most significant variant within the 95% CS for both mixFine and the “standard approach”. Then, we compared how well the “consensus SNPs” and “top SNPs” were detected by mixFine and the standard fine-mapping approach using only a subset of samples. We subsampled to 90%, 80%, ⋯ , 30% of samples, and repeated each random subsampling step 10 times.

**Fig. 6:**
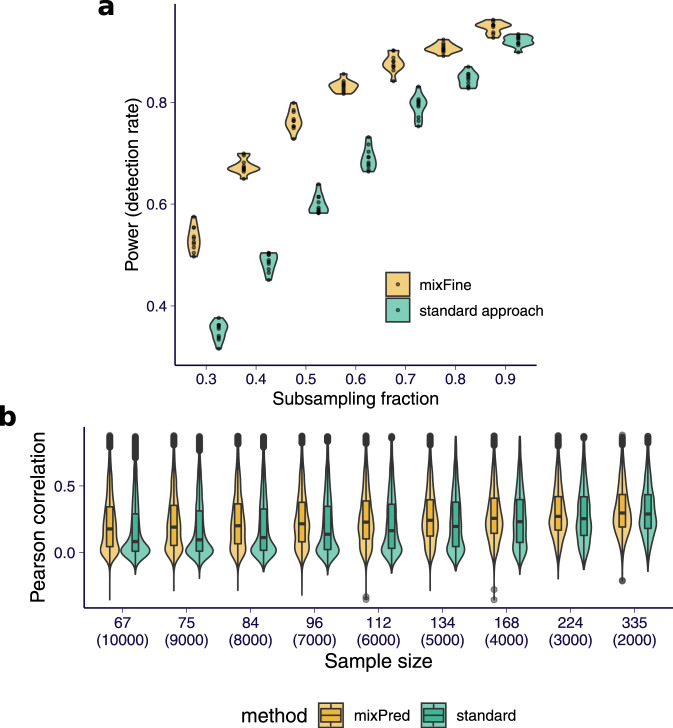
Performance of mixFine and mixPred on GTEx v8 whole-blood RNA-seq. **a** The fraction of detected "consensus SNPs'' among all 272 "consensus SNPs'' in full data as a function of subsampling level, for mixFine and the standard approach, are shown. The subsampling analysis are repeated 10 times. The plot shows the results of all the ten replicates. **b** The Pearson correlation between observed and predicted expression across all models trained from 1000 genes are shown. "Standard'' corresponds to the elastic net model as implemented in ref. ^1^. The results are stratified by sample size used for training. For each sample size, the distribution of the Pearson correlation across all cross-validation folds and genes are shown (the corresponding total number of observations is shown in the parentheses). In the boxplots, the lower and upper hinges show the first and third quartiles and the middle line shows the median. The whiskers extend from the hinge to the maximum and minimum at most 1.5× the interquartile range. All data points beyond the end of the whiskers are plotted individually.

Among the 1000 genes, there were 272 “consensus SNPs” being identified in the full data. At each subsampling level, mixFine, on average, detected more “consensus SNPs” than the standard approach (Fig. 6a) and performance improved most on the more highly expressed genes (top tertile) (Supplementary Fig. 13). Moreover, mixFine detected “top SNPs” in 95% CSs with an average size of 9.5 variants, whereas the corresponding 95% CS from the standard approach had 14.6 variants on average (Supplementary Fig. 14). Furthermore, since the power gain would be more apparent in small sample sizes, we ran mixFine and standard eQTL approach in 26 GTEx v8 tissues with sample size <260. We examined the enrichment of the top QTL and PIP in different functional annotations, including regulatory element annotations, candidate cis-regulatory elements (cCREs)^22^, and the GWAS catalog (Supplementary Notes 10). We found that the variants with the most significant mixQTL *p*-value or the highest mixFine PIP were more enriched in GWAS catalog variants and cCREs than the standard approach. We found enrichment of enhancer, promoter, and transcription factor binding sites but the difference in enrichment between mixQTL and standard QTL methods was not significant (Supplementary Fig. 16). The reduced enrichment compared to cCREs are likely due to the fact that we used tissue-specific annotations for cCREs and cross-tissue annotations for enhancers, promoters, and TFs. These results indicate that, when sufficient counts are available, mixFine, the multi-SNP model combining total and allele-specific counts, can better pinpoint causal cis-eQTLs than the standard approach on real data.

To compare the performance of mixPred and the standard method on real data, we implemented a cross-validated evaluation pipeline where we split the GTEx v8 whole-blood data into *k* folds. At each fold, we trained the prediction model using one fold of the data and evaluated the performance (by Pearson correlation between predicted and observed ${\mathrm{log}}\,({Y}_{i}^{\,\text{total}\,}/{L}_{i})$) on the remaining (*k* − 1) folds. We applied this evaluation pipeline to mixPred and the standard approach (elastic net as in ref. ^1^) on the same 1000 genes as the subsampling analysis with *k* = 10, 9, …, 2 (corresponding to sample size = 67, 75, ⋯ , 335). At the same sample size, we observed, on average, significantly higher performance in mixPred as compared to the standard approach, and the performance gain was greater for smaller sample sizes (Fig. 6b and Supplementary Table 1).

## Discussion

We proposed a unified framework that integrates both allele-specific and total read counts to estimate genetic cis-regulatory effects, resulting in improved eQTL mapping, fine-mapping, and prediction of gene expression traits. Our suite of tools (mixQTL, mixFine, and mixPred) can be scaled to much larger sample sizes (thousands) due to the underlying log-linear approximation. By assuming weak multiplicative genetic effects consistent with observations (most estimated log allelic fold changes of cis-eQTLs have a median absolute value of 0.153 and a 95th percentile of 0.845 (Supplementary Fig. 15)), we transform the observed read counts into two approximately independent quantities: allelic imbalance and total read count. Leveraging this independence, we developed computationally efficient approaches that integrate both allele-specific and total reads.

Specifically, mixQTL estimates the genetic effect separately for allelic imbalance and total read counts, and combines the resulting statistics via meta-analysis. These calculations have computationally efficient closed-form solutions, enabling their use in permutation schemes applied to compute FDR in eQTL mapping^23–25^.

Furthermore, the simple multi-SNP extension and the approximate independence of the terms enable use of a two-step inference procedure. In the first step, the allelic imbalance and total read count are scaled such that the error terms have the same variance. And in the second step, given their approximate independence, the pair of equations (from allelic imbalance and total counts) can simply be input into existing fine-mapping and prediction algorithms.

We showed through simulations and applications to GTEx v8 data that our suite of methods outperforms methods that rely on total read counts alone. Compared to existing QTL mapping methods that integrate total and allele-specific reads, such as RASQUAL^9^, mixQTL has slightly lower power (Supplementary Fig. 11B). This is expected since RASQUAL models count data directly and mixQTL relies on approximations. However, the computational burden of RASQUAL is prohibitive for large datasets. In practice, the most suitable approach will depend on computational capacity and sample sizes. For datasets with small sample sizes (e.g., fewer than 100 samples), RASQUAL or WASP remain preferable. The computational efficiency of mixQTL makes it applicable to large sample sizes, and, moreover, enables using the mixQTL model in place of the standard eQTL mapping approach that relies on inverse normal transformed counts.

Given the unified modeling framework and computationally scalable tools proposed here, we anticipate that combining total and allele-specific read counts will find widespread use for cis-QTL mapping, fine-mapping, and prediction of gene expression.

## Methods

### Notation and terminology

It is described in Table 1.

**Table 1:** Summary of notation and terminology used in the paper.

Notation	Description	Synonym in text	Observable
*i*	Individual index.	—	—
*h*	Haplotype index, with *h* = 1, 2 for diploid.	—	—
${X}_{i}^{h}$	Alternative allele count (0 or 1) of the variant linking to the gene haplotype *h*.	Allelic dosage	Yes
*L*_*i*_	The total number of reads in the RNA-seq library.	Library size	Yes
${Y}_{i}^{h}$	Count of reads originated from gene haplotype *h*.	Haplotypic (read) count	No
${Y}_{i}^{(h)\,\text{obs}\,}$	Allele-specific read count that gets aligned to the gene haplotype *h*.	Allele-specific (read) count	Yes
${Y}_{i}^{\,\text{total}\,}$	Total count of reads originated from any of the two gene haplotypes (sum).	Total (read) count	Yes
*θ*_0,*i*_	The abundance of the gene haplotype relative to the total transcriptome when the linked causal variants are all in reference alleles	Baseline (relative) abundance	No
${\theta }_{i}^{h}$	The abundance of the gene haplotype *h* relative to the total transcriptome in individual *i*	(Relative) abundance; expression level^a^	No
*β*	The log fold change of gene haplotype abundance when linking to alternative allele relative the reference allele	Allelic fold change (aFC) in natural log scale	No
$\frac{{Y}_{i}^{(1)\,\text{obs}\,}}{{Y}_{i}^{(2)\,\text{obs}\,}}$	The ratio of the allele-specific counts between two haplotypes	Allelic imbalance	Yes
${Y}_{i}^{\,\text{trc}\,}$	Shorthand of the term $\mathrm{log}\,\frac{{Y}_{i}^{\,\text{total}\,}}{2{L}_{i}}$.	—	—
${Y}_{i}^{\,\text{asc}\,}$	Shorthand of the term $\mathrm{log}\,\frac{{Y}_{i}^{(1)\,\text{obs}\,}}{{Y}_{i}^{(2)\,\text{obs}\,}}$	—	—
*θ*	Only used in simulation where *θ* = E(*θ*_0,*i*_)	expression level^b^	—

The "Description” column contains a brief definition of each "Notation”, and the "Synonym in text” column contains the corresponding terminology used in the text. The "Observable” column indicates whether the entity is an observable variable or not.

^a,b^Expression level does not strictly refer to ${\theta }_{i}^{h}$ or E(*θ*_0,*i*_), but, more generally, it refers to the abundance of the gene transcripts relative to the transcriptome.

### Statistical model of cis-regulation

For individual *i*, let ${X}_{i}^{1}$ and ${X}_{i}^{2}$ be the number of alternative alleles in each of the two haplotypes at the variant of interest. Let ${Y}_{i}^{1}$ and ${Y}_{i}^{2}$ be the number of reads coming from each of the two haplotypes (i.e., haplotypic counts; in practice, these quantities are unobserved) and *L*_*i*_ the library size for individual *i*. As proposed in ref. ^12^, we use the concept of allelic fold change (aFC) to represent the genetic effect on cis-expression. We denote *θ*_0,*i*_ as the baseline abundance of the transcripts originating from each of the gene haplotype without considering genetic effect. Let *β* be the genetic effect of a variant of interest, which is defined as the log fold change relative to the reference allele. Then, the transcript abundance of each haplotype *h* after accounting for the genetic effect is ${\theta }_{i}^{h}={\theta }_{0,i}\times g(\beta ,{X}_{i}^{h})$ where $g(\beta ,{X}_{i}^{h})$ is *e*^*β*^ if ${X}_{i}^{h}$ is the alternative allele; otherwise $g(\beta ,{X}_{i}^{h})=1$. We model read count ${Y}_{i}^{h}$ as

5 $${\mathrm{log}}\,{Y}_{i}^{h}| {L}_{i},{\theta }_{i}^{h} \sim N({\mathrm{log}}\,({L}_{i}{\theta }_{i}^{h}),{\tau }_{i}^{h}).$$

In an RNA-seq experiment, a fraction of reads contribute to allele-specific read counts. Let *α*_*i*_ denote the fraction of allele-specific reads in individual *i*, which depends on the number of heterozygous sites within the transcript. Instead of observing haplotypic counts ${Y}_{i}^{1}$ and ${Y}_{i}^{2}$, we observe total read count ${Y}_{i}^{\,\text{total}\,}$ and gene-level allele-specific read counts ${Y}_{i}^{(1)\,\text{obs}\,}$ and ${Y}_{i}^{(2)\,\text{obs}\,}$. Similarly, we further assume that the baseline abundance of allele-specific reads per haplotype is *θ*_0,*i*_ × *α*_*i*_, so we have

6 $${\mathrm{log}}\,{Y}_{i}^{(1)\,{\text{obs}}\,}| {L}_{i},{\theta }_{i}^{1},{\alpha }_{i}\ \sim N({\mathrm{log}}\,({\alpha }_{i}{L}_{i}{\theta }_{i}^{1}),{\tau }_{i}^{(1)})$$

7 $$\begin{array}{*{20}{l}}{\mathrm{log}}\,{Y}_{i}^{(2)\,{\text{obs}}\,}| {L}_{i},{\theta }_{i}^{2},{\alpha }_{i}\hfill & \sim \hfill & N({\mathrm{log}}\,({\alpha }_{i}{L}_{i}{\theta }_{i}^{2}),{\tau }_{i}^{(2)})\hfill\\ {\mathrm{log}}\,{Y}_{i}^{\,\text{total}\,}| {L}_{i},{\theta }_{i}^{1},{\theta }_{i}^{2}\hfill & = \hfill& {\mathrm{log}}\,({Y}_{i}^{1}+{Y}_{i}^{2})| {L}_{i},{\theta }_{i}^{1},{\theta }_{i}^{2}\hfill\end{array}$$

8 $$\sim N({\mathrm{log}}\,[{L}_{i}({\theta }_{i}^{1}+{\theta }_{i}^{2})],{\tau }_{i})$$

### Linearizing the model by approximation

Based on the model described above along with approximations under weak effect assumptions, we propose the following linear mixed effects model (see Supplementary Notes 2 for derivation):

9 $${{\underbrace{{\mathrm{log}}\,\frac{{Y}_{i}^{\text{total}}}{2{L}_{i}}}}\atop {{Y}_{i}^{\text{trc}}}}={\mu }_{0}+z_i+{{\underbrace{\frac{{X}_{i}^{1}+{X}_{i}^{2}}{2}}} \atop {{X}_{i}^{\text{trc}}}}\beta +{\epsilon }_{i}^{\text{trc}\,}$$

10 $${{\underbrace{{\mathrm{log}}\,\frac{{Y}_{i}^{(1)\text{obs}}}{{Y}_{i}^{(2)\text{obs}}}}}\atop {\begin{array}{c}{Y}_{i}^{\text{asc}}\end{array}}}={{\underbrace{({X}_{i}^{1}-{X}_{i}^{2})}}\atop{\begin{array}{c}{X}_{i}^{\text{asc}}\end{array}}}\beta +{\epsilon }_{i}^{\text{asc}\,}$$

11 $${z}_{i} \sim N(0,{\sigma }_{0}^{2}),\,{\epsilon }_{i}^{\,\text{trc}\,} \sim N(0,\frac{{\sigma }^{2}}{{Y}_{i}}),\,{\epsilon }_{i}^{\,\text{asc}\,} \sim N\left(0,{{\underbrace{\frac{{\sigma }^{2}{Y}_{i}^{(1)}{Y}_{i}^{(2)}}{{Y}_{i}^{(1)}+{Y}_{i}^{(2)}}}}\atop{\begin{array}{c}{\sigma }^{2}/{w}_{i}\end{array}}}\right),$$

where *z*_*i*_ is the individual-level random effect capturing the between-individual variation of *θ*_*i*,0_. Notice that the individual-level random effect cancels out when we take the difference between the two log-scale allele-specific read counts (allelic imbalance in log scale). The scaling of *ϵ*^trc^ and *ϵ*^asc^ in Eq. (11) is to ensure that variance of read count scales linearly with the magnitude of read count (see Supplementary Notes 1.2). In other words, this model ensures Var(*Y*) ≈ constant × E(*Y*), such that over-dispersion is implicitly taken into account.

Since ${\epsilon }_{i}^{\,\text{asc}\,}$ is approximately independent to ${\epsilon }_{i}^{\,\text{trc}\,}$ (see Supplementary Notes 4), ${\epsilon }_{i}^{\,\text{trc}\,}$ and *z*_*i*_ can be merged into one term ${\widetilde{z}}_{i}$. So, we can further simplify Eqs. (9), (10) as

12 $${Y}_{i}^{\,\text{trc}}={\mu }_{0}+{X}_{i}^{\text{trc}}{\beta }^{\text{trc}}+{\widetilde{z}}_{i},{\widetilde{z}}_{i} \sim N(0,{\widetilde{\sigma }}_{0}^{2})$$

13 $${Y}_{i}^{\,\text{asc}}={X}_{i}^{\text{asc}}{\beta }^{\text{asc}}+{\epsilon }_{i}^{\text{asc}},{\epsilon }^{\text{asc}} \sim N(0,{\sigma }^{2}/{w}_{i})$$

Equations (12), (13) are applicable to both single SNP and multi-SNP scenarios. In the single-SNP case, *X*_*i*_ and *β* are scalars, and in the multi-SNP case, *X*_*i*_ and *β* are replaced by vectors including all SNPs within the cis-window (see Supplementary Notes 3).

### Numerically efficient QTL mapping leveraging approximate independence of allelic imbalance and total read count

The likelihood function corresponding to the proposed model in Eqs. (12), (13) approximately takes the form

$$\mathop{\prod}\limits_{i}\Pr(Y_{i}^{{\rm{total}}}|u_{0},\widetilde{\sigma}^{2}_{0},\beta) \cdot \Pr \left(\frac{Y_{i}^{(1){\rm{obs}}}}{Y_{i}^{(2){\rm{obs}}}}|\sigma^{2},\beta\right),$$

factoring into total read count and allelic imbalance components. (see Supplementary Notes 2.2). This means that the likelihood for total read count and the ratio of allele-specific read counts provide approximately independent information on *β*, and enables us to solve each component separately and combine the results via meta-analysis (standard approach with independent studies^26^). Specifically, we fit *β*^trc^ and *β*^asc^ using total and allele-specific observations as two separate linear regression problems, and meta-analyze the results using inverse-variance weighting (see details in Supplementary Notes 4.2).

### Two-step inference procedure for multi-SNP model

The prediction and fine-mapping problems both rely on the linearized model Eqs. (12), (13), but with different objectives. For prediction, the objective is to find the best predictor, whereas for fine-mapping, the objective is to infer whether *β*_*k*_ is non-zero. Existing solvers for both prediction and fine-mapping use total read information only and assume that data (*X*, *y*) follows the model *y* = *Xβ* + *ϵ*, where the noise term *ϵ* is independent across the rows of the data matrix. We will refer to this model as the ‘canonical’ linear model. We propose a two-step inference procedure that first processes the data such that it approximates *y* = *Xβ* + *ϵ*, and then uses existing solvers for prediction and fine-mapping problems, respectively.

For the first step, we process total and allele-specific reads separately to fit the ‘canonical’ linear model. Specifically, we estimate *σ*^2^ from (*Y*^asc^, *X*^asc^) based on Eq. (13) by further assuming the genetic effect as random effect and estimating *σ*^2^ using R package EMMA^27^. And similarly, based on Eq. (12) and the random effect assumption, we estimate ${\widetilde{\sigma }}_{0}^{2}$ from (*Y*^trc^, *X*^trc^). To account for the intercept term *μ*_0_ in Eq. (12), we center *Y*^trc^ and *X*^trc^ by subtracting the mean values across all samples and then scale the centered (*Y*^trc^, *X*^trc^) by $1/{\widehat{\widetilde{\sigma }}}_{0}$. And similarly, we scale (*Y*^asc^, *X*^asc^) by $w/\hat{\sigma }$. These linear transformations ensure that the transformed $({\tilde{Y}}^{\text{trc}},{\tilde{X}}^{\text{trc}})$ and $({\tilde{Y}}^{\text{asc}},{\tilde{X}}^{\text{asc}})$ both approximately follow *Y* = *Xβ* + *ϵ*. The implementation details are described in Supplementary Notes 5. At the second step, we concatenate the transformed data from both total and allele-specific read counts as $(\tilde{Y},\tilde{X})$, which is compatible with existing solvers for prediction and fine-mapping problems.

### Adjusting for covariates

When analyzing real data, we need to take covariates such as sex, batch effect, population stratification into account. Here, we adapt the procedure which has been proposed previously^12^. We regress out the effect of covariates beforehand and use the residual as the response in both QTL mapping and fitting multi-SNP model. Specifically, let *c*_1_, ⋯ , *c*_*K*_ denote the *K* covariates to be considered. We first regress *Y*^trc^ against *c*_1_, ⋯ , *c*_*K*_ jointly and select the covariates with nominally significant coefficients (*p* < 0.05). Then we regress *Y*^trc^ against the selected covariates jointly and set the residuals as the adjusted *Y*^trc^ for QTL mapping and multi-SNP inference downstream.

### Simulation scheme

We simulate RNA-seq reads with total and allele-specific readouts as sketched in three steps in Fig. 1. In step 1, we specify, for each individual *i*, the position of heterozygous sites within the gene body. The expected read count from each haplotype transcripts, $\,\text{E}\,({Y}_{i}^{h})$, is determined by the RNA-seq library size *L*_*i*_, the baseline abundance of the transcript *θ*_0,*i*_, and the genetic effect *β*. In step 2, given the expected haplotypic count, we draw ${Y}_{i}^{h}$ from Negative Binomial to model the variation among count data. In step 3, we position the reads randomly along the gene body and readout observed allele-specific count ${Y}_{i}^{(h)\,\text{obs}\,}$ by counting the number of reads overlapping heterozygous sites simulated in step 1. The total read count readout is ${Y}_{i}={Y}_{i}^{1}+{Y}_{i}^{2}$, which is independent of the number of heterozygous sites.

To survey a wide range of parameters, we simulate data with a grid of parameters. We vary sample size among 100, 200, ..., 500. At library size around 90 million, we vary the level of *θ*_0,*i*_ to cover the gene with different expression levels, among 5 × 10^−5^, 2.5 × 10^−5^, 1 × 10^−5^, 5 × 10^−6^, 2.5 × 10^−6^, 1 × 10^−6^. The genetic effect, aFC, is set to 1 (null), 1.01, 1.05, 1.1, 1.25, 1.5, 2, 3 in the single-SNP model. For the multi-SNP scenario, we set the number of causal SNPs between 1 and 3 with heritability from 0.2 to 0.55. The number of polymorphic sites within the gene body is centered around 10 with minor allele frequency from 0.05 to 0.3. A detailed description and parameter settings are provided in the Supplementary Notes 6.

### Analysis of GTEx v8 data

We downloaded the phased genotypes, total read count matrix, and variant-level allele-specific read counts for whole blood from GTEx release 8^13^ via dbGaP (accession number phs000424.v8.p2). To obtain gene-level read counts, we summed over allele-specific counts at all the heterozygous sites for each gene haplotype. We also obtained library size, sex, and genotype PCs from GTEx v8. For comparisons with the inverse normalization-based approach, we also downloaded normalized expression matrices.

Similarly to the GTEx v8 analyses^13^, we restricted the analysis to the cis-regulatory window defined as 1Mbp up/downstream of the transcription start site of each gene.

To obtain the PEER factors for mixQTL analysis, we ran peertool^28^ on a matrix with value $\mathrm{log}\,(\frac{{Y}_{i,g}}{2{L}_{i}})$ for individual *i* and gene *g* (imputed by k-nearest neighbors if *Y*_*i*,*g*_ is zero using impute::impute.knn in R).

We considered very large allele-specific counts to be likely alignment artifacts and removed individuals with allele-specific read counts greater than 1000. To further limit the influence of large count outliers on the estimated log fold change, ${\hat{\beta }}^{\text{asc}}$, we set the largest weight ${\left(\frac{1}{{Y}_{\,}^{(1)\text{obs}}}+\frac{1}{{Y}_{\,}^{(2)\text{obs}}}\right)}^{-1}$ to be at most *K* fold to the smallest one, where $K=\min (10,\,\text{sample size}\,/10)$.

Specific analyses focused on high or low expression were performed with different gene filtering criteria as stated in the Results section.

For analyses of the full GTEx v8 dataset, we built a data analysis pipeline at https://github.com/liangyy/mixqtl-gtex/tree/master/mixqtl which relied on the tensorQTL implementation of mixQTL. We included all genes regardless of expression level and analyzed the 22 autosomes for each of the 49 tissues. Specifically, since mixQTL can only work with non-zero total read count, we imputed the samples with missing total read count as 1. And in the mixQTL call, all total read counts were included and all allele-specific counts with more than 15 reads (on both haplotypes) were included.

### Reporting summary

Further information on research design is available in the Nature Research Reporting Summary linked to this article.

## Supplementary information

Supplementary Information

Peer Review

Reporting Summary

Description of Additional Supplementary Fiies

Supplementary Data 1

## Data Availability

The Genotype-Tissue Expression (GTEx) project’s raw whole-transcriptome and -genome sequencing data are available via dbGaP accession number phs000424.v8.p2. All processed GTEx data are available via the GTEx portal (http://gtexportal.org/). The download links to the mixQTL full summary statistics for 49 GTEx tissues are listed in Supplementary Data 1.

## Code availability

Softwares mixQTL, mixFine, and mixPred in R https://github.com/hakyimlab/mixqtl^15^. A reproducible pipeline for the simulated data and some GTEx data analysis https://github.com/liangyy/mixqtl-pipeline^16^. A reproducible pipeline for the massive GTEx data analysis https://github.com/liangyy/mixqtl-gtex^17^. A GPU-based implementation embedded in tensorQTL https://github.com/broadinstitute/tensorqtl^18^.
