# Supplementary material for: A scalable unified framework of total and allele-specific counts for cis-QTL, fine-mapping, and prediction
Source: Nat Commun. 2021 Mar 3;12:1424. doi: 10.1038/s41467-021-21592-8 (PMC7930098; doi:10.1038/s41467-021-21592-8)
Supplement: Supplementary file 1 — Supplementary Information [file 41467_2021_21592_MOESM1_ESM.pdf]

Supplementary Information

# A scalable unified framework of total and allele-specific counts for cis-QTL, fine-mapping, and prediction

Yanyu Liang[1,*] François Aguet[2] Alvaro Barbeira[1] Kristin Ardlie[2]
Hae Kyung Im[1,*]

**1** Section of Genetic Medicine, The University of Chicago, Chicago, IL, USA

**2** The Broad Institute of MIT and Harvard, Cambridge, MA, USA

***** Correspondence to yanyul@uchicago.edu and haky@uchicago.edu

# List of Supplementary Figures


1 Type I error of mixQTL, ascQTL, and trcQTL on the full grid of simulations . . . . . . . . . . . . . . 4
2 Power of mixQTL, ascQTL, and trcQTL on the full grid of simulations . . . . . . . . . . . . . . . . . . 5
3 Difference between $\hat{\beta}$ and true $\beta$ of mixQTL, ascQTL, and trcQTL on the full grid of simulations . . . 6
4 Power curves of mixFine and trcFine on the full grid of simulations . . . . . . . . . . . . . . . . . . . . 7
5 Distribution of the positive 95% CS's which contain causal variants in mixFine and trcFine on the full grid of simulations . . . . . . . . . . . . . . . . . . . . . . . . . . . . . . . . . . . . . . . . . . . . 8
6 Distribution of Pearson correlations between predicted and observed expression level (in the scale $\log(Y_i^{\text{total}}/L_i)$) for mixPred and trcPred on the full grid of simulations . . . . . . . . . . . . . . . . . . 9
7 Pairwise comparison of prediction performance of mixPred and trcPred on the full grid of simulations 10
8 The performance of trcQTL and the standard eQTL approach on genes with low total read counts . . 11
9 QQ-plot of nominal p-values from ascQTL and trcQTL on four randomly selected genes in GTEx v8 whole blood RNA-seq . . . . . . . . . . . . . . . . . . . . . . . . . . . . . . . . . . . . . . . . . . 12
10 Comparison of aFC estimates from GTEx v8 and the estimated allelic fold change of ascQTL, trcQTL, and mixQTL . . . . . . . . . . . . . . . . . . . . . . . . . . . . . . . . . . . . . . . . . . . . . 13
11 The performance of RASQUAL in GTEx v8 kidney cortex RNA-seq . . . . . . . . . . . . . . . . . . 14
12 Running mixQTL on the full GTEx v8 data . . . . . . . . . . . . . . . . . . . . . . . . . . . . . . . 15
13 The performance of mixFine on GTEx v8 whole blood RNA-seq stratified by expression level . . . . . 16
14 The performance of mixFine on GTEx v8 whole blood RNA-seq on pinpointing the "top" SNPs . . . 16
15 The estimated cis-eQTL effect size in GTEx v8 whole blood . . . . . . . . . . . . . . . . . . . . . . . 17
16 Enrichment in functional annotation for GTEx v8 tissues . . . . . . . . . . . . . . . . . . . . . . . . 18


# List of Supplementary Tables


1 The pairwise comparison of the prediction performance between mixPred and the standard approach based on the cross-validated evaluation . . . . . . . . . . . . . . . . . . . . . . . . . . . . . . . . 19


# Contents of Supplementary Notes


**1 Statistical model for read count** **19**

1.1 Overview . . . . . . . . . . . . . . . . . . . . . . . . . . . . . . . . . . . . . . . . . . . . . 20
1.2 Parameterizing $\tau$ to weight total and AS count properly . . . . . . . . . . . . . . . . . . . . . . . 20

**2 Single-SNP model** **20**

2.1 From likelihood to linear mixed model . . . . . . . . . . . . . . . . . . . . . . . . . . . . . . . . . 20
2.2 Simplifying the model . . . . . . . . . . . . . . . . . . . . . . . . . . . . . . . . . . . . . . . . . 22

**3 Generalizing to multi-SNP model** **22**

**4 QTL mapping procedure** **23**

4.1 Converting the problems into two linear regressions . . . 23

4.2 Meta-analysis for QTL mapping . . . 24

**5 Inference procedure for multi-SNP model** **24**

5.1 Motivating two-step inference procedure . . . 24

5.2 Inferring $\widetilde{\sigma}_0^2$ and $\sigma^2$ . . . 25

5.3 Data transformation and inference . . . 25

**6 Simulating RNA-seq reads** **25**

**7 Pseudocode on solving trcQTL and ascQTL in matrix form** **27**

**8 Evaluating QTL mapping performance using eQTLGen results** **28**

8.1 Comparing the effective sample size . . . 29

8.2 Drawing receiver operating characteristic and precision-recall curves . . . 29

**9 Running RASQUAL on GTEx data** **29**

**10 Examining the enrichment in functional annotations** **29**

## Supplementary Figures and Tables

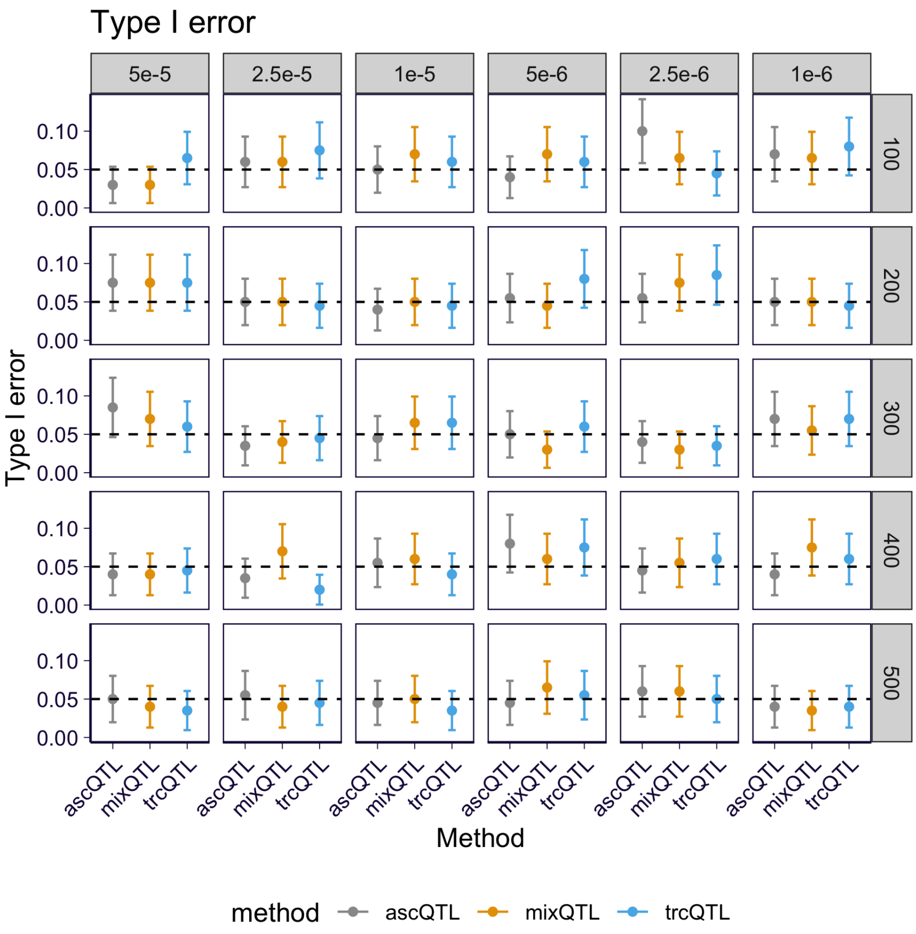


**Supplementary Figure 1. Type I error of mixQTL, ascQTL, and trcQTL on the full grid of simulations.** Each panel shows results on data simulated under a pair of $\theta$ (relative abundance in the simulation, by column) and sample size (by row). The error rate under significance level $\alpha = 0.05$ from 200 replicates is shown. The error bar indicates the 95% confidence interval of the estimated error rate.

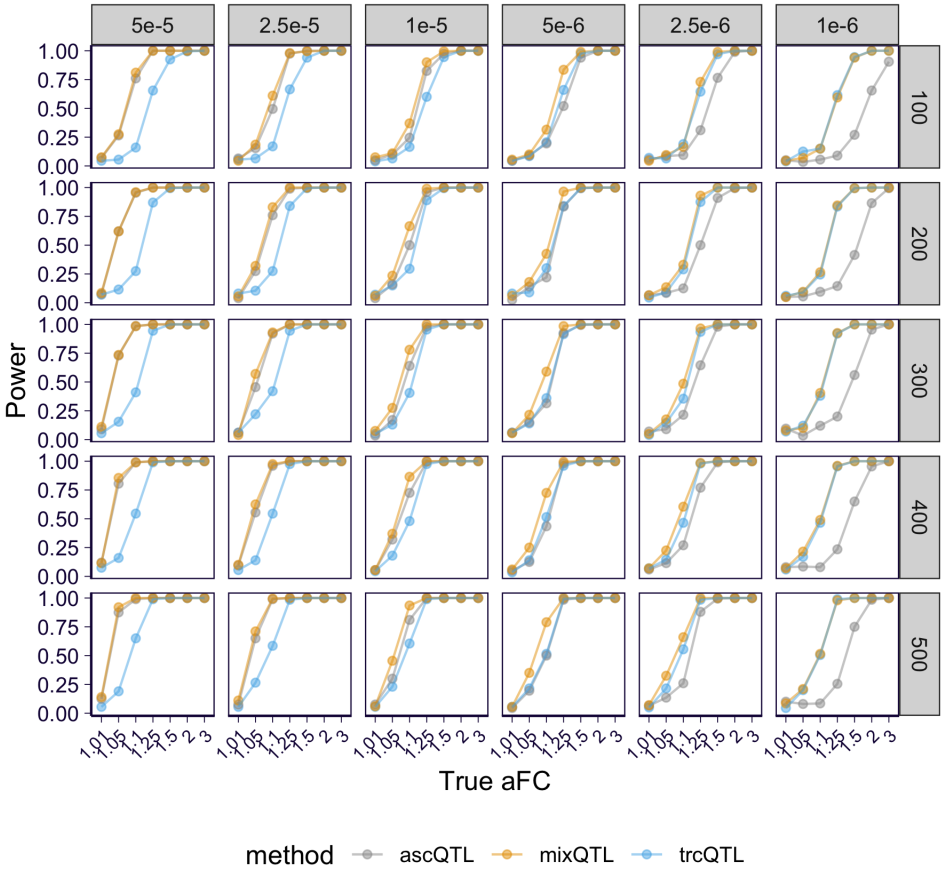


**Supplementary Figure 2. Power of mixQTL, ascQTL, and trcQTL on the full grid of simulations.** Each panel shows results on data simulated under a pair of $\theta$ (relative abundance in the simulation, by column) and sample size (by row). The power is calculated under significance level $\alpha = 0.05$.

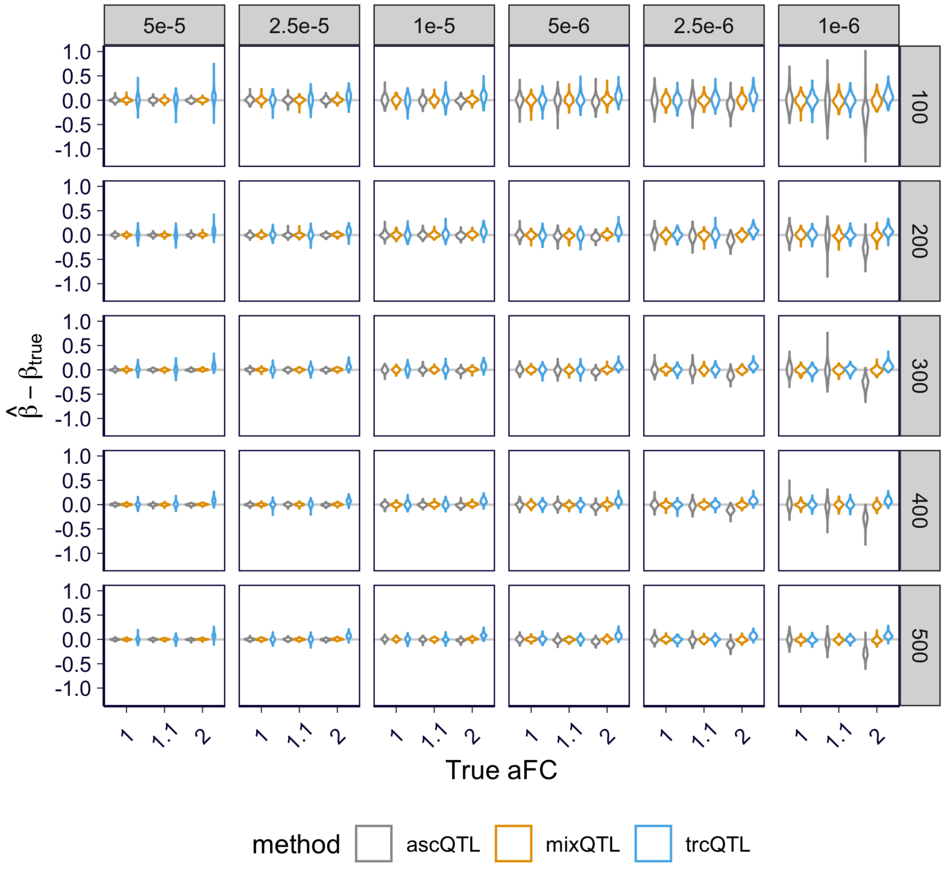


**Supplementary Figure 3. Difference between $\hat{\beta}$ and true $\beta$ of mixQTL, ascQTL, and trcQTL on the full grid of simulations.** Each panel shows results on data simulated under a pair of $\theta$ (relative abundance in the simulation, by column) and sample size (by row). The difference between the estimated log allelic fold change and the true log allelic fold change is shown on y-axis.

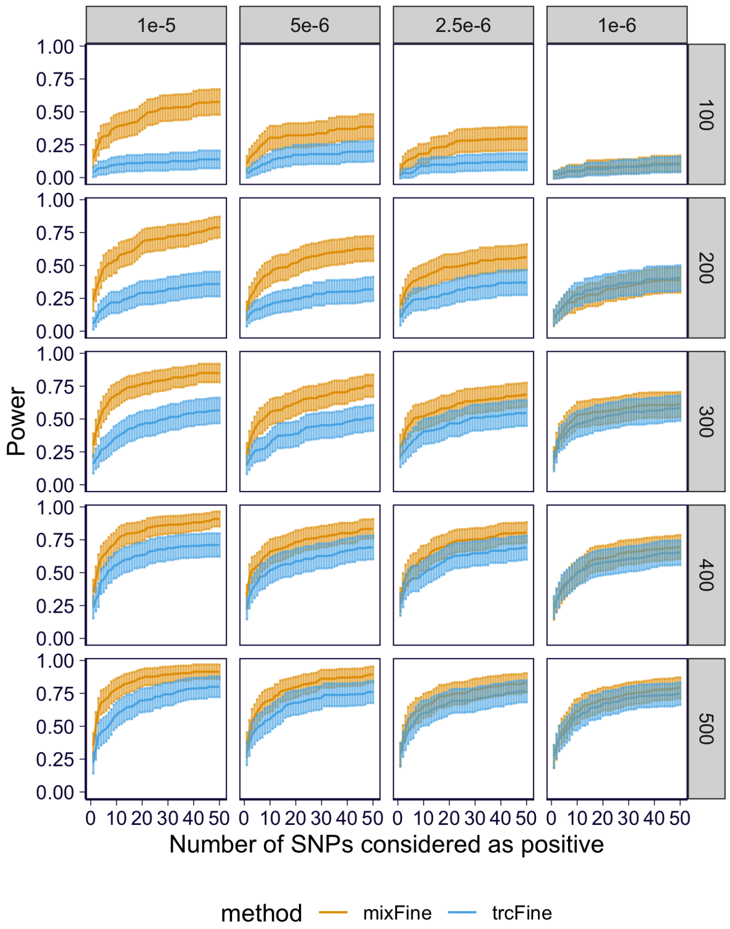


**Supplementary Figure 4. Power curves of mixFine and trcFine on the full grid of simulations.** Each panel shows results on data simulated under a pair of $\theta$ (relative abundance in the simulation, by column) and sample size (by row). In each panel, the curve is based on 200 simulation replicates with 100 simulations having signals and 100 simulations being drawn from the null. The solid curves indicate the mean power (recall rate) among 100 simulation replicates and the error bars indicate the 95% confidence interval.

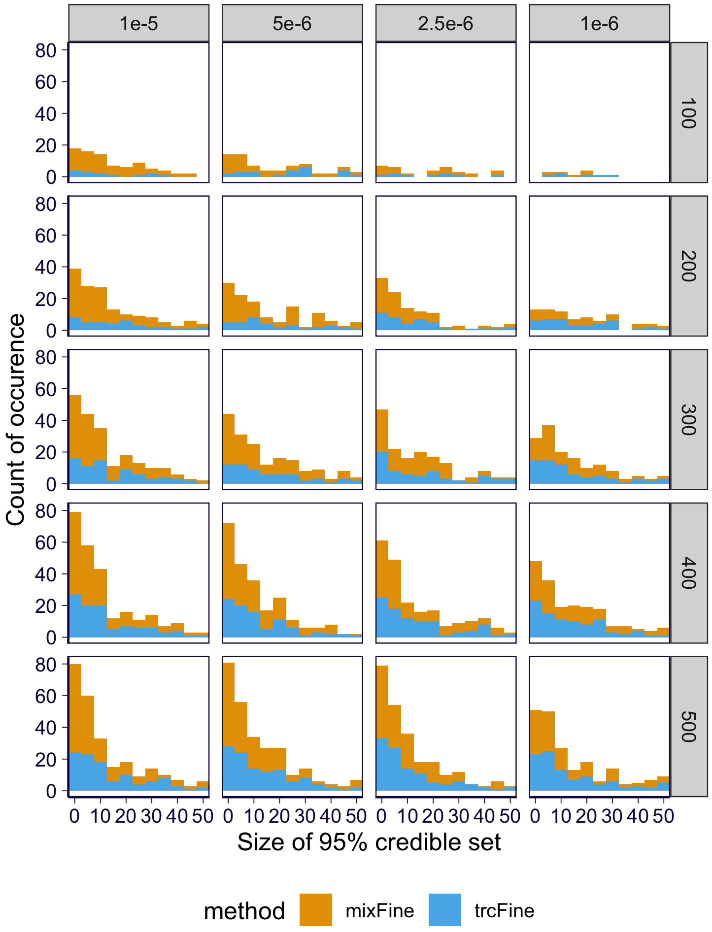


**Supplementary Figure 5. Distribution of the positive 95% CS's which contain causal variants in mixFine and trcFine on the full grid of simulations.** Each panel shows results on data simulated under a pair of $\theta$ (relative abundance in the simulation, by column) and sample size (by row).

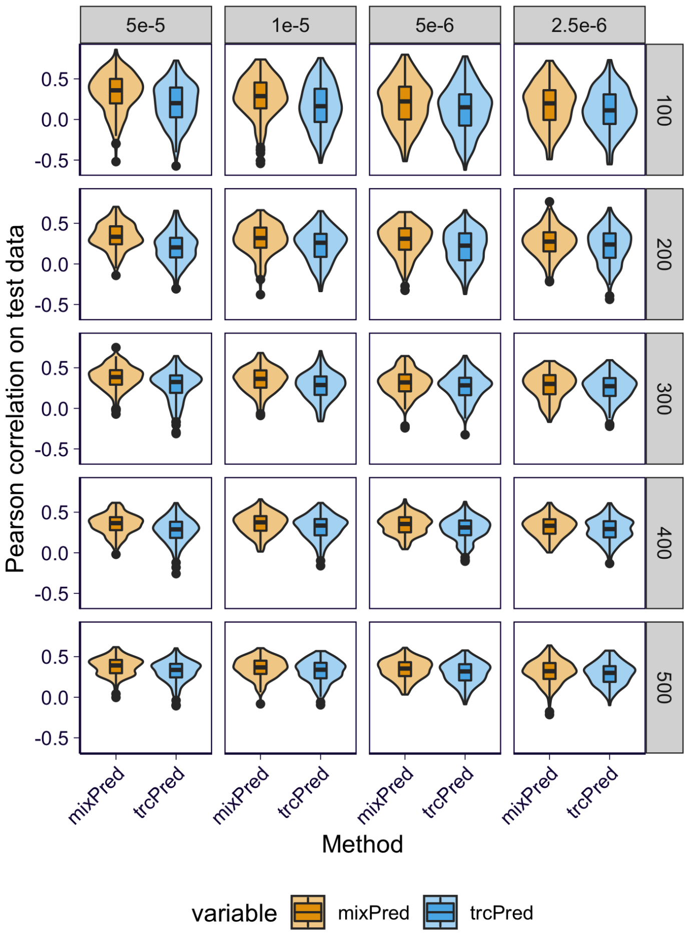


**Supplementary Figure 6. Distribution of Pearson correlations between predicted and observed expression level (in the scale $\log(Y_i^{\text{total}}/L_i)$) for mixPred and trcPred on the full grid of simulations.** Correlation is calculated on held-out test data. Each panel shows results on data simulated under a pair of $\theta$ (relative abundance in the simulation, by column) and sample size (by row). For each panel, the plot is based on 200 simulation replicates. In the boxplots, the lower and upper hinges show the first and third quartiles and the middle line shows the median. The whiskers extend from the hinge to the maximum and minimum at most 1.5x the inter-quartile range. All data points beyond the end of the whiskers are plotted individually.

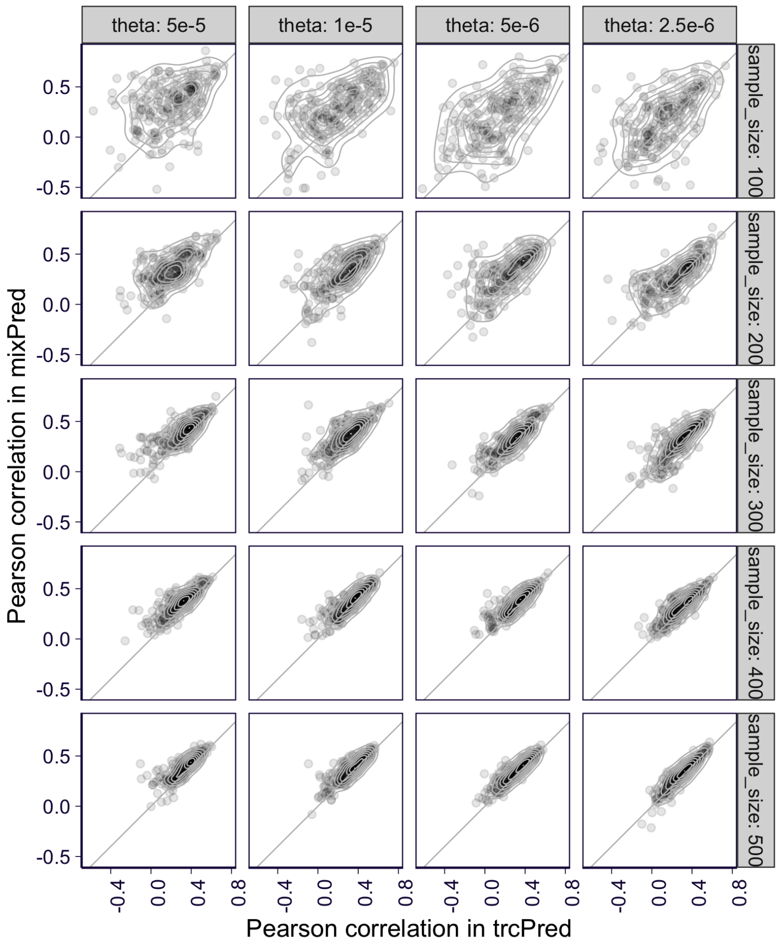


**Supplementary Figure 7. Pairwise comparison of prediction performance of mixPred and trcPred on the full grid of simulations.** Correlation of predicted versus observed expression level (in the scale $\log(Y_i^{\text{total}}/L_i)$) is calculated on held-out test data. The prediction performance of mixPred (y-axis) is plotted against the prediction performance of trcPred (x-axis) for each split. Each panel shows results on data simulated under a pair of $\theta$ (relative abundance in the simulation, by column) and sample size (by row).

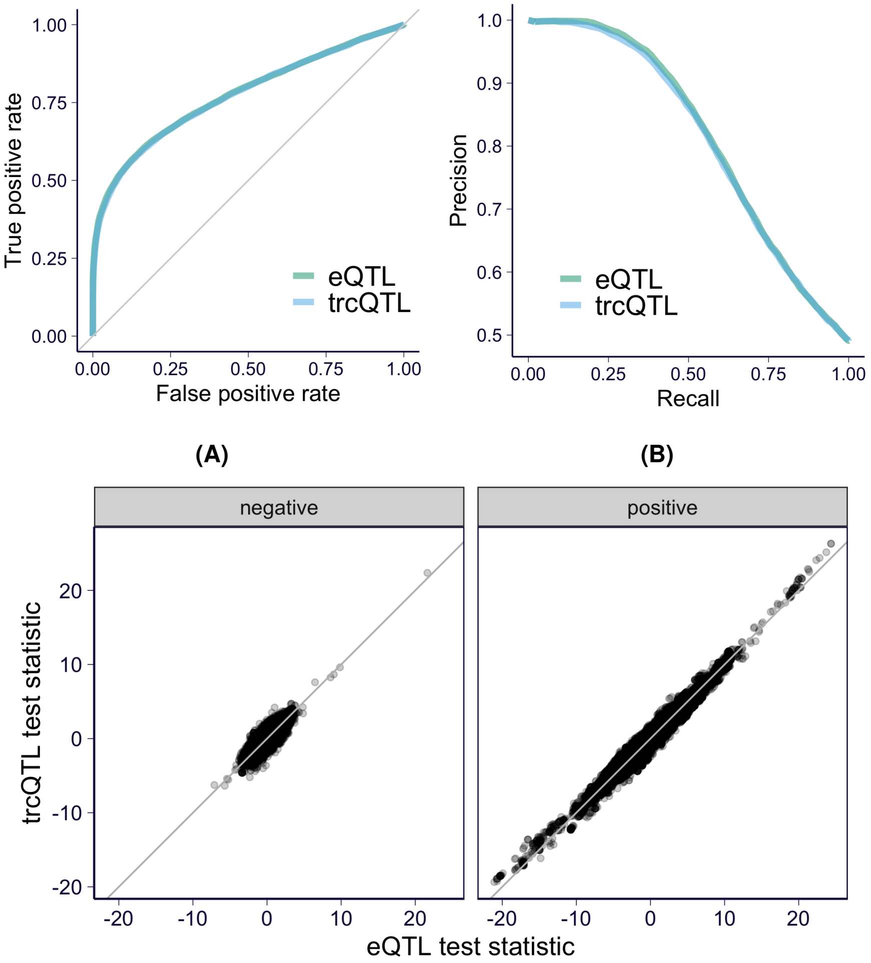


(C)

**Supplementary Figure 8. The performance of trcQTL and the standard eQTL approach on genes with low total read counts.** Genes with low total counts are defined as having no more than 50 total read counts in any one sample. In GTEx v8 whole blood samples, we extracted 912 genes with low total counts and calculated trcQTL estimates for variants in the corresponding cis-windows. To compare the power of trcQTL and eQTL, we used the 85,129 variant/gene pairs with FDR $< 0.05$ in eQTLGen as a "ground truth" set. We also randomly selected 88,242 variant/gene pairs from the pairs with p-value $> 0.5$ in eQTLGen as a negative set. **(A,B)** ROC and PR curves for trcQTL and the standard eQTL method. **(C)** Test statistics for the standard eQTL method (x-axis) and trcQTL (y-axis). The variant/gene pairs in the eQTLGen negative set are shown in the left panel, and pairs in the "ground truth" set in the right panel.

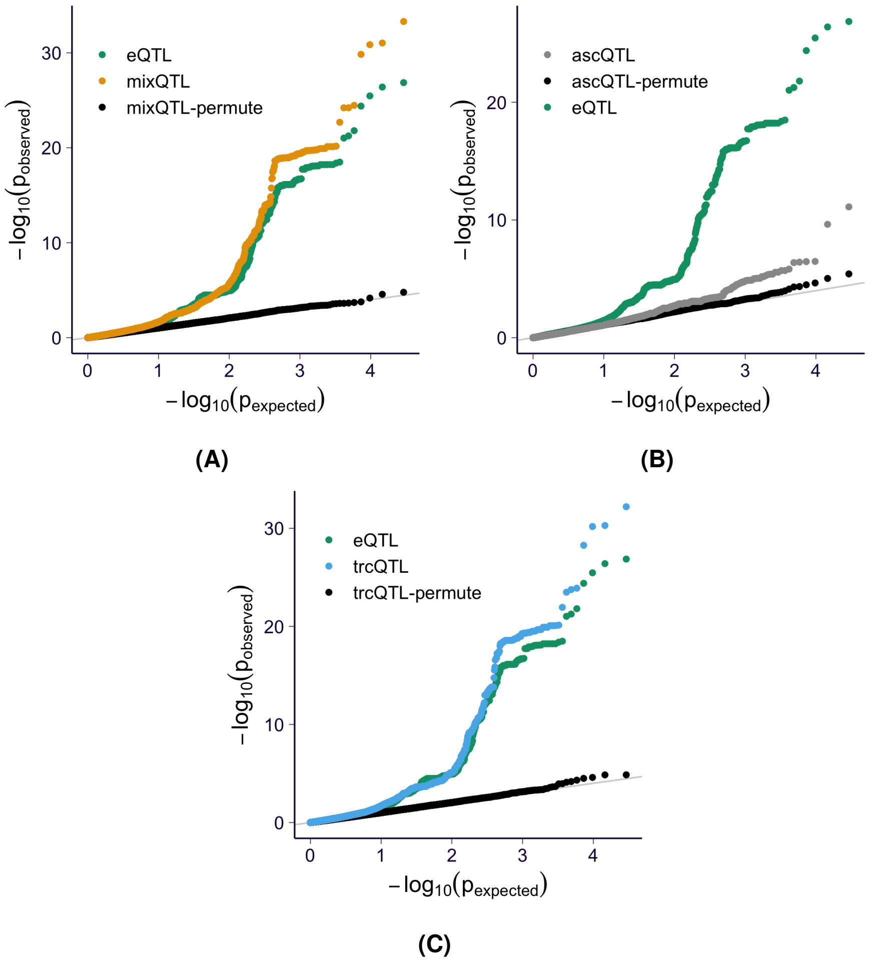


**Supplementary Figure 9. QQ-plot of nominal p-values from ascQTL and trcQTL on four randomly selected genes in GTEx v8 whole blood RNA-seq.** The nominal p-values of trcQTL and ascQTL are compared against the standard eQTL method for four randomly selected genes ENSG00000000457, ENSG00000001461, ENSG00000002834, and ENSG00000277734. The results of ascQTL and trcQTL on permuted genotypes are shown in black. **(A)** Results from mixQTL. **(B)** Results from ascQTL. **(C)** Results from trcQTL.

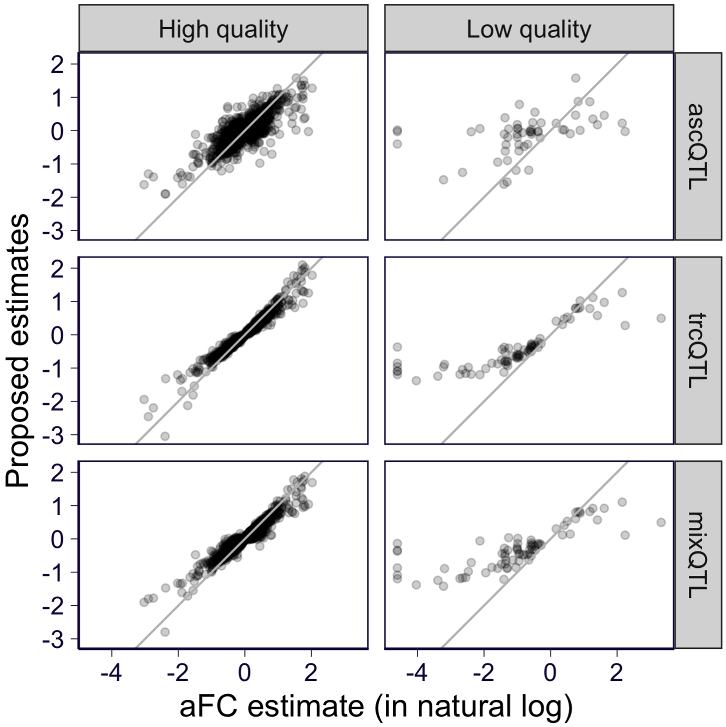


**Supplementary Figure 10. Comparison of aFC estimates from GTEx v8 and the estimated allelic fold change of ascQTL, trcQTL, and mixQTL.** The estimates of the top variants in the eGenes of GTEx v8 whole blood are shown (based on eQTL results). On the x-axis, the aFC estimate reported by GTEx v8 is shown (the reported value is in $\log_2$ and, for visualization, we rescale it to natural log scale by multiplying the value with $\log(2)$). On the y-axis, the estimated allelic fold changes (in natural log scale) of ascQTL, trcQTL, and mixQTL are shown. The variant/gene pairs are stratified on the basis of the quality of aFC estimate, which is defined as 'high quality' if the 95% confidence interval of $\log_2$ aFC is smaller than 1 and the low and high boundaries of the 95% confidence interval are not more extreme than $-\log_2(50)$ and $\log_2(50)$, and as 'low quality' otherwise.

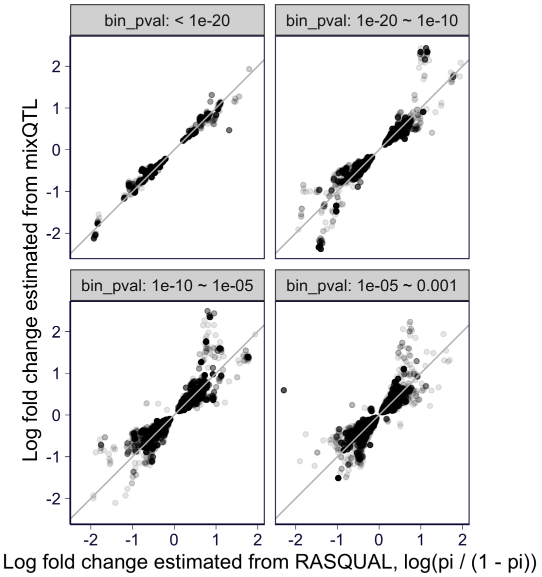


**(A)**

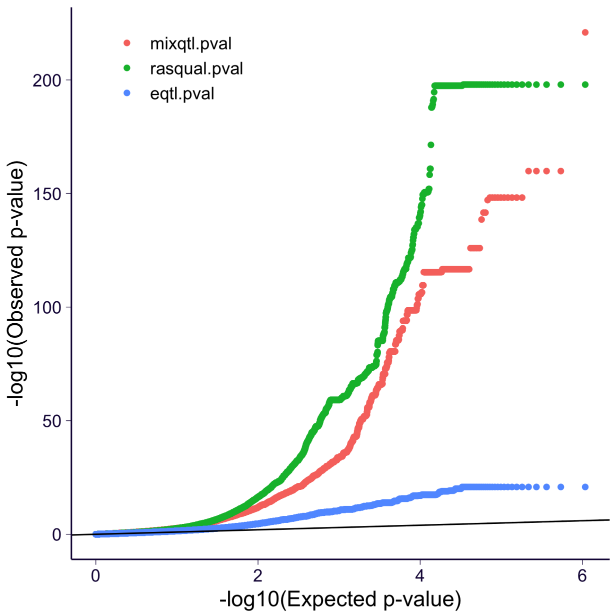


**(B)**

**Supplementary Figure 11. The performance of RASQUAL in GTEx v8 kidney cortex RNA-seq.** Here we show the results on kidney cortex for the gene/variants pairs within ±50kbp of the transcription start side. We tested the gene with enough allele-specific counts. Specifically, we include genes that have more than 100 reads (total count) in at least 80% of the samples and 50 allele-specific reads per haplotypes (both haplotypes should meet the criterion) in at least 15 samples. With these criteria, 4,596 genes are included. **(A)** The estimated effect sizes (in terms of log fold change) of both RASQUAL (on x-axis) and mixQTL (on y-axis). For RASQUAL, the log fold change is calculated from RASQUAL parameter $\pi$ using the relation that log fold change = $\log \frac{\pi}{1-\pi}$. The plot includes variant/gene pairs that both RASQUAL and mixQTL p-values pass some cutoffs (as stratified in the different panels). The concordance is similar across different minor allele frequencies. **(B)** QQ-plot of all the variant/gene pairs being tested.

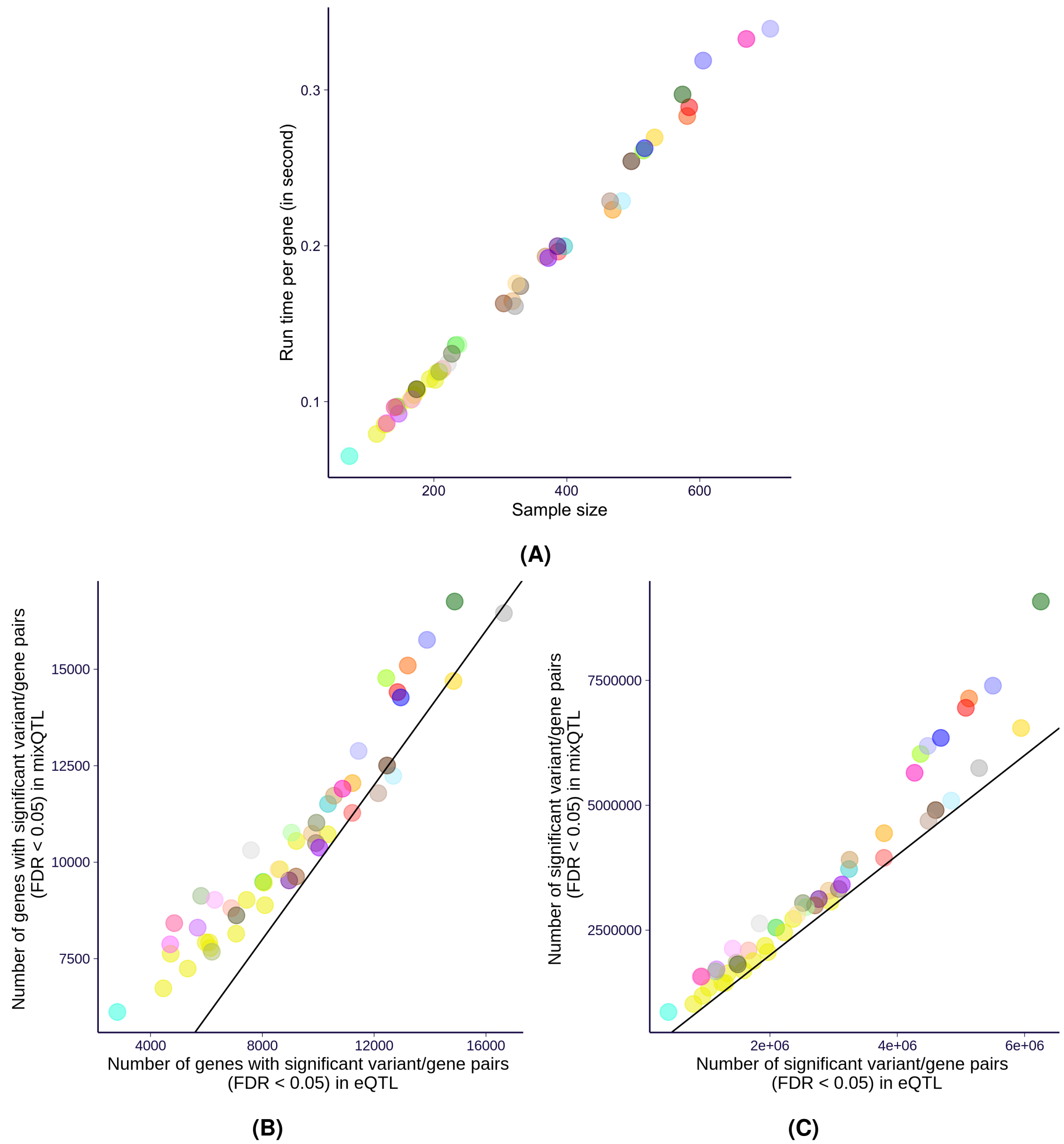


**Supplementary Figure 12. Running mixQTL on the full GTEx v8 data. (A)** The average runtime (clock time under 8 CPU cores) per gene is shown for each of the 49 tissues (y-axis) against the corresponding sample size (x-axis). **(B)** The number of genes that have at least one variant passing FDR control at 0.05 is shown for both mixQTL (y-axis) and the standard approach (x-axis). In the GTEx v8 main eQTL analysis, “eGene” was defined based on permutation-based analysis. Here we do not perform permutation so, to avoid confusion, we do not use the term “eGene”. **(C)** The number of variant/gene pairs that pass FDR control at 0.05 is shown for both mixQTL (y-axis) and the standard approach (x-axis).

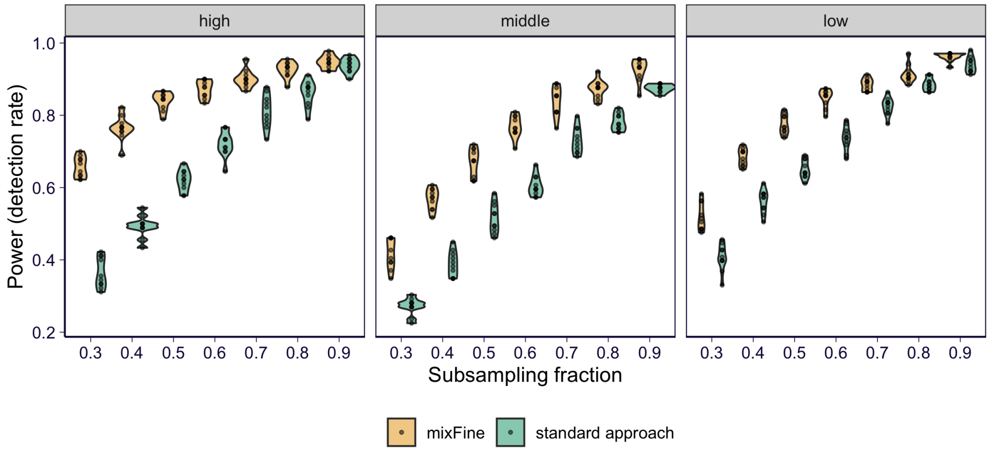


**Supplementary Figure 13. The performance of mixFine on GTEx v8 whole blood RNA-seq stratified by expression level.** At each subsampling level (x-axis), the fraction of "consensus SNPs" being detected is shown on the y-axis. Each panel shows the results of genes stratified by expression level tertiles in which the fraction is calculated within each expression level category. Among the 272 "consensus SNPs", 90 belong to "high" expression level, 89 belong to "middle" level, and 103 belong to "low" level. The subsampling analysis are repeated 10 times. The plot of each panel shows the results of all the ten replications.

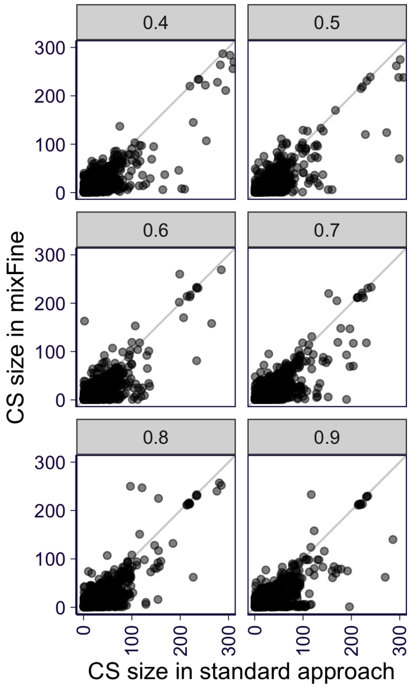


**Supplementary Figure 14. The performance of mixFine on GTEx v8 whole blood RNA-seq on pinpointing the "top" SNPs.** At each subsampling level (shown in each panel), we compare mixFine (y-axis) and the standard method (x-axis) on the size of 95% CS's which are paired by sharing the same "top SNP".

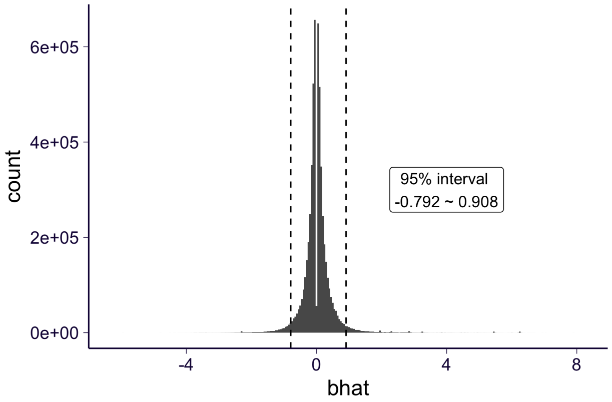


**Supplementary Figure 15. The estimated cis-eQTL effect size in GTEx v8 whole blood.** We examined the estimated effect sizes by mixQTL (in GTEx v8 whole blood) among the variant/gene pairs with FDR < 0.05. The 95% intervals (2.5% quantile to 97.5% quantile) of the estimated effect size are shown. Note that the estimated effect size (x-axis) is defined as allelic fold change in log-scale.

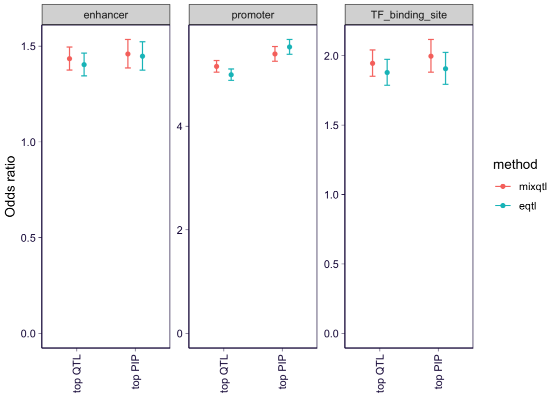


**(A)**

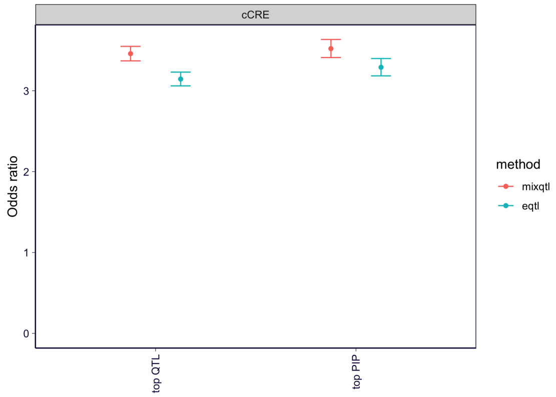


**(B)**

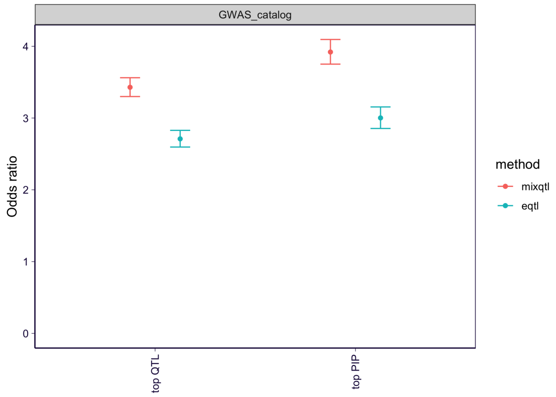


**(C)**

**Supplementary Figure 16. Enrichment in functional annotation for GTEx v8 tissues.** The enrichment is measured by odds ratio which is based on the 2-by-2 table indicating if the variant is in the annotation and if the variant is the top signal within a gene according to mixQTL or mixFine. The result is calculated by aggregating across 26 GTEx v8 tissues which have sample size $< 260$ and 221,920,351 tissue-gene-variant tuples are considered in total. The error bar indicates the 95% confidence interval. The enrichment is examined among all genes with enough allele-specific counts. **(A)** The enrichment of top mixQTL and mixFine signal in regulatory element annotations curated by GTEx v8 paper [1]. **(B)** The enrichment of top mixQTL and mixFine signal in candidate cis-regulatory elements (cCREs) [2] where only 10 of the 26 tissues are included due to the lack of matched tissue in cCRE data. In total, 85,170,905 tissue-gene-variant tuples are considered. **(C)** The enrichment of top mixQTL and mixFine signal in GWAS catalog variants.

| nfold | sample_size | pairwise_diff | diff_ci95_low | diff_ci95_high | pval | median_mixpred | median_standard |
|---|---|---|---|---|---|---|---|
| 10 | 67 | 0.052 | 0.047 | 0.057 | 1.318e-72 | 0.175 | 0.070 |
| 9 | 75 | 0.050 | 0.044 | 0.055 | 4.828e-58 | 0.185 | 0.079 |
| 8 | 84 | 0.049 | 0.044 | 0.054 | 4.569e-63 | 0.198 | 0.100 |
| 7 | 96 | 0.047 | 0.042 | 0.053 | 1.350e-57 | 0.214 | 0.119 |
| 6 | 112 | 0.043 | 0.038 | 0.049 | 4.884e-53 | 0.228 | 0.152 |
| 5 | 134 | 0.036 | 0.031 | 0.041 | 1.483e-39 | 0.241 | 0.195 |
| 4 | 168 | 0.028 | 0.023 | 0.032 | 1.791e-27 | 0.251 | 0.219 |
| 3 | 224 | 0.017 | 0.012 | 0.021 | 2.535e-12 | 0.266 | 0.254 |
| 2 | 335 | 0.007 | 0.002 | 0.011 | 3.354e-03 | 0.292 | 0.287 |

**Supplementary Table 1. The pairwise comparison of the prediction performance between mixPred and the standard approach based on the cross-validated evaluation.** The GTEx v8 whole blood data (sample size = 670) is split into $k$ folds. To evaluate the prediction performance, we train a model using one fold of the data and measure the performance on the held-out $(k-1)$ folds. This routine is applied to 1,000 genes and, for each gene, it is repeatedly $k$ times going through each of the $k$ folds. The prediction performance is measured by Pearson correlation. The **nfold** columns shows the number of folds, and, correspondingly, the **sample_size** column shows the number of samples used for training. The **pairwise_diff** column shows the average pairwise difference (mixPred vs. the standard approach) of the prediction performance among all folds and genes. And the **diff_ci95_low** and **diff_ci95_high** columns show the lower and upper bounds of the 95% confidence interval of the pairwise difference. The **pval** shows the p-value of the pairwise difference under paired t test (two-sided). The median of the prediction performance among all folds and genes are shown in the **median_mixpred** and **median_standard** columns for mixPred and the standard approach respectively.

# Supplementary Notes

# 1 Statistical model for read count

Here we introduce the statistical model of read count in this paper. For completeness, we opt for keeping some text that overlaps with main text. Recall that $i$ indexes individual and $h$ indexes haplotypes. $X_i^h$ is the phased genotype of the corresponding individual $i$ haplotype $h$. $Y_i^{\text{total}}$ is the total read count within the gene body and $L_i$ is the library size. $Y_i^{(h)\text{obs}}$ is the allele-specific read count of the corresponding haplotype transcript $h$ and $Y_i^h$ is the actual (though unobserved) read count of the haplotype transcript $h$. $\alpha_i$ is the expected fraction of allele-specific reads in individual $i$. Additionally, the cis-genetic effect of a single SNP on haplotype $h$ is represented as $g(\beta, X_i^h)$ where

$$g(\beta, X_i^h) = \begin{cases} 1 & \text{, if } X_i^h = 0 \\ e^{\beta} & \text{, if } X_i^h = 1 \end{cases} \tag{1}$$

$$= e^{X_i^h \beta} \tag{2}$$

We assume multiplicative effect when there are multiple causal SNPs. And the effect of multiple SNPs $j = 1, \cdots, p$ is

$$\prod_{j=1}^{p} g(\beta_j, X_{ij}^h) = e^{\sum_j X_{ij}^h \beta_j} \tag{3}$$

$$= e^{\mathbf{X}_i^h \boldsymbol{\beta}} \tag{4}$$

$$:= g(\boldsymbol{\beta}, \mathbf{X}_i^h) \tag{5}$$

### 1.1 Overview

We model haplotypic count $Y_i^h$ as lognormal distribution as follow.

$$\log Y_i^h \sim N(\log(L_i \theta_i^h), \tau_i^h) \tag{6}$$
$$\theta_i^h = \theta_{0,i} \times g(\beta, \mathbf{X}_i^h), \tag{7}$$

$\theta_{0,i}$ is the baseline abundance of haplotype transcript without considering genetic effect (*i.e.* it represents the abundance when the affecting SNP is reference allele).

In practice, we do not observe $Y_i^h$ but allele-specific read count $Y_i^{(h)\text{obs}}$. So, we further assume that the baseline abundance of corresponding allele-specific reads are $\theta_{0,i}^{(1)} = \theta_{0,i}^{(2)} = \alpha_i \theta_{0,i}$. And by definition, total read count $Y_i^{\text{total}} = Y_i^1 + Y_i^2$. So, similar to Eq 6, 7, $Y_i^{(h)\text{obs}}$ and $Y_i^{\text{total}}$ follow

$$\log Y_i^{(h)\text{obs}} \sim N(\log(L_i \theta_i^{(h)}), \tau_i^{(h)}) \tag{8}$$
$$\log Y_i^{\text{total}} \sim N(\log(L_i \theta_i), \tau_i) \tag{9}$$
$$\theta_i^{(h)} = \alpha_i \theta_{0,i} \times g(\beta, \mathbf{X}_i^h) \tag{10}$$
$$\theta_i = \theta_{0,i} \times [g(\beta, \mathbf{X}_i^1) + g(\beta, \mathbf{X}_i^2)] \tag{11}$$

### 1.2 Parameterizing $\tau$ to weight total and AS count properly

Note that lognormal distribution has the following property.

$$\log X \sim N(\mu, \tau) \tag{12}$$
$$X \sim \text{lognormal}(\mu, \tau) \text{ , by definition of lognormal} \tag{13}$$
$$\text{E}(X) = e^{\mu + \frac{\tau}{2}} \tag{14}$$
$$\text{Var}(X) = (e^{\tau} - 1)(e^{2\mu + \tau}) \tag{15}$$

When modeling read count, given the mean, we would like the variance to scale linearly with the mean (as assumed in RASQUAL [3]). In other word, we want to ensure that $\text{Var}(X)/\text{E}(X)$, also known as over-dispersion parameter, is roughly a constant. From Eq 14, 15 we have $\text{Var}(X) = (e^{\tau} - 1)\text{E}(X)^2$. For count data, since $\tau$ is capturing the variation of count in log-scale, $\tau$ is typically close to 0. So $e^{\tau} - 1 \approx \tau$ and $\text{Var}(X) \approx \tau \text{E}(X)^2$. This result suggests that to ensure $\text{Var}(X)/\text{E}(X) = \text{constant}$, $\tau$ should be approximately proportional to $1/\text{E}(X)$. So, for the distribution of $Y \sim \text{lognormal}(\log(L\theta), \tau)$, we impose the constraint on $\tau$ such that $\tau \approx \sigma^2/\text{E}(Y)$. In practice, $\text{E}(Y)$ is unknown so that we plug-in $Y$ in replace of $\text{E}(Y)$.

## 2 Single-SNP model

On the basis of the model described in Supplementary Notes 1.1, we propose the single-SNP model where we focus on one ”test SNP” $X_i^h$ instead of the whole phased haplotype $\mathbf{X}_i^h$. Hence, the cis-genetic effect of interest is $g(\beta, X_i^h)$.

### 2.1 From likelihood to linear mixed model

Here, we model cis-genetic effect of test SNP as allelic fold change (aFC) [4]. So $\beta$ is log-scale aFC in $g(\beta, X_i^{(h)}) = e^{X_i^{(h)}\beta}$. From Eq 8, 10, we have (for $h = 1, 2$)

$$\log Y_i^{(h)\text{obs}} = \log L_i + \log \theta_i^{(h)} + \epsilon_i^{(h)} \tag{16}$$
$$= \log L_i + \log \alpha_i + \log \theta_i^h + \epsilon_i^{(h)} \tag{17}$$
$$= \log L_i + \log \alpha_i + \log \theta_{0,i} + \log(e^{X_i^h \beta}) + \epsilon_i^{(h)} \tag{18}$$
$$= \log L_i + \log \alpha_i + \log \theta_{0,i} + X_i^h \beta + \epsilon_i^{(h)} \tag{19}$$
$$\epsilon_i^{(h)} \sim N(0, \frac{\sigma^2}{Y_i^{(h)}}), \tag{20}$$

where the error term scaling in Eq 20 follows from the discussion in Supplementary Notes 1.2. To further simplify the term $\log \theta_{0,i}$, as the variation of baseline abundance among individuals, we assume $\log \theta_{0,i} \sim N(\mu_0, \sigma_0^2)$. So that Eq 19, 20 can be further written as

$$\log Y_i^{(h)\text{obs}} = \mu_0 + \log L_i + \log \alpha_i + z_i + X_i^h \beta + \epsilon_i^{(h)} \tag{21}$$
$$\epsilon_i^{(h)} \sim N(0, \frac{\sigma^2}{Y_i^{(h)\text{obs}}}),\ z_i \sim N(0, \sigma_0^2), \tag{22}$$

which is the approximated likelihood function for allele-specific counts $Y_i^{(1)\text{obs}}$ and $Y_i^{(2)\text{obs}}$. Such likelihood function is equivalent to linear mixed effects model.

Furthermore, we can linearize the likelihood of total read count $Y_i^{\text{total}}$ in similar fashion. From Eq 9, 11 , we have

$$\log Y_i^{\text{total}} = \mu_0 + \log L_i + z_i + \log(\theta_i^1 + \theta_i^2) + \epsilon_i \tag{23}$$
$$= \mu_0 + \log L_i + z_i + \log(e^{X_i^1 \beta} + e^{X_i^2 \beta}) + \epsilon_i \tag{24}$$
$$\epsilon_i \sim N(0, \frac{\sigma^2}{Y_i^{\text{total}}}),\ z_i \sim N(0, \sigma_0^2) \tag{25}$$

Here we linearize $\log(e^{X_i^1 \beta} + e^{X_i^2 \beta})$ under the weak-effect assumption as follow

$$\log(e^{X_i^1 \beta} + e^{X_i^2 \beta}) = \log[(X_i^1 e^\beta + 1 - X_i^1) + (X_i^2 e^\beta + 1 - X_i^2)] \tag{26}$$
$$= \log(2 + X_i e^\beta - X_i) \quad \text{, let } X_i = X_i^1 + X_i^2 \tag{27}$$
$$= \log[2 + X_i(e^\beta - 1)] \tag{28}$$
$$= \log 2 + \frac{1}{2}(e^\beta - 1)X_i + o(X_i(e^\beta - 1)) \tag{29}$$
$$\approx \log 2 + \frac{1}{2} X_i \beta \quad \text{, when } \beta \text{ is close to 0} \tag{30}$$

So that Eq 24 can be approximated as

$$\log \frac{Y_i^{\text{total}}}{2} \approx \mu_0 + \log L_i + z_i + \frac{X_i^1 + X_i^2}{2} \beta + \epsilon_i \tag{31}$$

In summary, combining Eq 21 ,25, 22, 31, we have a linear mixed effects model unifying total and allele-specific read counts after linearization along with other approximations. And it also serves as an approximated likelihood for total and allele-specific reads, in which we can see that these read counts are not independent since they share the same random effect $z_i$.

## 2.2 Simplifying the model

Note that $\alpha_i$ is not observed so that we are unable to solve the model proposed in Supplementary Notes 2.1 in a computationally efficient manner. Here we address this problem by re-parameterizing the model. In principle, conditioning on genetic effect $\beta$, the ratio of allele-specific reads should be independent to the observations on the total read counts. This intuition motivates us to model the ratio of $Y_i^{(1)\text{obs}}$ and $Y_i^{(2)\text{obs}}$ rather than each of them separately. Mathematically, we subtract $\log Y_i^{(2)\text{obs}}$ from $\log Y_i^{(1)\text{obs}}$, which gives

$$\log \frac{Y_i^{(1)\text{obs}}}{Y_i^{(2)\text{obs}}} = (X_i^1 - X_i^2)\beta + \epsilon_i^{\text{asc}} \tag{32}$$

$$\epsilon_i^{\text{asc}} \sim N(0, \sigma^2(\frac{1}{Y_i^{(1)\text{obs}}} + \frac{1}{Y_i^{(2)\text{obs}}})), \tag{33}$$

where both $z_i$ and $\alpha_i$ cancel out. This result naturally shows that the likelihood function of $Y_i^{\text{total}}$ and $\frac{Y_i^{(1)\text{obs}}}{Y_i^{(2)\text{obs}}}$ takes the form:

$$\mathcal{L}(\mathbf{Y}^{\text{total}}, \frac{\mathbf{Y}^{(1)\text{obs}}}{\mathbf{Y}^{(2)\text{obs}}}; \mu_0, \sigma_0^2, \sigma^2, \beta) = \prod_i \Pr(Y_i^{\text{total}} | \mu_0, \sigma_0^2, \sigma^2, \beta) \Pr(\frac{Y_i^{(1)\text{obs}}}{Y_i^{(2)\text{obs}}} | \sigma^2, \beta) \tag{34}$$

$$= \underbrace{\prod_i \Pr(Y_i^{\text{total}} | \mu_0, \sigma_0^2, \sigma^2, \beta)}_{\text{total read count likelihood}} \quad \underbrace{\prod_i \Pr(\frac{Y_i^{(1)\text{obs}}}{Y_i^{(2)\text{obs}}} | \sigma^2, \beta)}_{\text{allele-specific read count likelihood}} \tag{35}$$

$$:= \mathcal{L}^{\text{trc}}(\mathbf{Y}^{\text{total}}) \times \mathcal{L}^{\text{asc}}(\frac{\mathbf{Y}^{(1)\text{obs}}}{\mathbf{Y}^{(2)\text{obs}}}) \tag{36}$$

With the simplification shown in Eq 32, the model used for inference can be summarized as follow

$$\log \frac{Y_i^{\text{total}}}{2L_i} = \mu_0 + z_i + \frac{X_i^1 + X_i^2}{2}\beta \quad + \epsilon_i^{\text{trc}} \tag{37}$$

$$\log \frac{Y_i^{(1)\text{obs}}}{Y_i^{(2)\text{obs}}} = \qquad (X_i^1 - X_i^2)\beta + \epsilon_i^{\text{asc}} \tag{38}$$

$$z_i \sim N(0, \sigma_0^2), \;\; \epsilon_i^{\text{trc}} \sim N(0, \frac{\sigma^2}{Y_i^{\text{total}}}), \;\; \epsilon_i^{\text{asc}} \sim N(0, \frac{\sigma^2 Y_i^{(1)\text{obs}} + Y_i^{(2)\text{obs}}}{Y_i^{(1)\text{obs}} Y_i^{(2)\text{obs}}}) \tag{39}$$

# 3 Generalizing to multi-SNP model

The linearized model described in Eq 37, 38, 39 is easily extensible to multi-SNP scenario since we assume multiplicative genetic effect, as described in Supplementary Notes 5. To see the extension, all we need to examine is how $\log \theta_i^h$ and $\log(\theta_i^1 + \theta_i^2)$ as compared to the single SNP case since the rest of the terms stay

the same.

$$\log \theta_i^h = \log \theta_{0,i} + \log g(\beta, \mathbf{X}_i^h) \tag{40}$$
$$= \log \theta_{0,i} + \log e^{\mathbf{X}_i^h \beta} \tag{41}$$
$$= \log \theta_{0,i} + \mathbf{X}_i^h \beta \tag{42}$$
$$\log(\theta_i^1 + \theta_i^2) = \log \theta_{0,i} + \log\{\prod_j [1 + (e^{\beta_j} - 1)X_{ij}^1] + \prod_j [1 + (e^{\beta_j} - 1)X_{ij}^2]\}, \tag{43}$$
$$\text{similar to Eq 26} \tag{44}$$
$$\approx \log \theta_{0,i} + \log[1 + \sum_j (e^{\beta_j} - 1)X_{ij}^1 + 1 + \sum_j (e^{\beta_j} - 1)X_{ij}^2], \tag{45}$$
$$\text{high orders term like } (e^{\beta_j} - 1)X_{ij}^1(e^{\beta_{j'}} - 1)X_{ij'}^1 \text{ are ignored} \tag{46}$$
$$= \log \theta_{0,i} + \log(2 + \sum_j (e^{\beta_j} - 1)X_{ij}) \ , X_{ij} := X_{ij}^1 + X_{ij}^2 \tag{47}$$
$$\approx \log \theta_{0,i} + \log 2 + \frac{1}{2}\mathbf{X}_i \beta \ \text{, follows similarly as Eq 29, 30} \tag{48}$$

So, we can simply plug-in the multi-SNP version of $\log \theta_i^h$ and $\log(\theta_i^1 + \theta_i^2)$ to Eq 17 and 23 respectively and the similar conclusion follows with $\mathbf{X}$ and $\beta$ in replace of $X$ and $\beta$.

# 4 QTL mapping procedure

In the following, we describe the mixQTL procedure to map cis-eQTLs under the model proposed in Eq 37, 38, 39.

## 4.1 Converting the problems into two linear regressions

Instead of solving the proposed mixed effects model using numerical solver, we propose a meta-analysis procedure. In this procedure, we solve Eq 37 and 38 separately and meta-analyze the estimates afterwards.

Here we recognize that $\epsilon_i^{\text{trc}}$ in Eq 37 is approximate independent to $\epsilon_i^{\text{asc}}$ in Eq 38. The reason is that, under the model assumption, the read counts from each of the two haplotypes are independent (conditioning on $z_i$ and library size), which is also true in log-scale, *i.e.* $\epsilon^{(1)} \perp\!\!\!\perp \epsilon^{(2)}$. So, $\epsilon^{(1)} + \epsilon^{(2)} \perp\!\!\!\perp \epsilon^{(1)} - \epsilon^{(2)}$, which means that the sum of logarithm of the haplotypic counts, $\log Y_i^1 + \log Y_i^2$, is independent to the haplotypic imbalance signal, $\log Y_i^1 / Y_i^2$. Furthermore, under the weak effect size assumption, $\log Y_i^1 + \log Y_i^2 \approx \log Y_i^{\text{total}}$ so that $\epsilon_i^{\text{trc}}$ is approximately independent to $\epsilon_i^{\text{asc}}$. Besides, $z_i$ represents baseline abundance, which is independent of the multiplicative errors $\epsilon_i^{\text{trc}}$ and $\epsilon_i^{\text{asc}}$. So, we can further simplify Eq 37 by merging the noise term $\epsilon_i^{\text{trc}}$ and $z_i$ as a new term $\widetilde{z}_i$. Such simplification results in the following linear model

$$Y_i^{\text{trc}} = \mu_0 + X_i^{\text{trc}} \beta^{\text{trc}} + \widetilde{z}_i, \ \widetilde{z}_i \sim N(0, \widetilde{\sigma}_0^2) \ , \tag{49}$$

where $X^{\text{trc}} := \frac{X^1 + X^2}{2}$, $Y^{\text{trc}} = \log \frac{Y_i^{\text{total}}}{2L_i}$. Eq 49 itself can be used for QTL mapping and we call this approach trcQTL in the paper.

For solving Eq 38, notice that it is weighted simple linear regression with the form

$$Y_i^{\text{asc}} = X_i^{\text{asc}} \beta^{\text{asc}} + \epsilon_i^{\text{asc}}, \ \epsilon_i^{\text{asc}} \sim N(0, \sigma^2 / w_i) \ , \tag{50}$$

where $Y_i^{\text{asc}} = \log \frac{Y_i^{(1)\text{obs}}}{Y_i^{(2)\text{obs}}}$, $X_i^{\text{asc}} = X_i^1 - X_i^2$, $w_i = \frac{Y_i^{(1)\text{obs}} Y_i^{(2)\text{obs}}}{Y_i^{(1)\text{obs}} + Y_i^{(2)\text{obs}}}$. We call QTL mapped by Eq 50 ascQTL.

Note that we can combine Eq 49 and 50 and solve them jointly in close form. But here we still prefer meta-analysis for two reasons: 1) it allows combining summary statistics across studies; and 2) it allows the over-dispersion in total and allele-specific read counts to be different which is more realistic in practice since total and allele-specific read counts may go through different pre-processing steps.

Since the inference of linear regression has analytical solution which only involves $X^T X$ and $X^T Y$, we can solve it quickly and in a parallel way as proposed by Matrix eQTL [5]. We sketch the pseudocode on calculating trcQTL and ascQTL estimates in matrix form in Supplementary Notes 7.

## 4.2 Meta-analysis for QTL mapping

Once we obtain estimated $\widehat{\beta}^{\text{trc}}$ and $\widehat{\beta}^{\text{asc}}$, we can use these estimates to approximate $\mathcal{L}^{\text{trc}}$ and $\mathcal{L}^{\text{asc}}$ in Eq 36. Specifically, when sample size is large,

$$\mathcal{L}^{\text{trc}}(Y_i^{\text{total}}|\beta) \approx N(\beta; \widehat{\beta}^{\text{trc}}, \text{se}(\widehat{\beta}^{\text{trc}})) \tag{51}$$

$$\mathcal{L}^{\text{asc}}(\frac{Y_i^{(1)\text{obs}}}{Y_i^{(2)\text{obs}}}|\beta) \approx N(\beta; \widehat{\beta}^{\text{asc}}, \text{se}(\widehat{\beta}^{\text{asc}})) \tag{52}$$

So that the joint likelihood, as factorized in Eq 35, is simply $N(\beta; \widehat{\beta}^{\text{trc}}, \text{se}(\widehat{\beta}^{\text{trc}})) \times N(\beta; \widehat{\beta}^{\text{asc}}, \text{se}(\widehat{\beta}^{\text{asc}}))$. As shown previously [6], maximizing the approximate joint likelihood is equivalent to inverse-variance meta-analysis, which takes the form

$$\widehat{\beta}^{\text{mix}} = \frac{w^{\text{trc}}\widehat{\beta}^{\text{trc}} + w^{\text{asc}}\widehat{\beta}^{\text{asc}}}{w^{\text{trc}} + w^{\text{asc}}} \tag{53}$$

$$\text{se}(\widehat{\beta}^{\text{mix}}) = \sqrt{\frac{1}{w^{\text{trc}} + w^{\text{asc}}}}\ , \tag{54}$$

where $w^{\text{trc}} = 1/\text{se}(\widehat{\beta}^{\text{trc}})^2$ and $w^{\text{asc}} = 1/\text{se}(\widehat{\beta}^{\text{asc}})^2$.

# 5 Inference procedure for multi-SNP model

With the simplification made in Supplementary Notes 4.1, the multi-SNP model can be written as

$$Y_i^{\text{trc}} = \mu_0 + \mathbf{X}_i^{\text{trc}}\beta + \widetilde{z}_i \quad , \widetilde{z}_i \quad \sim N(0, \widetilde{\sigma}_0^2) \tag{55}$$

$$Y_i^{\text{asc}} = \quad \mathbf{X}_i^{\text{asc}}\beta + \epsilon_i^{\text{asc}}, \epsilon^{\text{asc}} \sim N(0, \sigma^2/w_i)\ . \tag{56}$$

## 5.1 Motivating two-step inference procedure

Here we focus on two inference problems under the multi-SNP model: 1) construct genetic predictor of expression; and 2) infer whether $\beta_k$ is non-zero, *i.e.* causal SNP. Problem 1) is prediction problem in machine learning context and in terms of building genetic predictor, elastic net has been used for this task as implemented in the PrediXcan method[7]. For problem 2), the inference problem is formulated into a Bayesian variable selection problem and efficient solvers such as susieR [8] and DAP-G [9] have been developed in the context of eQTL analysis.

However, the existing methods only use total read information (typically inverse normalized expression) and they assume the inversely normalized expression $Y$ and genotype vector $\mathbf{X}$ follow $Y \sim N(\mathbf{X}\beta, \nu)$. The modeling assumption is very close to Eq 55, 56 but it requires equal variance in error term and shared intercept across all observations. To apply the existing tools, we need to bypass the gap between our model and their modeling assumption. For this reason, we propose a two-step inference procedure to perform inference for multi-SNP model. In step 1, we infer $\widetilde{\sigma}_0^2$ and $\sigma^2$ and transform the data such that they

approximately follow $Y \sim N(\mathbf{X}\beta, \nu)$. And in step 2, we apply the transformed data to existing solvers for both prediction and fine-mapping problems.

### 5.2 Inferring $\widetilde{\sigma}_0^2$ and $\sigma^2$

To estimate $\widetilde{\sigma}_0^2$ and $\sigma^2$ from Eq 55 and Eq 56, we further assume that the genetic effects $\beta_1, \cdots, \beta_P$ (for all the SNPs within the cis-window) follow $\beta_p \sim_{iid} N(0, V_g)$. Or equivalently, we assume

$$Y^{\text{trc}} \sim N(\mu_0, \widetilde{\sigma}_0^2 I_N + V_g \mathbf{X}_i^{\text{trc}} (\mathbf{X}_i^{\text{trc}})') \tag{57}$$

$$Y^{\text{asc}} \sim N(0, \sigma^2 I_N + V_g \mathbf{X}_i^{\text{asc}} (\mathbf{X}_i^{\text{asc}})') \tag{58}$$

Under the mixed effect model Eq 57, we solve for $\widetilde{\sigma}_0^2$ using total read count data. And similarly, under the random effect model Eq 58, we solve for $\sigma^2$ using allele-specific count data. The actual computation is done using R package EMMA [10].

### 5.3 Data transformation and inference

Once we obtain $\widehat{\widetilde{\sigma}}_0^2$ and $\widehat{\sigma}^2$, we shift and re-scale the total and allelic imbalance observations by

$$\widetilde{Y}_i^{\text{trc}} = \frac{\text{center}(Y_i^{\text{trc}})}{\widehat{\widetilde{\sigma}}_0}, \ \widetilde{\mathbf{X}}_i^{\text{trc}} = \frac{\text{center}(\mathbf{X}_i^{\text{trc}})}{\widehat{\widetilde{\sigma}}_0} \tag{59}$$

$$\widetilde{Y}_i^{\text{asc}} = \frac{Y_i^{\text{asc}}}{\widehat{\sigma}}, \qquad \widetilde{\mathbf{X}}_i^{\text{asc}} = \frac{\mathbf{X}_i^{\text{asc}}}{\widehat{\sigma}}, \tag{60}$$

where the function center$(\cdot)$ centers the input by subtracting the population mean (mean across all samples). By centering $Y_i^{\text{trc}}$ and $\mathbf{X}_i^{\text{trc}}$, effectively, we account for the term $\mu_0$ in Eq 55, which has been deployed previously by [5, 11]. And the transformed data (on the left-hand side) is used for downstream analysis on performing prediction and fine-mapping.

Specifically, we concatenate $\widetilde{\mathbf{Y}}^{\text{trc}}$ and $\widetilde{\mathbf{Y}}^{\text{asc}}$ into one vector $\mathbf{Y} \in \mathbb{R}^{(N^{\text{trc}}+N^{\text{asc}})\times 1}$ and similarly we concatenate $\widetilde{\mathbf{X}}^{\text{trc}}$ and $\widetilde{\mathbf{X}}^{\text{asc}}$ into one matrix $\mathbf{X} \in \mathbb{R}^{(N^{\text{trc}}+N^{\text{asc}})\times p}$ where $p$ is the number of SNPs. To perform fine-mapping, we run `susieR::susie(X = X, Y = Y, intercept = FALSE, standardize = FALSE)` with `X` equal to $\mathbf{X}$ and `Y` equal to $\mathbf{Y}$. To build prediction model, we run `glmnet::glmnet(x = X, y = Y, lambda = lambda, alpha = 0.5)` with `x` equal to $\mathbf{X}$ and `y` equal to $\mathbf{Y}$. The hyperparamter `lambda` is selected by 5-fold nested cross-validation where at each `lambda` the 5-fold cross-validation are repeated three times and `lambda` that has lowest cross-validated mean squared error (averaged across three runs) is used. For comparison, we feed the part of total read count data $(\mathbf{X}^{\text{trc}}, \mathbf{Y}^{\text{trc}})$ directly into: 1) susieR for fine-mapping; and 2) elastic net for prediction. The procedure is the same but $\mathbf{X}, \mathbf{Y}$ are replaced by $\mathbf{X}^{\text{trc}}, \mathbf{Y}^{\text{trc}}$. And we call this total read count-only approach for fine-mapping and prediction as trcFine and trcPred.

## 6 Simulating RNA-seq reads

To examine the performance of the methods, we propose and implement a simulation scheme which generates total and allele-specific read counts. The simulation procedure includes three parts: 1) simulate gene body which will be aligned by reads; 2) randomly draw the causal variants; 3) simulate the number of reads for each haplotype transcript and place these reads to the gene body obtained in step 1). The total and allele-specific read counts can be directly read out from step 3) where the total read count is the sum of two haplotypic read counts and the allele-specific read count is the number of reads overlapping with heterozygous sites within gene body.

In step 1), we fix the length of gene body to be 10kbp. To simulate the heterozygous sites within gene body for each individual, we start with determining the position of polymorphic sites along gene body. We

first sample the number of polymorphic sites from Binomial distribution, and then draw their positions and minor allele frequencies (MAFs). And finally, whether a polymorphic site is heterozygous in an individual is determined by Bernoulli distribution with MAF. The procedure is sketched as follow.

1. Number of polymorphic site within gene body $N_h \sim \text{Binomial}(L_{\text{gene}}, f^h)$, where $L_{\text{gene}} = 10^4, f^h = 0.001$.
2. Position $P_m$ ($m = 1, \cdots, N_h$) of these polymorphic sites are sampled by $P_m \sim \text{Sample}(\{1, \cdots, L_{\text{gene}}\})$And the corresponding MAF $f_m$ are drawn from $f_m \sim \text{Uniform}(\text{maf}^l, \text{maf}^h)$, where $\text{maf}^l = 0.05, \text{maf}^h = 0.3$.
3. For each individual $i$, whether the $m$th polymorphic site is heterozygous (denote as $Z_{im}$) is determined by $Z_{im} \sim \text{Bernoulli}(2f_m(1 - f_m))$.

In step 2), the genetic effect equals to $e^{X_i^h\beta}$ (in single-SNP model) and $e^{\mathbf{X}_i^h\beta}$ (in multi-SNP model). To do so, we need to obtain haplotype and effect size. For single-SNP model, we first sample MAF of the causal variants and obtain the two haplotypes of each individual by drawing from Bernoulli. For multi-SNP model, we use the 1000G phase3 genotypes of European individuals. In brief, we randomly select 200 genes on chromosome 22 and extract phased genotypes of 1Mbp cis-window surrounding the transcription start site of them (excluding variants with allele frequency $< 0.01$ or $> 0.99$). The genetic effect size, $e^\beta$, ranges among 1, 1.01, 1.05, 1.1, 1.25, 1.5, 2, 3 for single-SNP case. In multi-SNP case, the number of causal SNPs is sampled from 1, 2, 3 and the genetic effect ranges from 0.015 to 0.075 such that the heritability ranges approximately from 19.4% to 54.5%. The detailed procedure for sampling $e^{X^h\beta}$ and $e^{\mathbf{X}_i^h\beta}$ is as follow.

- **Single-SNP scenario**:
  1. Sampling $X_i^h$: MAF of causal SNP $f^c \sim \text{Uniform}(\text{maf}^l, \text{maf}^h)$ and $X_i^h \sim \text{Bernoulli}(f^c)$ where $\text{maf}^l = 0.05, \text{maf}^h = 0.3$.
  2. Setting up $\beta$: fixed to 1, 1.01, ..., 2, 3.
- **Multi-SNP scenario**:
  1. Sampling $\mathbf{X}_i^h$: obtained from 1000G phased genotypes.
  2. Setting up $\beta$: number of causal SNPs $\sim \text{Sample}(\{1, 2, 3\})$ and the genetic variation $v_g \sim \text{Uniform}(0.015, 0.075)$. The genetic effect of causal variants are determined by randomly partition the genetic variation and convert per-SNP genetic variation into effect size by $\beta_k = \sqrt{v_{g,k}/(2f_k(1 - f_k))}$ where $f_k$ is MAF of $k$th causal SNP.

In the step 3), the last step, we sample the reads coming from each of the haplotype transcripts. The procedure is as follow.

1. For individual $i$, sample library size $L_i \sim \text{NegativeBinomial}(\text{size}, \text{prob})$ where $\text{size} = 15, \text{prob} = 1.6 \times 10^{-7}$ (Negative Binomial follows parameterization in `rnbinom` in R).
2. And then, sample individual-specific baseline abundance $\theta_{0,i} \sim$ Beta where $\text{E}(\theta_{0,i})$ ranges among $5 \times 10^{-5}, 2.5 \times 10^{-5}, 1 \times 10^{-5}, 5 \times 10^{-6}, 2.5 \times 10^{-6}, 1 \times 10^{-6}$ and $\text{sd}(\theta_{0,i}) = \text{E}(\theta_{0,i})/4$ (so that the non-genetic variation is roughly $1/4^2 = 1/16$).
3. The actual relative abundance of haplotype $h$ in individual $i$ is $\theta_i^h = \theta_{0,i} e^{X_i^h\beta}$ or $\theta_i^h = \theta_{0,i} e^{\mathbf{X}_i^h\beta}$
4. Sample actual read count for each haplotype: $Y_i^h \sim \text{NegativeBinomial}(\text{size}, \text{prob})$ where $\text{size} = 2L_i\theta_i^h, \text{prob} = \frac{2}{3}$. This corresponds to $\text{E}(Y_i^h) = L_i\theta_i^h$ and $\text{Var}(Y_i^h) = \frac{3}{2}\text{E}(Y_i^h)$.
5. Randomly place reads, $Y_i^h$ in total, onto the corresponding gene body simulated in step 1) where the read is aligned to each position of gene body with equal probability.
6. Total count is $Y_i^{\text{total}} = Y_i^1 + Y_i^2$ and allele-specific count $Y_i^{(h)\text{obs}}$ is the number of reads (as part of $Y_i^h$) that overlaps with the heterozygous sites of the individual (indicated by $Z_{i\cdot}$).

# 7 Pseudocode on solving trcQTL and ascQTL in matrix form

We sketch the matrix operations for solving a grid of least squares problems $\mathbf{y}_k \sim \mathbf{x}_j$ for each pair of $j, k$ where we let $Y = [\mathbf{y}_1, \cdots, \mathbf{y}_K]$ and $X = [\mathbf{x}_1, \cdots, \mathbf{x}_n]$. To obtain nominal p-value, $K = 1$. For permutation procedure proposed in fastQTL [12], $K$ equals to the number of permutation and $\mathbf{y}_k$ is the $k$th permuted $\mathbf{y}$.

To ensure trcQTL and ascQTL ran on the same permuted $\mathbf{y}$, we perform permutation before removing low count observations. So that in each permutation, different individuals are removed by low-count filter. To account for this fact, we introduce mask $M \in \{0, 1\}^{n \times K}$ where $M_{ik}$ indicating if the $i$th individual is included in $k$th permutation.

For trcQTL, the corresponding least squares problem has intercept, as mentioned in Eq 49. The pseudocode to solve the grid of trcQTL problems for all cis-SNP of a gene is sketched in Algorithm 1 where $Y = \mathbf{Y}^{\text{trc}}$ for nominal pass and $Y_{\cdot k} = P_k \mathbf{Y}^{\text{trc}}$ with permutation matrix $P_k$ for permutation pass.

Note that the pseudocode only requires basic matrix operation. The matrix operation is element-wise if not notice explicitly. The Einstein summation is represented by `einsum` with similar arguments as `numpy.einsum` in Python. For instance, `einsum('ij,jk→ik', A, B)` means that to take the inner product of the $i$ row in A and $k$ column in B as the element at $i$th row and $j$th column in the output matrix.

Similar to trcQTL, the corresponding least squares problem of ascQTL is weighted without intercept, as mentioned in Eq 50. The pseudocode to solve the grid of ascQTL problems for all cis-SNP of a gene is sketched in Algorithm 2 where $Y = \mathbf{Y}^{\text{asc}}$ for nominal pass and $Y_{\cdot k} = P_k \mathbf{Y}^{\text{asc}}$ with permutation matrix $P_k$ for permutation pass. And $W$ as the weight matrix should be permutate accordingly, *i.e.* $W_{\cdot k} = P_k \mathbf{w}$. And to obtain valid mixQTL estimates under permutation, $P_k$ is required to be shared by trcQTL and ascQTL in permutation pass.

Note that both Algorithm 1 and Algorithm 2 are iteration free. And throughout the computation, only two-way tensors are involved explicitly so that the memory usage does not blow up.

**Algorithm 2:** Solve multiple least squares problems $y = bx + e$ with weight $w$ in matrix form

**Input :** $Y \in \mathbb{R}^{n \times K},\ X \in \mathbb{R}^{n \times p},\ M \in \{0, 1\}^{n \times K},\ W \in \mathbb{R}_+^{n \times K}$.

**Output:** $\widehat{B} \in \mathbb{R}^{K \times p}$ and $\text{se}(\widehat{B}) \in \mathbb{R}^{K \times p}$ where $\widehat{B}_{kj}, \text{se}(\widehat{B}_{kj})$ are estimates of $Y_{\cdot k} = B_{kj} X_{\cdot j} + \epsilon$ where data is weighted by $W_{\cdot k}$ and masked by $M_{\cdot k}$.

**Function** `SolveMatrixLSwithWeight`$(Y, X, M, W)$**:**
- $n$ = einsum('ik→k', $M$);
- $W = WM$;
- $Y_{sqW} = Y\sqrt{W}$;
- $Y = YW$;
- $T$ = einsum('ij,ik→jk', $X$, $Y$);
- $S = X^2$;
- $S$ = einsum('ij,ik→jk', $S$, $W$);
- $\widehat{B} = T/S$;
- $Y_{sq}$ = einsum('ik,ik→k', $Y_{sqW}$, $Y_{sqW}$);
- $R_{sq} = Y_{sq} - 2\widehat{B}T + \widehat{B}^2 S_{11}$;
- $\widehat{\sigma} = \sqrt{R_{sq}/(n-1)}$;
- $\text{se}(\widehat{B}) = \widehat{\sigma}/\sqrt{S}$;
- **return** $\widehat{B}, \text{se}(\widehat{B})$

**End**

**Algorithm 1:** Solve multiple least squares problems $y = a + bx + e$ in matrix form

**Input :** $Y \in \mathbb{R}^{n\times K},\ X \in \mathbb{R}^{n\times p},\ M \in \{0,1\}^{n\times K}$.
**Output:** $\widehat{A}, \widehat{B}, \mathrm{se}(\widehat{A}), \mathrm{se}(\widehat{B}) \in \mathbb{R}^{K\times p}$ where $\widehat{A}_{kj}, \widehat{B}_{kj}, \mathrm{se}(\widehat{A}_{kj}), \mathrm{se}(\widehat{B}_{kj})$ are estimates of $Y_{\cdot k} = A_{kj} + B_{kj}X_{\cdot j} + \epsilon$ where data is masked by $M_{\cdot k}$.

**Function** `SolveMatrixLSwithIntercept`($Y, X, M$)**:**
  $U$ = matrix(1, dim = dim($X$));
  $n$ = einsum('ik→k', $M$);
  $Y = YM$;
  $T_1$ = einsum('ij,ik→jk', $X$, $Y$);
  $T_2$ = einsum('ij,ik→jk', $U$, $Y$);
  $S_{11} = X^2$;
  $S_{11}$ = einsum('ij,ik→jk', $S_{11}$, $M$);
  $S_{22} = U^2$;
  $S_{22}$ = einsum('ij,ik→jk', $S_{22}$, $M$);
  $S_{12} = XU$;
  $S_{12}$ = einsum('ij,ik→jk', $S_{12}$, $M$);
  $\Delta = |S_{11}S_{22} - S_{12}S_{12}|$;
  $\widehat{B} = (S_{22}T_1 - S_{12}T_2)/\Delta$;
  $\widehat{A} = (S_{11}T_2 - S_{12}T_1)/\Delta$;
  $Y_{sq}$ = einsum('ik,ik→k', $Y$, $Y$);
  $R_{sq} = Y_{sq} - 2\widehat{B}T_1 - 2\widehat{A}T_2 + 2\widehat{B}\widehat{A}S_{12} + \widehat{B}^2S_{11} + \widehat{A}^2S_{22}$;
  $\widehat{\sigma} = \sqrt{R_{sq}/(n-2)}$;
  $\mathrm{se}(\widehat{B}) = \widehat{\sigma}\sqrt{S_{22}/\Delta}$;
  $\mathrm{se}(\widehat{A}) = \widehat{\sigma}\sqrt{S_{11}/\Delta}$;
  **return** $\widehat{A}, \widehat{B}, \mathrm{se}(\widehat{A}), \mathrm{se}(\widehat{B})$
**End**

# 8 Evaluating QTL mapping performance using eQTLGen results

To evaluate the performance of QTL mapping method, we treat eQTLGen [13] as a silver standard, in the sense that eQTLs identified as positive in eQTLGen are treated as the true associations and the non-significant variant/gene pairs in eQTLGen are treated as true non-associations. Although 336 GTEx samples are included in eQTLGen analysis, they make up of only around 1.5% of total samples. So, eQTLGen results are unlikely driven by GTEx samples. And besides, GTEx v8 includes additional samples that are not included in eQTLGen. Therefore, eQTLGen is an approximately independent eQTL study with much larger sample size (50-fold relative to GTEx v8) and diverse populations (predominantly Europeans along with other populations).

To simplify the analysis, we randomly select 100,000 eQTLGen cis-eQTLs (FDR $< 0.05$) as the true associations in the silver standard. And we randomly collect 100,000 variant/gene pairs in eQTLGen with p-value $> 0.5$ as the true non-associations. Among those variant/gene pairs in silver standard, 96,660 true associations and 78,691 true non-associations are included in both our mixQTL mapping pipeline and GTEx v8 analysis. So that we keep only these variant/gene pairs for downstream analysis.

### 8.1 Comparing the effective sample size

To compare the effective sample size between mixQTL and eQTL approaches, we performed analysis similar to [14]. Here, we utilize the fact that $\chi^2$ statistic scales proportionally with the sample size, among those true associations. So, we can calculate the ratio $\chi^2_{\text{mixQTL}}$ over $\chi^2_{\text{eQTL}}$ for each truly associated variant/gene pair as the measure of effective sample size of mixQTL relative to eQTL approach. Specifically, we calculate the relative effective sample size using the true associations in the silver standard constructed above (as the proxy of true associations based on independent evidence). Note that the gain of power in mixQTL depends on the amount of allele-specific observations so we measured the average relative effective sample size as the median of the $\chi^2$ ratio. Among the 96,660 variant/gene pairs collected as true associations in silver standard, we measured the median of $\chi^2_{\text{eQTL}}$ as 2.59 and the median of $\chi^2_{\text{mixQTL}}$ as 3.56. And the median of the ratio $\chi^2_{\text{mixQTL}}$ over $\chi^2_{\text{eQTL}}$ is 1.29. In other word, it suggests that the mixQTL approach (with 670 individuals) is equivalent to the eQTL approach with 863 individuals.

### 8.2 Drawing receiver operating characteristic and precision-recall curves

The ROC and PR curves are constructed using $-\log(p)$ as prediction score (higher means more likely to be causal). To simplify the calculation, we evaluate the performance measures at a grid of score cutoffs: 0.1, 0.2, ..., 1.9, 2, 2.2, ..., 2.8, 3, 4, ..., 50. For ROC curve, we calculate true positive rate and false positive rate at these cutoffs. And similarly, for PR curve, we calculate precision and power at these cutoffs.

## 9 Running RASQUAL on GTEx data

We implemented the RASQUAL analysis pipeline for GTEx v8 data at `https://github.com/liangyy/run-rasqual` and ran RASQUAL on kidney cortex and whole blood data in GTEx v8. We focused on the genes with enough allele-specific reads. To ensure this, we required the genes to pass the following two criteria: 1. The gene should have more than 100 reads (total count) in at least 80% of the samples; 2. The gene should have $\geq 50$ allele-specific reads (per haplotypes and both haplotypes should meet the criteria) in at least 15 samples. With these criteria, we tested 4,596 genes in kidney cortex (sample size = 73) among 22 autosomes and 192 genes in whole blood (sample size = 670) on chromosome 22. Instead of using RASQUAL default parameters, we fixed two of the hyperparameters, $\delta$ (=0.5) and $\phi$ (=0.01), controlling mapping error rate and mapping bias. We made this choice for two reasons: 1. These two parameters are not considered in mixQTL analysis; 2. To estimate these parameters take time and by fixing these the running time for RASQUAL reduced substantially. RASQUAL was run with 8 CPU cores and 16gb RAM.

## 10 Examining the enrichment in functional annotations

We focused the analysis on 26 GTEx v8 tissues which have sample size $< 260$. Furthermore, we focused on the genes with sufficient amount of allele-specific counts. Specifically, for each tissue, we selected the genes passing the criteria described in Supplementary Notes 9.

Regarding the functional annotation, we included the functional annotation constructed by GTEx v8 working group (see more details in [1] supplementary notes section 9). We also looked at the candidate Cis-Regulatory Elements (cCREs) in ENCODE [2] where we manually selected ENCODE tissue/cell line that matches with the GTEx tissue. With this restrictive matching, we included 10 of the 26 tissues for the cCRE enrichment analysis. Moreover, to ensure the quality of the annotation, we excluded the cCREs that are labelled as “Unclassified”. Lastly, we also considered GWAS catalog where we label GWAS catalog variant as 1 and the rest of the genome as 0.

Since all these annotations are binary, for each functional annotation, we formed a 2-by-2 table (functional annotation against whether the variant is top signal in mixQTL or mixFine) aggregating across all

tissues. The enrichment in functional annotation was measured as the odds ratio calculated on the basis of the 2-by-2 table.

# Supplementary References


[1] The GTEx Consortium. The gtex consortium atlas of genetic regulatory effects across human tissues. *Science* **369**, 1318–1330 (2020).

[2] Moore, J. E. *et al.* Expanded encyclopaedias of dna elements in the human and mouse genomes. *Nature* **583**, 699–710 (2020).

[3] Kumasaka, N., Knights, A. J. & Gaffney, D. J. Fine-mapping cellular qtls with rasqual and atac-seq. *Nature genetics* **48**, 206 (2016).

[4] Mohammadi, P., Castel, S. E., Brown, A. A. & Lappalainen, T. Quantifying the regulatory effect size of cis-acting genetic variation using allelic fold change. *Genome research* **27**, 1872–1884 (2017).

[5] Shabalin, A. A. Matrix eqtl: ultra fast eqtl analysis via large matrix operations. *Bioinformatics* **28**, 1353–1358 (2012).

[6] Lee, C. H., Cook, S., Lee, J. S. & Han, B. Comparison of two meta-analysis methods: inverse-variance-weighted average and weighted sum of z-scores. *Genomics & informatics* **14**, 173 (2016).

[7] Gamazon, E. R. *et al.* A gene-based association method for mapping traits using reference transcriptome data. *Nature genetics* **47**, 1091 (2015).

[8] Wang, G., Sarkar, A., Carbonetto, P. & Stephens, M. A simple new approach to variable selection in regression, with application to genetic fine mapping. *Journal of the Royal Statistical Society: Series B (Statistical Methodology)* **82**, 1273–1300 (2020).

[9] Lee, Y., Francesca, L., Pique-Regi, R. & Wen, X. Bayesian multi-snp genetic association analysis: Control of fdr and use of summary statistics. *bioRxiv* 316471 (2018).

[10] Kang, H. M. *et al.* Efficient control of population structure in model organism association mapping. *Genetics* **178**, 1709–1723 (2008).

[11] Loh, P.-R. *et al.* Efficient bayesian mixed-model analysis increases association power in large cohorts. *Nature genetics* **47**, 284 (2015).

[12] Ongen, H., Buil, A., Brown, A. A., Dermitzakis, E. T. & Delaneau, O. Fast and efficient qtl mapper for thousands of molecular phenotypes. *Bioinformatics* **32**, 1479–1485 (2015).

[13] Võsa, U. *et al.* Unraveling the polygenic architecture of complex traits using blood eqtl meta-analysis. *bioRxiv* 447367 (2018).

[14] Loh, P.-R., Kichaev, G., Gazal, S., Schoech, A. P. & Price, A. L. Mixed-model association for biobank-scale datasets. *Nature genetics* **50**, 906–908 (2018).
