# Supplementary material for: A scalable unified framework of total and allele-specific counts for cis-QTL, fine-mapping, and prediction
Source: Nat Commun. 2021 Mar 3;12:1424. doi: 10.1038/s41467-021-21592-8 (PMC7930098; doi:10.1038/s41467-021-21592-8)
Supplement: Supplementary file 4 — Description of Additional Supplementary Fiies [file 41467_2021_21592_MOESM4_ESM.pdf]

Description of Additional Supplementary Files

Supplementary Data 1: The links to the full summary statistics of mixQTL on GTEx v8
The mixQTL summary statistics are publicly available via the DOI's and URL's listed in the table. 49 tissues of GTEx v8 are included.
