# Supplementary material for: A scalable unified framework of total and allele-specific counts for cis-QTL, fine-mapping, and prediction
Source: Nat Commun. 2021 Mar 3;12:1424. doi: 10.1038/s41467-021-21592-8 (PMC7930098; doi:10.1038/s41467-021-21592-8)
Supplement: Supplementary file 2 — Peer Review [file 41467_2021_21592_MOESM2_ESM.pdf]

Reviewer #1 (Remarks to the Author):

Liang et al. developed an efficient computational framework that combines total and allele-specific gene expression for eQTL studies. Specifically, they developed three tools, mixQTL, mixFine, and mixPred for finding eQTLs, performing fine-mapping, and prediction of gene expression data, respectively. The authors showed using simulated and GTEx data that combining total and allele-specific gene expression improves the performance of all three tasks (QTL mapping, fine-mapping, and prediction), compared to methods that use only total or allele-specific information. The statistical models described in the manuscript are sound with detailed derivation process. The results of analysis are well presented, and the computational framework proposed in this manuscript has potential to be useful for the research community for analyzing gene expression data. However, the main idea in this paper, which is to combine total and allele-specific gene expression, is not very novel as it was utilized in a number of different tools as the authors mention. Hence, it appears that this framework is a combination of previous approaches without vast improvement over those approaches except that it is more efficient, which is not clearly demonstrated in the paper as discussed below. I also have following major concerns about results and methods.

1. One major limitation of mixQTL is that it can be only applied to 28% of genes in GTEx data, and it outperforms standard QTL mapping only for middle or high expression groups among those 28% of genes without mentioning how many genes are in those groups. Also, it is possible that other methods that combine total expression and ASE for eQTL mapping such as RASQUAL or WASP may be applied to more genes and detect more eQTLs. The authors need to elaborate more on this issue.

2. Regarding the previous comment, it is not clear why the authors chose not to apply RASQUAL and WASP to the GTEx data for eQTL mapping as they can be applied to a dataset with a few hundred people, which is the sample size of the GTEx data. The authors should compare the number of eQTLs detected by these methods on the GTEx data or eQTLGen data with that by mixQTL.

3. One of the major claims by the authors is that their method is much more efficient than previous approaches saying their method is 10 times faster than the next fastest algorithm. To show speed gain over previous approaches, the authors, however, chose to apply the method to simulated data with only 100 samples, and the real data they analyzed (GTEx data) has the sample size of a few hundreds. The authors should also show the runtime gain as the sample size increases up to a thousand or more to see if they continue to have large speed gain as other approaches can still be applied to small sample size datasets.

4. For the fine mapping method (mixFine), the authors should compare their method to previous approaches such as one by Zou et al. or Wang et al. that also use both total expression and ASE data for fine-mapping. These previous approaches already showed that combining total expression and ASE data improves the accuracy of fine-mapping, so the results of mixFine in the paper are expected. Instead, the authors should show whether their method outperforms or performs similarly to the previous approaches that use both total expression and ASE.

5. I have several concerns for the prediction method (mixPred). First of all, the authors should discuss utility of mixPred or a gene expression prediction method in general as it is not clear why one wants to use this method (is it useful in improving power of eQTL studies?). Second, it is not clear whether there is any use of this method as the Pearson correlation is not very impressive for most genes (<0.3). Is this good enough such that we can use this prediction for eQTL mapping or any other analysis? Lastly, it is hard to tell how much better mixPred is compared to the standard prediction method in Figure 6B; the authors should come up with some quantitative metrics for this figure.

6. Regarding the methods, there are two main assumptions in this approach. One is that effect size from total expression and that from ASE are independent (beta^trc in equation 62 and beta^asc in equation 63). The authors mention they are “approximately independent” but did not provide

theoretical derivation or empirical data supporting this claim. The authors also did not show how much they are independent. If they are not independent, meta-analysis using these two beta values will cause false positives. Hence, the authors should elaborate on why and how much these two beta values are independent.

7. Another assumption in this method is weak genetic effects. This may be true for SNPs found in GWAS, but some SNPs in eQTL studies may have large effect sizes. The authors need to discuss what problems may be caused if this assumption is violated and how likely this assumption is violated in eQTL studies.

8. Fine-mapping simulation needs more explanation. For example, how many causal variants are assumed? Is it one or more? Also, Fig 3A shows trcFine seems to have higher fraction of true positives across PIP bins. Does this mean mixFine have higher false positives? And Fig 3C, can you calculate average size of causal sets and compare it between mixFine and trcFine? It is not clear whether the mean is significantly different between the two.

Reviewer #2 (Remarks to the Author):

This paper describes a new statistical methods to discover/fine-map expression quantitative trait loci (eQTLs) by leveraging two different sources of information: total and allele-specific gene expression. In addition, an approach to predict gene expression from trained models is also described. The authors extensively compared their approach to a standard model only based on total gene expression and show that it performs better in all three tasks: discovering/fine-mapping eQTLs and predicting gene expression from genetic data.

In terms of form, I think the paper is well written and presented: the figures are clear and the text flows very well. The resulting paper is easy and pleasant to read. In terms of content, the method seems to constitute a nice addition to the eQTL toolbox but lacks evidence of its tractability and benefit on real datasets.

Specifically, I do have the following concerns regarding the tests performed on real data:

1. Mapping eQTLs. I think the comparison with other methods should be extended, notably on GTEx, so that it covers more than 4 lines of text and one supplementary figure. It would also be good to see how mixQTL performs at scale (why not the full GTEx v8.0 data set?). This would provide solid evidence of its practicability compared to other methods (using or not allele specific signal) and some idea of the computational effort needed to get this additional set of eQTLs (how long do we need for how many new eQTLs/eGenes?).

2. Fine-mapping. I'd be curious to see where the causal variants spotted by each respective approach do locate in terms of functional annotations (e.g. Encode).

3. Gene expression prediction. How does mixQTL perform relative to PrediXcan? Also, I cannot really see any difference between "standard" and "mixPred" in Fig6B: the authors should support their claim with a better figure.

4. Using the eQTLGen dataset as validation is a reasonable approach as it was derived from a huge sample size. However, there is quite a bit of heterogeneity in this dataset and I would be cautious when using it as a "ground truth": I would seriously consider replicating the results on several randomizations of the 100,000 variant-gene pairs to make sure that the results still hold.

5. What is the computational complexity of the overall algorithm? Is it linear with number of samples?

Quadratic?

Overall, this work nicely demonstrates on simulated data that leveraging allele specific signal does improve discovery power, fine-mapping and prediction. However, I think this should be better illustrated on real data with some comparisons with standard methods commonly used in the field.

Reviewer #3 (Remarks to the Author):

The paper introduces a principled way to convert joint analysis of gene expression and ASE for eQTL calling, fine mapping, and prediction of genetically driven expression into a meta-analysis problem over independent linear regression. I like the work: I think modeling is elegant, and the addressed problems are relevant. That said, I think the work as it stands is not mature enough for publication.

Specifically, most analyses reported are simulations, and the current results from real data support a fraction of the claims and do not provide any new biological results/insights. Simulation results are important for debugging the code and exploring the model's behavior but are not helpful in testing the validity of the model assumptions, comparison to other methods. The authors claim improvements in eQTL calling, fine mapping and expression prediction. While each of these contributions could be sufficient for a separate paper, the manuscript fails to pin down the performance and practical value of each of these methods and provide the new biological insights with adequate experiments on real data. The paper should address the following practical questions for a reader:

1: When should one use mixQTL on a dataset instead of standard TensorQTL, or WASP/RASQUAL for eQTL calling? How are the eQTLs found with mixQTL different from those found by the conventional approach quantitatively and functionally. The simulations are adequate for the speed comparisons, and the analysis of GTEx data in Figure 5 highlights the potential value of the method or the standard eQTL calling. Is the value over WASP/RASQUAL here is only the speed? Now that the speed issue is resolved what do we find in GTEx v8 that we were unable to find before? Considering the motivation of the paper regarding the need for a faster method: "However, these methods are computationally too costly to be applied to sample sizes beyond a few hundred and as a result have not been applied to large-scale studies like GTEx, which includes over 17,000 samples across 49 tissues." it would be reasonable to expect the results to include the results from the complete GTEx data and the new biological insights found by this new analysis. Providing the results from the complete GTEx data would also be also a great resource for the community.

2: Should one use mixFine instead of PLASMA or the Zou et al. 2019 methods, which are both very similar fine-mapping techniques designed to utilize ASE and the aFC model.

3: Should one use mixPred instead of the standard Susie/elastic net for predicting genetically driven gene expression? This analysis is presented in figure 6B. But, I cannot understand how Figure 6B shows an improvement? Please clarify if this is the case with appropriate visualization/analysis.

I realize that the above questions have been partially explored via simulation. Still, I believe a real data comparison using orthogonal sources of evidence such as functional enrichment, reproducibility, etc. will be the appropriate way to evaluate the methods.

Here are more minor comments:

- The Abstract and Introduction sections go back and forth between the fine mapping and eQTL calling, starting from the 2nd and the 3rd sentence in the abstract. Please streamline the narrative to improve readability.

- The justification for error definitions on line 65 is not clear; the same goes for e^asc in line 67. Are the shared variance term and the variance scaling by mean appropriate assumptions that fit the real data? Does this assume that the biological variance to be similar in allelic imbalance and gene expression?

- I find the discussion regarding the "technical" and "biological noise" terms rather confusing (text between lines 66 and 67). Is it possible to plot these terms against each other in an example dataset by estimating the over-dispersion and count noise?

- In all relevant figures, please clarify the error bars (SE, 95% CI, std, Quantiles, ...).

- It is not clear in the text if mixQTL finds multiple independent eQTLs per gene or just the top one. Or if it needs to be used together with mixFine to find those?

- In the fine-mapping section, what fraction are "consensus snps"? Are the fine map SNPs from mixFine functionally similar/different to/from those from Susie? What about those high confidence SNPs from Susie that do not show up in mixFine?
- There are invalid characters substituted for $\leq$ in supplementary materials, see the paragraph before section 14.1.

Reviewer 1

Reviewer's comments	Response
Liang et al. developed an efficient computational framework that combines total and allele-specific gene expression for eQTL studies. Specifically, they developed three tools, mixQTL, mixFine, and mixPred for finding eQTLs, performing fine-mapping, and prediction of gene expression data, respectively. The authors showed using simulated and GTEx data that combining total and allele-specific gene expression improves the performance of all three tasks (QTL mapping, fine-mapping, and prediction), compared to methods that use only total or allele-specific information. The statistical models described in the manuscript are sound with detailed derivation process. The results of analysis are well presented, and the computational framework proposed in this manuscript has potential to be useful for the research community for analyzing gene expression data. However, the main idea in this paper, which is to combine total and allele-specific gene expression, is not very novel as it was utilized in a number of different tools as the authors mention. Hence, it appears that this framework is a combination of previous approaches without vast improvement over those approaches except that it is more efficient, which is not clearly demonstrated in the paper as discussed below. I also have following major concerns about results and methods.	We thank the reviewer for the helpful comments and suggestions. We address each point below.
**MAJOR COMMENTS**	
**1)** One major limitation of mixQTL is that it can be only applied to 28% of genes in GTEx data, and it outperforms standard QTL mapping only	We realize that our write up may have been unclear. In fact, mixQTL can be applied to all genes regardless of the number of allele-specific counts (ASCs). When the allele-specific count is low, the method

for middle or high expression groups among those 28% of genes without mentioning how many genes are in those groups.	defaults to using only total read count. In this paper, to make more clear the benefit of adding allele-specific counts, we show the comparison between approaches only for the 28% of the genes (5,734 genes) with enough ASCs to make a difference. For the remaining genes, the method simply outputs the results based on total read count, which we have shown that performs similarly to the standard eQTL approach (Fig.S8).<br><br>**Action:**<br>We have rewritten the paragraph on the mixQTL performance in GTEx to make this point more clear.<br><br>Also, for this revision, we have performed mixQTL analysis for all the genes that were tested by the GTEx consortium's standard eQTL analysis (about 24k genes per tissue in 49 tissues).
Also, it is possible that other methods that combine total expression and ASE for eQTL mapping such as RASQUAL or WASP may be applied to more genes and detect more eQTLs. The authors need to elaborate more on this issue.	We agree that RASQUAL/WASP may be able to identify more eQTLs since they model count data directly instead of using an approximation as mixQTL does. But their computation burden is too high for large datasets.<br><br>Implementing RASQUAL for GTEx data takes substantial effort. Following the reviewer's suggestion, we implemented the pipeline at https://github.com/liangyy/run-rasqual and ran RASQUAL on GTEx whole blood samples (n = 670, 192 genes). It took 826 seconds / gene on average. (see more details below). Given this computation time, we decided to run RASQUAL in GTEx kidney cortex tissue (n = 73).<br><br>**Action:**<br>We added to the discussion that RASQUAL yielded more significant results but given the much longer computational time, its applicability is

<table>
<tr><td></td><td>limited to small to medium size datasets. We also added the QQ-plot below to the supplement Fig.S11.
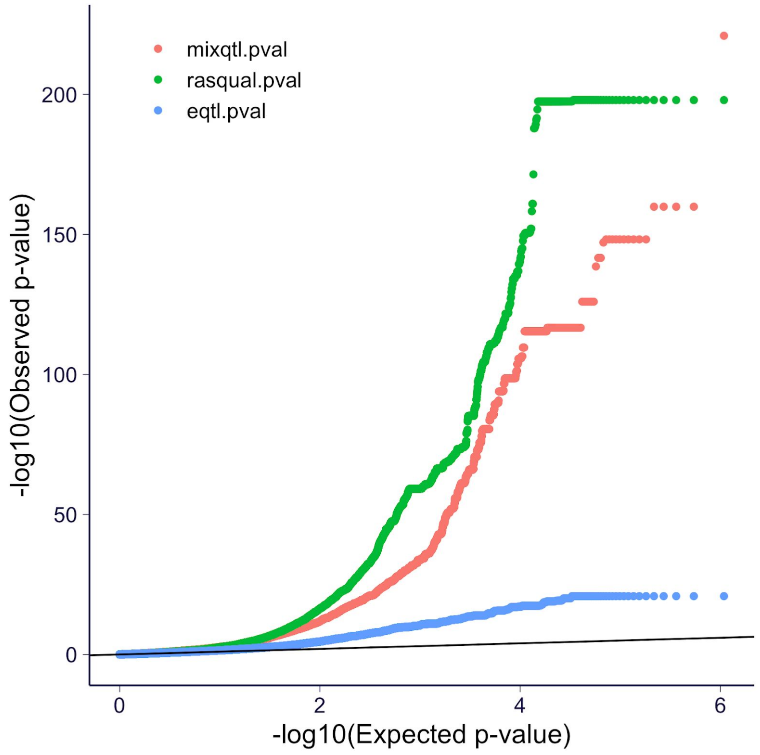
</td></tr>
<tr><td>2) Regarding the previous comment, it is not clear why the authors chose not to apply RASQUAL and WASP to the GTEx data for eQTL mapping as they can be applied to a dataset with a few hundred people, which is the sample size of the GTEx data. The authors should compare the number of eQTLs detected by these methods on the GTEx data or eQTLGen data with that by mixQTL.</td><td>As mentioned above, we ran RASQUAL on GTEx kidney cortex tissue and showed that RASQUAL yielded more significant p-values. However, we did not compare to eQTLGen results given the difference in tissue.</td></tr>
</table>

**3)** One of the major claims by the authors is that their method is much more efficient than previous approaches saying their method is 10 times faster than the next fastest algorithm. To show speed gain over previous approaches, the authors, however, chose to apply the method to simulated data with only 100 samples, and the real data they analyzed (GTEx data) has the sample size of a few hundreds.<br><br>The authors should also show the runtime gain as the sample size increases up to a thousand or more to see if they continue to have large speed gain as other approaches can still be applied to small sample size datasets.	We followed the reviewer's suggestion and as mentioned above, we ran RASQUAL in GTEx kidney cortex (4596 genes) and whole blood (192 genes). We found that in the Kidney Cortex mixQTL was 723 times faster than RASQUAL and in Whole Blood it was 2480 times faster.<br><br>This shows that in practice with real data, the gain in computational time is much larger than what we found with simulated data where covariates were not included, and other prior calculation steps were skipped, which makes the comparison incomplete and could be misleading. So we will drop the simulated data comparison and only show the real data computational time comparison.<br><br>**Action:**<br>We added these run times to the results section and dropped the simulated data comparison.
**4)** For the fine mapping method (mixFine), the authors should compare their method to previous approaches such as one by Zou et al. or Wang et al. that also use both total expression and ASE data for fine-mapping. These previous approaches already showed that combining total expression and ASE data improves the accuracy of fine-mapping, so the results of mixFine in the paper are expected. Instead, the authors should show whether their method outperforms or performs similarly to the previous approaches that use both total expression and ASE.	Zou et al and Wang et al combine the eQTL and ASE z-scores even though the eQTL and ASE results are obtained from completely different tests and definitions of expression level (read count or inverse normalized count). In contrast, mixFine is derived directly from the likelihood of the data so it provides theoretical justification of the approach. We expect that, in principle, mixFine should produce results similar to Zou et al and Wang et al.<br><br>However, we were not able to perform the comparison since both software implementations had bugs or other errors that were not easily solvable. We opened issues in their respective github repositories but none of the authors of these methods were responsive to our requests for help.

	For Wang et al method, we found that the code has some missing components. At least one other user had the same problem we encountered and opened an issue in the GitHub repository. We followed up on the same issue but have not heard back. The issue is at https://github.com/austintwang/plasma/issues/3#issuecomment-661161283.<br><br>For Zou et al methods, we packed their pipeline up into an R package https://github.com/liangyy/finemapAim but during testing their code, we found an unexpected behavior of their code, the output changes when we change the ID of one sample in the input. We opened an issue in their GitHub repository https://github.com/jzou1115/aim/issues/1) but have not heard back yet.
**5)** I have several concerns for the prediction method (mixPred).<br><br>First of all, the authors should discuss utility of mixPred or a gene expression prediction method in general as it is not clear why one wants to use this method (is it useful in improving power of eQTL studies?).<br><br>Second, it is not clear whether there is any use of this method as the Pearson correlation is not very impressive for most genes (<0.3). Is this good enough such that we can use this prediction for eQTL mapping or any other analysis?<br><br>Lastly, it is hard to tell how much better mixPred is compared to the standard prediction method in Figure 6B; the authors should come up with some quantitative metrics for this figure.	The goal of the prediction is to estimate the genetically determined gene expression rather than to obtain the best predictor of the observed expression. Such prediction models are key components of PrediXcan (https://www.nature.com/articles/ng.3367) and other transcriptome-wide association methods. And they can be viewed as the instrument variables in the Mendelian randomization framework.<br><br>Regarding the concern about the prediction performance, we want to make two points. First, the prediction performance of a gene is limited by the genetic architecture of the gene including the cis-heritability (proportion of gene expression captured by cis-variants). So, the prediction performance varies across genes because of such intrinsic biological properties. Second, Pearson correlation < 0.3 is typical in gene expression prediction models (Figure 2 in https://doi.org/10.1101/2020.03.19.997213). And with such prediction performance, the gene-level associations (based on S-PrediXcan) prioritizes likely causal genes (implicated in OMIM or rare variant

based association studies) (Figure 4 in https://doi.org/10.1101/2020.03.19.997213).

Action:

We added some discussion about the mixPred prediction models in the introduction to clarify the motivation.

To improve visualization, we report now the median prediction performance in the supplementary table S2. We also changed figure 6B with violin plots showing the increased pearson correlation for different sample sizes.

sample_size	pairwise_diff	diff_ci95_low	diff_ci95_high	pval	median_mixpred	median_standard
67	0.05208	0.04687	0.0573	1.318e-72	0.1753	0.07011
75	0.04952	0.04386	0.05518	4.828e-58	0.1854	0.07935
84	0.04889	0.04357	0.05422	4.569e-63	0.198	0.1003
96	0.0473	0.04187	0.05274	1.35e-57	0.2141	0.1189
112	0.04332	0.0381	0.04854	4.884e-53	0.228	0.1519
134	0.0362	0.03104	0.04136	1.483e-39	0.2413	0.1953
168	0.02762	0.02278	0.03246	1.791e-27	0.2514	0.2192
224	0.01657	0.01198	0.02115	2.535e-12	0.2663	0.2536
335	0.006543	0.002176	0.01091	0.003354	0.2919	0.2868

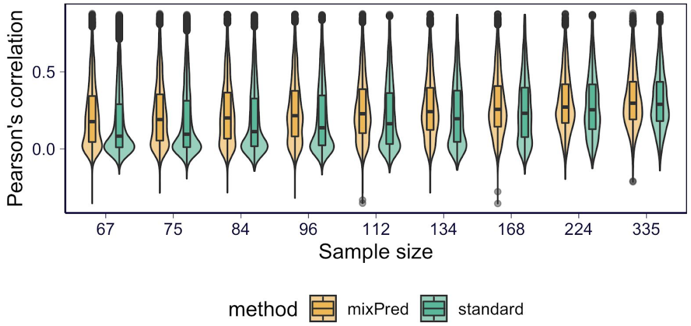

**6)** Regarding the methods, there are two main assumptions in this approach. One is that effect size from total expression and that from ASE are independent (beta^trc in equation 62 and beta^asc in equation 63). The authors mention they are “approximately independent” but did not provide theoretical derivation or empirical data supporting this claim. The authors also did not show how much they are independent. If they are not independent, meta-analysis using these two beta values will cause false positives. Hence, the authors should elaborate on why and how much these two beta values are independent.	The key assumption is that the counts from each of the two haplotypes are independent (conditioning on zi and library size), which is quite reasonable to us. From that, the independence of the sum and differences of the log counts follows. The weak effect size approximation means that the log of the total read count can be approximated by the sum of the log of haplotypic counts, and therefore it is approximately independent of the difference of the log of the haplotypic counts. Then the approximate independence of Eqn 50 and 51 follows (the correlation between ei^trc and ei^asc is zero).<br><br>**Action:**<br>We added this streamlined explanation to the results and the methods. We added the above discussion in the manuscript and made some edits accordingly (supplementary notes 10.1).
**7)** Another assumption in this method is weak genetic effects. This may be true for SNPs found in GWAS, but some SNPs in eQTL studies may have large effect sizes. The authors need to discuss what problems may be caused if this assumption is violated and how likely this assumption is violated in eQTL studies.	Our estimated effect sizes (log allelic fold changes) have a median absolute value of 0.153 and a 95th percentile of 0.845, which can be considered reasonably small. For discovery, large effects can be detected even if we have a small misspecification in the model. We also know that there is no obvious inflation of significance. Taken together, the weak effect assumption seems to be quite reasonable.

	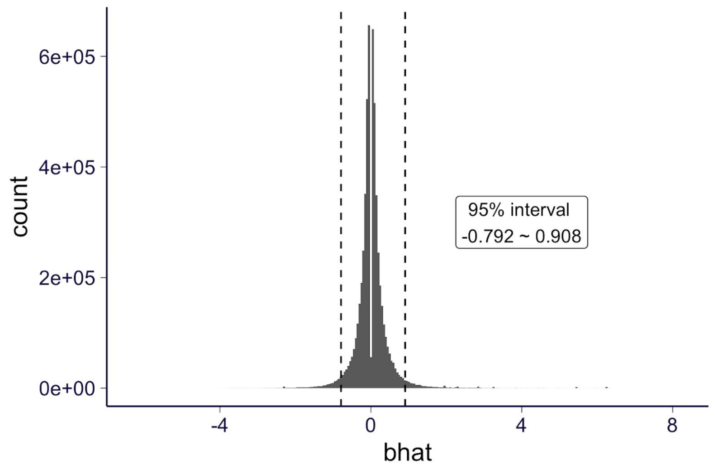 **Action:**<br>We added this figure as supplementary figure S15 and added it to the discussion in manuscript.
**8)** Fine-mapping simulation needs more explanation. For example, how many causal variants are assumed? Is it one or more?	We moved more of the details of the simulation to the main text.<br>In the paper, we simulated under two scenarios: single-SNP and multi-SNP scenarios which correspond to simulating only 1 causal SNPs and simulating up to 3 causal SNPs. The former is used to test mixQTL and the latter is used to test mixFine and mixPred. These simulated details are in the method section 6.7 and supplementary notes section 12.<br><br>**Action:**<br>And we moved more of the details to the main text now.
Also, Fig 3A shows trcFine seems to have higher fraction of true positives across PIP bins. Does this mean mixFine have higher false positives?	Prompted by this comment, we revisited our approach and obtained a better calibrated posterior inclusion probabilities (see details below).

ORIGINAL FIG 3A

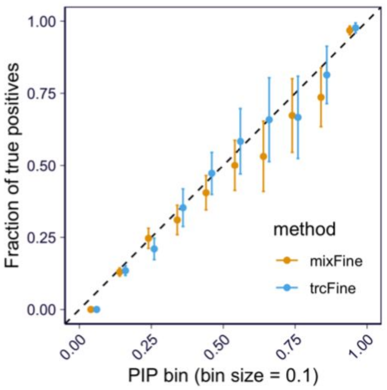

REVISED FIG 3A

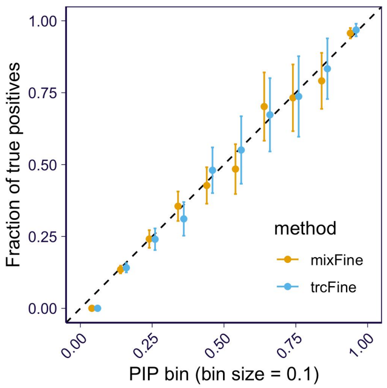

Specifically, now we estimate the variance of the noise term via random/mixed effect model where the genetic effects (beta_1, …, beta_P for P SNPs in the cis-window) are treated as random effect with beta_p ~iid N(0, Vg).

Or equivalently, we assume y ~ N(mu, Ve I + Vg X X’) with y being response and X being genotype (mean or difference of two haplotypes) and mu is the intercept in total read count equation and mu = 0 for allele-specific count equation.

Furthermore, to account for the intercept term in the total read count equation, we center y and column of X (subtract the mean), which, as a computation trick, has been deployed previously by (ref1: matrixEQTL, ref2: bolt-lmm). To sum up, the new way to perform step 1 is:

1. Estimate sigma^2 in allele-specific data by estimating the variance component in random effect model y ~ N(0, sigma^2 I / W + Vg X X’);

	2. Estimate sigma0^2 in total count data by estimating the variance component in the mixed effect model y ~ N(mu, sigma0^2 I + Vg X X’);<br>3. tilde_X^asc = sqrt(W) * X^asc / sigma^2<br>tilde_Y^asc = sqrt(W) * Y^asc / sigma^2<br>tilde_Y^trc = center(Y^trc / sigma0^2)<br>tilde_X^trc = center_by_column(X^trc / sigma0^2)<br>These equations above will replace the ones in Eq 70-71 in supplementary notes 11.2 and 11.3.<br><br>**Action:**<br><br>We added the following explanation to the methods section.<br>With the updated algorithm, we have updated Fig3A.<br>And we also updated all figures that are based on mixFine and mixPred results.
And Fig 3C, can you calculate average size of causal sets and compare it between mixFine and trcFine? It is not clear whether the mean is significantly different between the two.	To improve the visualization of the difference, for each true signal, we plot the 95% credible set size of trcFine against the mixFine CS size across all simulation parameter settings. The figure now shows more clearly that the 95% CS of mixFine is significantly smaller than the one of trcFine (paired t test p-value = 5.9e-29).

	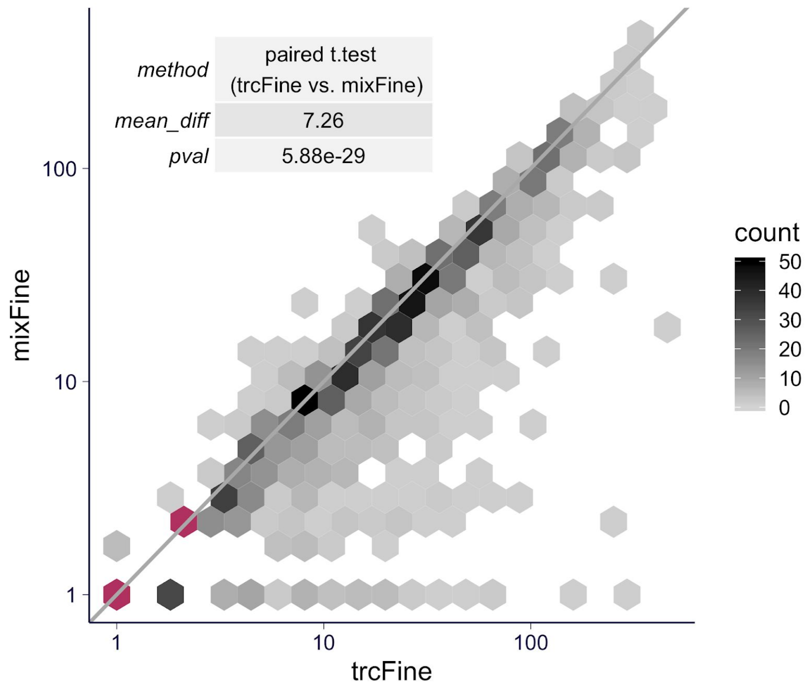<br>**Action:**<br>We updated the figures in the manuscript.

Reviewer 2

Reviewer's comments	Response
This paper describes a new statistical methods to discover/fine-map expression quantitative trait loci (eQTLs) by leveraging two different sources of information: total and allele-specific gene expression. In addition, an approach to predict gene expression from trained models is also described. The authors extensively compared their approach to a	We thank the reviewer for the helpful comments and suggestions. We address each point below.

standard model only based on total gene expression and show that it performs better in all three tasks: discovering/fine-mapping eQTLs and predicting gene expression from genetic data.<br><br>In terms of form, I think the paper is well written and presented: the figures are clear and the text flows very well. The resulting paper is easy and pleasant to read. In terms of content, the method seems to constitute a nice addition to the eQTL toolbox but lacks evidence of its tractability and benefit on real datasets.<br><br>Specifically, I do have the following concerns regarding the tests performed on real data.<br><br>Overall, this work nicely demonstrates on simulated data that leveraging allele specific signal does improve discovery power, fine-mapping and prediction. However, I think this should be better illustrated on real data with some comparisons with standard methods commonly used in the field.	
**MAJOR COMMENTS**	
**1)** Mapping eQTLs. I think the comparison with other methods should be extended, notably on GTEx, so that it covers more than 4 lines of text and one supplementary figure.	In this revision, in addition to the comparison with the standard eQTL approach, we add comparison with RASQUAL.<br><br>Given the computational burden (more than 10 days according to the authors of RASQUAL), we ran RASQUAL in a restricted set of genes in kidney cortex (4596 genes) and in whole blood (192 genes).

RASQUAL yields more significant results as shown in the QQ-plot below. We also show the comparison of estimated effect sizes further below, which shows reasonable concordance.

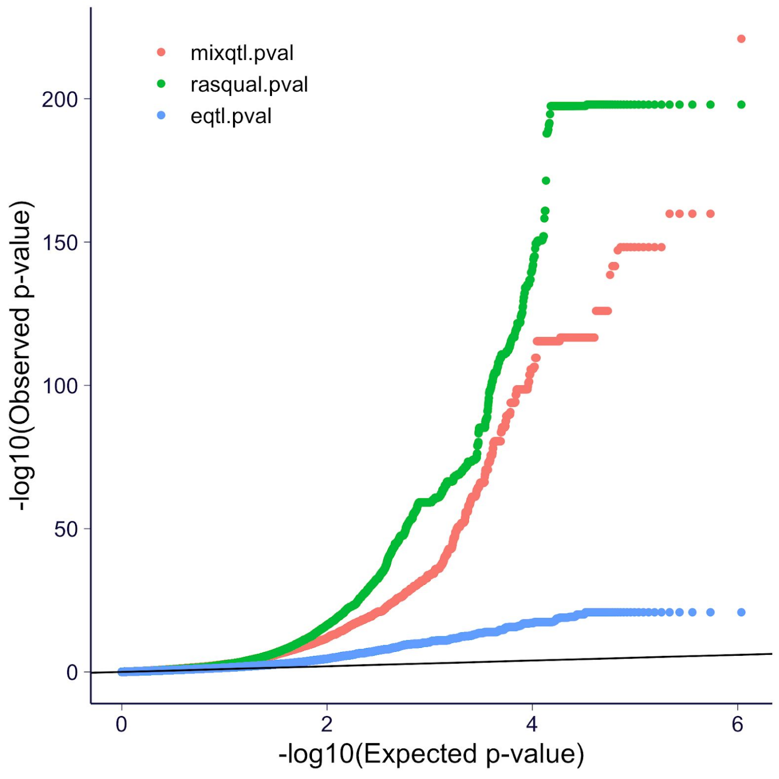

<table>
<tr>
<td></td>
<td>
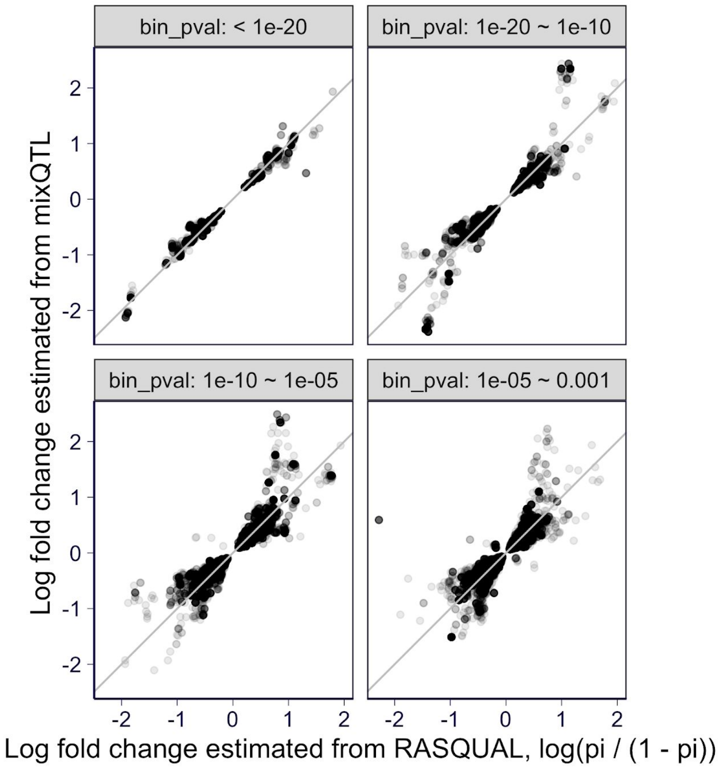

Action:
We added these results to the manuscript and the figures were added as supplementary figures S11.
</td>
</tr>
<tr>
<td>It would also be good to see how mixQTL performs at scale (why not the full GTEx v8.0 data set?). This would provide solid evidence of its practicability compared to other methods (using or not allele specific signal) and some idea of the computational effort needed to get this</td>
<td>We followed the suggestion and ran mixQTL on the 49 tissues analyzed by the GTEx v8 release. The full analysis included 15,201 samples covering 49 tissues and it took about 54 clock hours in total (used 8 CPU cores and 16gb RAM). The tissue with largest sample</td>
</tr>
</table>

additional set of eQTLs (how long do we need for how many new eQTLs/eGenes?).

size (n = 706) took 0.34 seconds per gene on average (for 1 Mbp cis-window). We included all genes regardless of its expression level.

See details of the implementation in Method section 6.8.

At FDR cutoff 0.05, on average, mixQTL identified 1440 more genes and 618k more eQTLs than the standard eQTL approach. The gain by tissue is shown in the figures below.

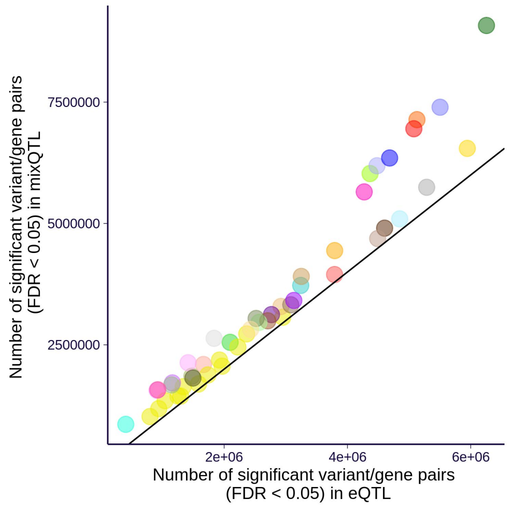

	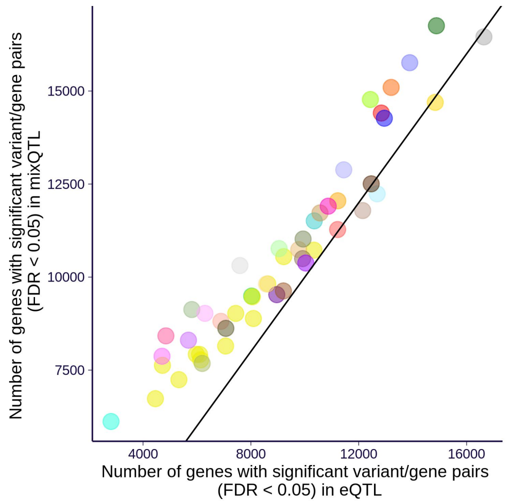<br>**Action:**<br>We shared the full summary statistics of mixQTL in the links listed in supplementary table S1.<br>We added the figures as the supplementary figure S12.
**2)** Fine-mapping. I'd be curious to see where the causal variants spotted by each respective approach do locate in terms of functional annotations (e.g. Encode).	Following the reviewer's suggestion, we ran mixFine and standard eQTL approach in 26 tissues with small sample sizes (n<260) where we thought that the gains would be most apparent. And we examined the enrichment of top QTL/PIP in different functional annotations.<br><br>We found that the top QTLs and top PIP variants from mixQTL and mixFine were more enriched among GWAS catalog variants and candidate cis-regulatory elements (https://www.nature.com/articles/s41586-020-2493-4) than the standard eQTL and fine-mapping methods. We did not find significant differences in enrichment differences for enhancer, promoter, and transcription factor binding sites.

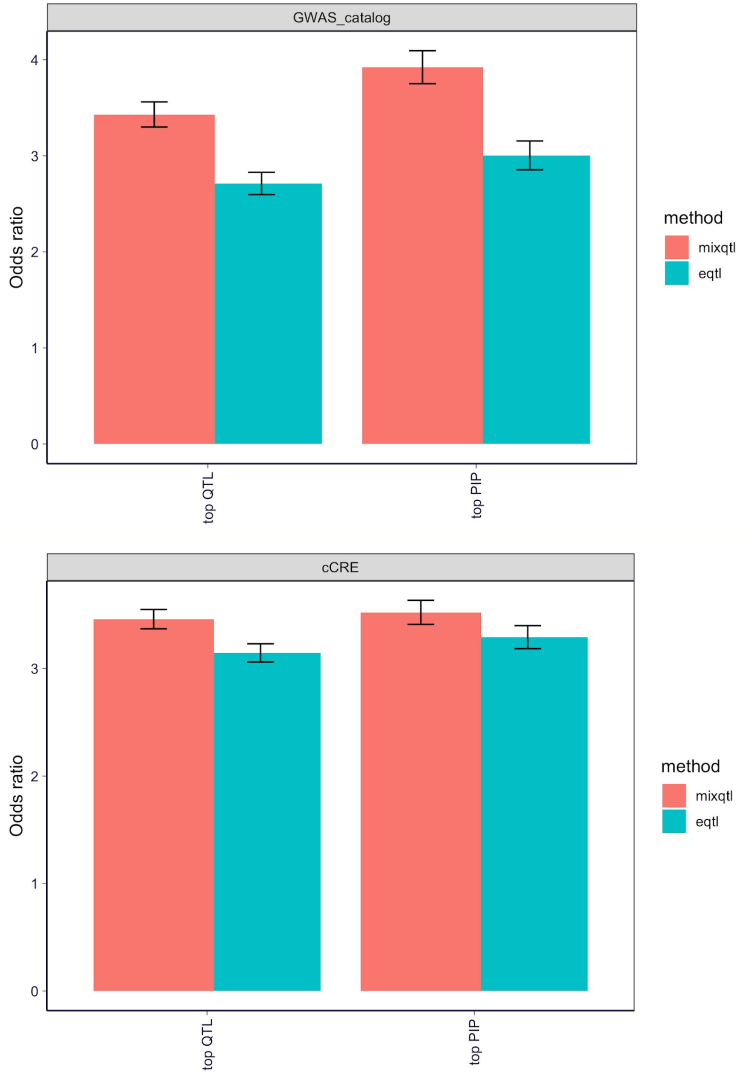
GWAS_catalog
Odds ratio
0
1
2
3
4
top QTL
top PIP
method
mixqtl
eqtl
cCRE
Odds ratio
0
1
2
3
top QTL
top PIP
method
mixqtl
eqtl

	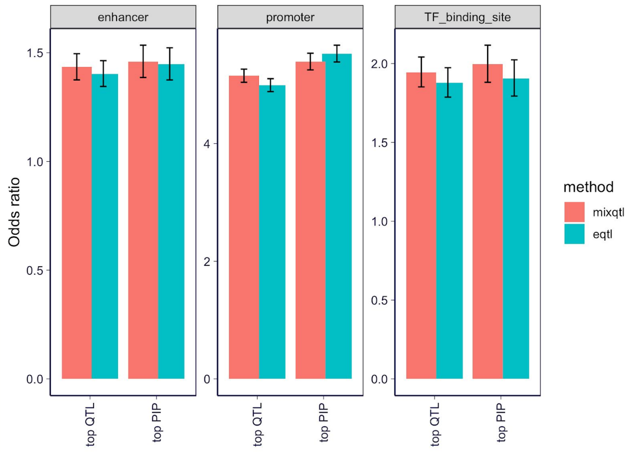 **Action:**<br><br>We added the analysis to the results and supplementary notes section 16.<br>We added the figures as the supplementary figure S16.
**3)** Gene expression prediction. How does mixQTL perform relative to PrediXcan? Also, I cannot really see any difference between "standard" and "mixPred" in Fig6B: the authors should support their claim with a better figure.	To improve visualization, we report now the median prediction performance in the supplementary table S2. We also changed figure 6B with violin plots showing the increased pearson correlation for different sample sizes.

sample_size	pairwise_diff	diff_ci95_low	diff_ci95_high	pval	median_mixpred	median_standard
67	0.05208	0.04687	0.0573	1.318e-72	0.1753	0.07011
75	0.04952	0.04386	0.05518	4.828e-58	0.1854	0.07935
84	0.04889	0.04357	0.05422	4.569e-63	0.198	0.1003
96	0.0473	0.04187	0.05274	1.35e-57	0.2141	0.1189
112	0.04332	0.0381	0.04854	4.884e-53	0.228	0.1519
134	0.0362	0.03104	0.04136	1.483e-39	0.2413	0.1953
168	0.02762	0.02278	0.03246	1.791e-27	0.2514	0.2192
224	0.01657	0.01198	0.02115	2.535e-12	0.2663	0.2536
335	0.006543	0.002176	0.01091	0.003354	0.2919	0.2868

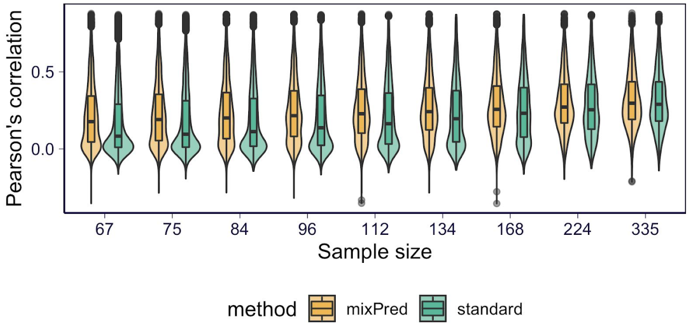

Action:
We updated Fig6B with the new violin plot and added the paired t test results as supplementary table S2.

4) Using the eQTLGen dataset as validation is a reasonable approach as it was derived from a huge sample size. However, there is quite a bit of heterogeneity in this dataset and I would be cautious when using it as a "ground truth": I would seriously consider replicating the results on several randomizations of the 100,000 variant-gene pairs to make sure that the results still hold.

We agree that eQTLGen, as “ground truth”, should be used with caution. We followed the recommendation and subsampled 10 sets of 100k variants. We observe that the substance of the results stayed the same across simulations. Specifically, we plotted the replication of Fig5C along with the PR and ROC curves in below.

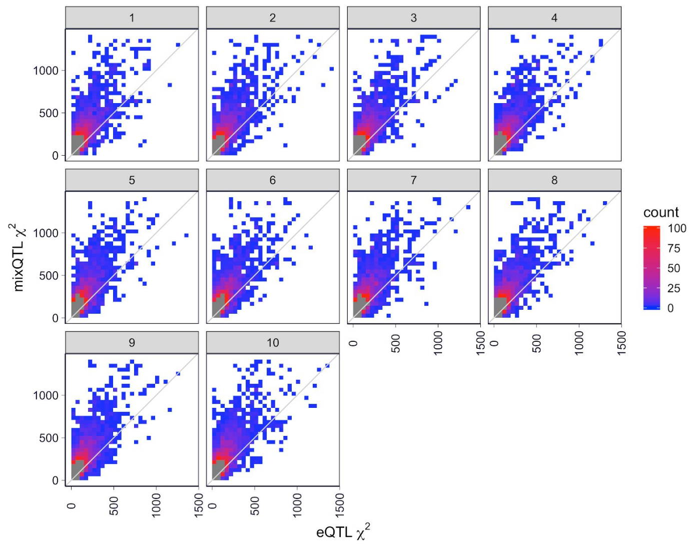
1
2
3
4
5
6
7
8
9
10
count
100
75
50
25
0
1000
500
0
0
500
1000
1500
mixQTL χ^2
eQTL χ^2

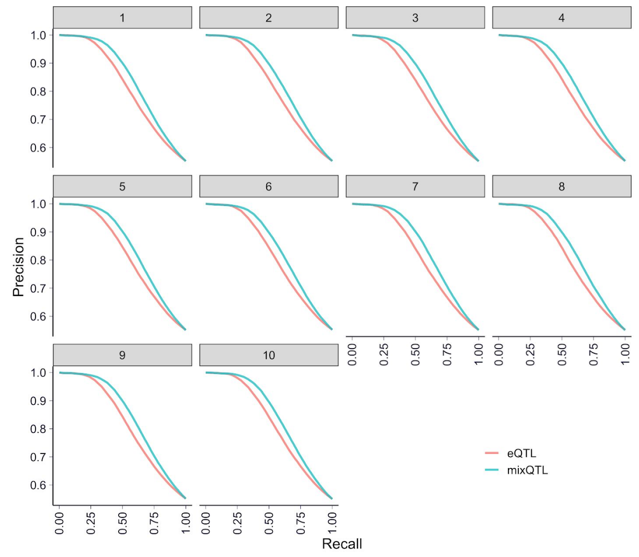
1
2
3
4
5
6
7
8
9
10
1.0
0.9
0.8
0.7
0.6
0.00
0.25
0.50
0.75
1.00
Precision
Recall
eQTL
mixQTL

<table>
<tr>
<td></td>
<td>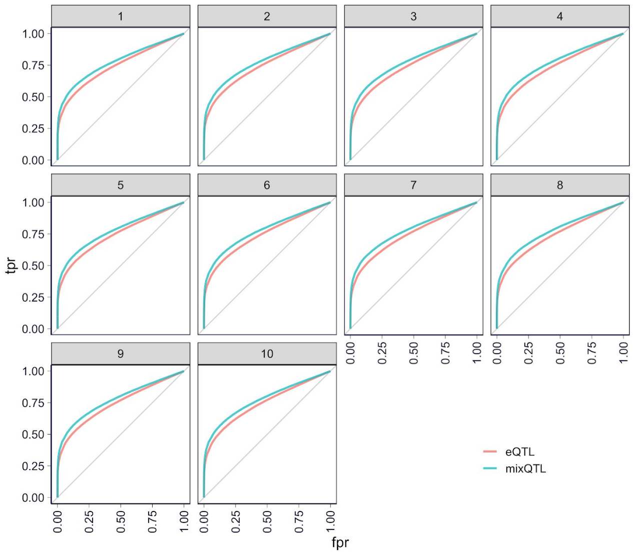

Action:
We added that we ran the analysis for multiple samples of eQTLGen results and found no substantive differences in the results to the results section.</td>
</tr>
<tr>
<td>5) What is the computational complexity of the overall algorithm? Is it linear with number of samples? Quadratic?</td>
<td>We ran mixQTL for all of the 49 tissues in GTEx (with 8 CPU cores). As shown in the figure, we plotted the sample size against the average runtime per gene (in seconds, clock time) and it is linear in sample size.</td>
</tr>
</table>

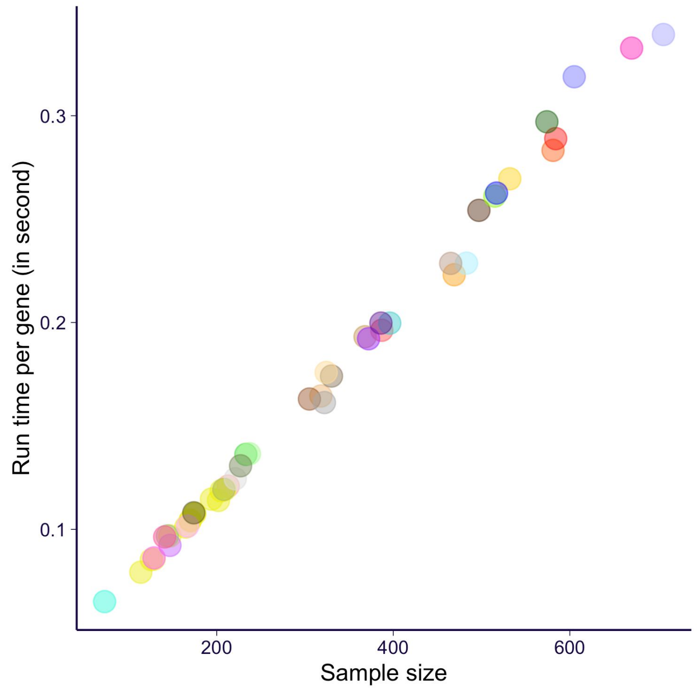

Action:
We added a sentence on this to the results section.
And we added the figure as supplementary figure S12.

Reviewer 3

Reviewer's comments	Response
The paper introduces a principled way to convert joint analysis of gene expression and ASE for eQTL calling, fine mapping, and prediction of genetically driven expression into a meta-analysis problem over independent linear regression. I like the work: I think modeling is elegant, and the addressed problems are relevant. That said, I think the work as it stands is not mature enough for publication.<br><br>Specifically, most analyses reported are simulations, and the current results from real data support a fraction of the claims and do not provide any new biological results/insights. Simulation results are important for debugging the code and exploring the model's behavior but are not helpful in testing the validity of the model assumptions, comparison to other methods. The authors claim improvements in eQTL calling, fine mapping and expression prediction. While each of these contributions could be sufficient for a separate paper, the manuscript fails to pin down the performance and practical value of each of these methods and provide the new biological insights with adequate experiments on real data. The paper should address the following practical questions for a reader.	We thank the reviewer for the helpful comments and suggestions. We address each point below.
**MAJOR COMMENTS**	
**1)** When should one use mixQTL on a dataset instead of standard TensorQTL, or WASP/RASQUAL for eQTL calling?	The best approach will depend on the computational capacity of the lab running the analysis. For small enough sample sizes, RASQUAL should be the method of choice. With current resources, the maximum sample size to run RASQUAL seems to be around 100. For larger samples, mixQTLs should be preferred over standard eQTL analysis,

	since it only adds a factor of 2 in computational cost. This is assuming that phased genotypes and allele specific expressions have been pre-computed.<br><br>**Action:**<br>We added these considerations to the discussion section.
How are the eQTLs found with mixQTL different from those found by the conventional approach quantitatively and functionally.	We examined the enrichment of top QTL/PIP in different functional annotations. We found that the top QTLs and top PIP variants from mixFine were more enriched among GWAS catalog variants and candidate cis-regulatory elements (https://www.nature.com/articles/s41586-020-2493-4) than the standard eQTL and fine-mapping methods. We did not find significant differences in enrichment differences for enhancer, promoter, and transcription factor binding sites.

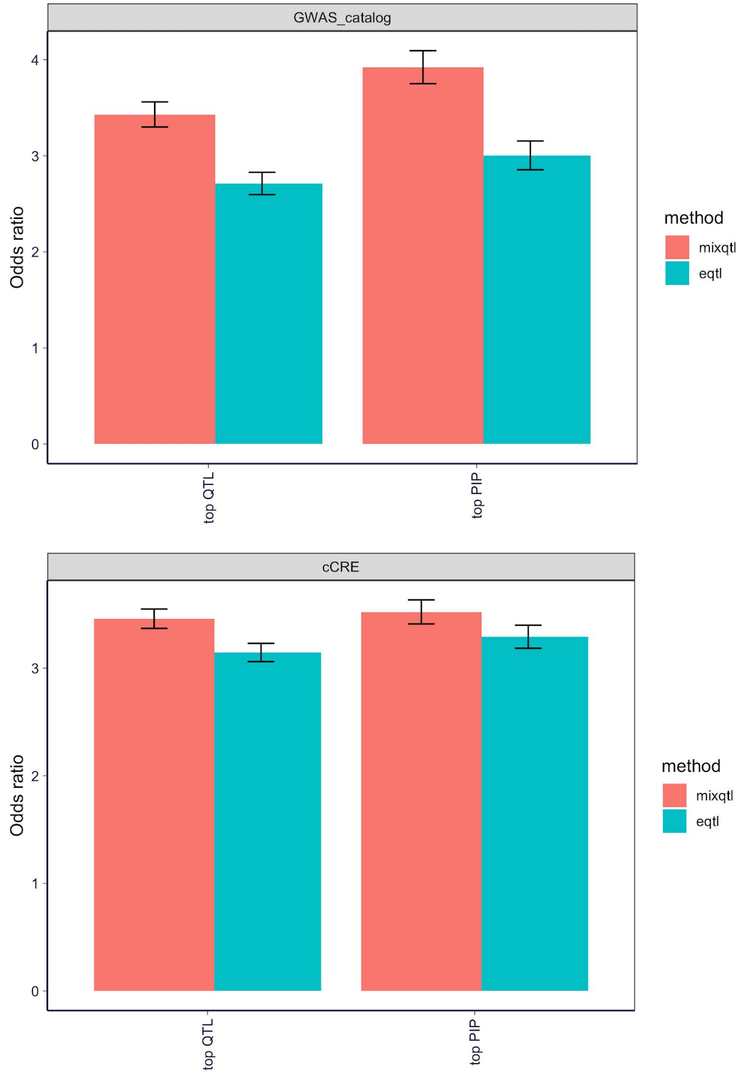
GWAS_catalog
Odds ratio
4
3
2
1
0
top QTL
top PIP
method
mixqtl
eqtl
cCRE
Odds ratio
3
2
1
0
top QTL
top PIP
method
mixqtl
eqtl

	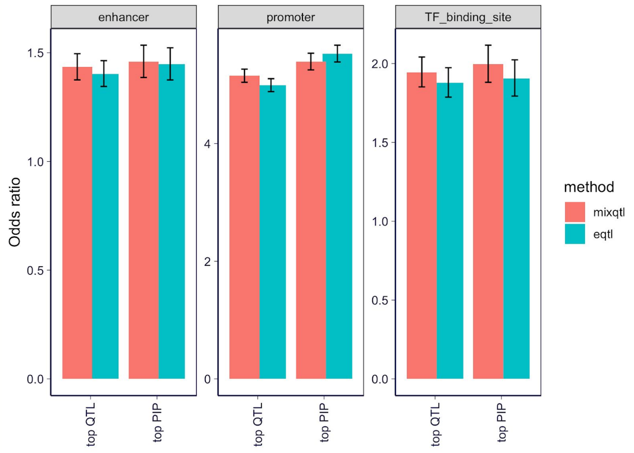<br>**Action:**<br><br>We added the analysis to the results and supplementary notes section 16.<br>We added the figure as supplementary figure S16.
The simulations are adequate for the speed comparisons, and the analysis of GTEx data in Figure 5 highlights the potential value of the method or the standard eQTL calling.<br>Is the value over WASP/RASQUAL here is only the speed?	The benefit of using mixQTL over WASP/RASQUAL is indeed about speed. Based on our test run and the runtime reported in the literature, it needs months to run RASQUAL on GTEx V8 whole blood (n = 670) even with multi-threading. And mixQTL takes less than 2 hrs to run on the same data. We found that in Kidney mixQTL was 723 times faster than RASQUAL and in Whole Blood it was 2480 times faster.
Now that the speed issue is resolved what do we find in GTEx v8 that we were unable to find before? Considering the motivation of the paper regarding the need for a faster method: "However, these methods are computationally too costly to be applied to sample sizes beyond a few hundred and as a result have not been applied to large-scale studies like GTEx, which includes over 17,000 samples across 49 tissues." it would be reasonable to expect the results to	We ran on all of the GTEx tissues with more than 70 samples. The full analysis included 15,201 samples covering 49 tissues and it took about 54 clock hours in total (used 8 CPU cores and 16gb RAM).<br><br>Our approach allowed us to identify more genes (1440 on average across tissues) and more eQTLs (618k on average across tissues), which are also more enriched in functional annotations such as cCRE

include the results from the complete GTEx data and the new biological insights found by this new analysis. Providing the results from the complete GTEx data would also be also a great resource for the community.	and GWAS catalog. For example, this should lead to improved interpretation of GWAS loci. Furthermore, we make our mixQTL summary statistics publicly available as an improved resource (the links are listed in supplementary table S1).
**2)** Should one use mixFine instead of PLASMA or the Zou et al. 2019 methods, which are both very similar fine-mapping techniques designed to utilize ASE and the aFC model.	Zou et al and Wang et al combine the eQTL and ASE z-scores even though the eQTL and ASE results are obtained from completely different tests and definitions of expression level (read count or inverse normalized count). In contrast, mixFine is derived directly from the likelihood of the data so it provides theoretical justification of the approach. We expect that, in principle, mixFine should produce results similar to Zou et al and Wang et al.<br><br>However, we were not able to perform the comparison since both software implementations had bugs or other errors not easily solvable. We opened issues in their respective github repositories but none authors of the methods were responsive to our request for help.<br><br>For Wang et al method, we found that the code has some missing components. At least one other user had the same problem we encountered and opened an issue in the GitHub repository. We followed up on the same issue but could not resolve the issue yet. The issue is at https://github.com/austintwang/plasma/issues/3#issuecomment-661161283.<br><br>For Zou et al methods, we tried to pack their pipeline up into an R package https://github.com/liangyy/finemapAim but during testing their code, we found an unexpected behavior of their code, the output changes when we change the ID of one sample in the input. We opened an issue in their GitHub repository https://github.com/jzou1115/aim/issues/1) but have not got a reply yet.

3) Should one use mixPred instead of the standard Susie/elastic net for predicting genetically driven gene expression? This analysis is presented in figure 6B. But, I cannot understand how Figure 6B shows an improvement? Please clarify if this is the case with appropriate visualization/analysis.

Yes, we should use mixFine instead of the standard elastic net. We will implement this in the future.

To improve visualization, we report now the median prediction performance in the supplementary table S2. We also changed Figure 6B with violin plots showing the increased pearson correlation for different sample sizes.

sample_size	pairwise_diff	diff_ci95_low	diff_ci95_high	pval	median_mixpred	median_standard
67	0.05208	0.04687	0.0573	1.318e-72	0.1753	0.07011
75	0.04952	0.04386	0.05518	4.828e-58	0.1854	0.07935
84	0.04889	0.04357	0.05422	4.569e-63	0.198	0.1003
96	0.0473	0.04187	0.05274	1.35e-57	0.2141	0.1189
112	0.04332	0.0381	0.04854	4.884e-53	0.228	0.1519
134	0.0362	0.03104	0.04136	1.483e-39	0.2413	0.1953
168	0.02762	0.02278	0.03246	1.791e-27	0.2514	0.2192
224	0.01657	0.01198	0.02115	2.535e-12	0.2663	0.2536
335	0.006543	0.002176	0.01091	0.003354	0.2919	0.2868

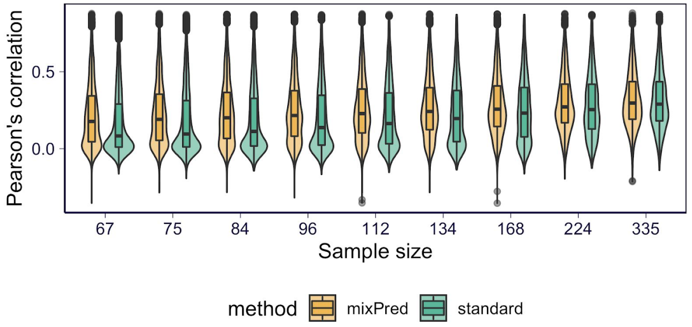

I realize that the above questions have been partially explored via simulation. Still, I believe a real data comparison using orthogonal

sources of evidence such as functional enrichment, reproducibility, etc. will be the appropriate way to evaluate the methods.	
**MINOR COMMENTS**	
**1)** The Abstract and Introduction sections go back and forth between the fine mapping and eQTL calling, starting from the 2nd and the 3rd sentence in the abstract. Please streamline the narrative to improve readability.	We updated the abstract to address this issue.
**2)** The justification for error definitions on line 65 is not clear; the same goes for e^asc in line 67. Are the shared variance term and the variance scaling by mean appropriate assumptions that fit the real data? Does this assume that the biological variance to be similar in allelic imbalance and gene expression?	Note that the linear relationship between the mean and the variance is the standard assumption made for RNA-seq data. RASQUAL and WASP do so by assuming a negative binomial model for the counts. In our case, we achieve the linearity by dividing the sigma^2 by the count. The detailed explanation is shown in supplementary notes 7.2. The e^trc and e^asc is part of the technical noise.<br><br>**Action:**<br>We added the following paragraph after line 65 (now it is line 72-73)<br><br>Here we let the ϵ terms have variance inversely proportional to the actual count and by doing so, we ensure that the variance of the count scales approximately linearly to the mean of the count (Supplementary Notes 7.2).
**3)** I find the discussion regarding the "technical" and "biological noise" terms rather confusing (text between lines 66 and 67). Is it possible to plot these terms against each other in an example dataset by estimating the over-dispersion and count noise?	We realize now that the description is a bit confusing. We rewrote the paragraph and simplified the description.<br><br>Now the paragraph reads<br><br>We further simplified the models by combining the two allele-specific counts and defining the baseline abundance variation as a random

	effect z_i ($\log \theta_{0, i}$ = population mean + z_i). Then, we merge the total count term $\epsilon_i^\trc$ and z_i into one term $\widetilde{z}_i$ since $\epsilon_i^\asc$ is approximately independent to both of them. See Methods section and Supplementary Note 10.1) ...
**4)** In all relevant figures, please clarify the error bars (SE, 95% CI, std, Quantiles, ...).	We added the clarifications accordingly.
**5)** It is not clear in the text if mixQTL finds multiple independent eQTLs per gene or just the top one. Or if it needs to be used together with mixFine to find those?	mixQTL simply lists the statistical association between expression and genotype. In other words, mixQTL reports the summary statistics of all variant/gene pairs. The fine-mapping methods are able to find allelic heterogeneity, namely identifying one or more causal signals for a region.
**6)** In the fine-mapping section, what fraction are "consensus snps"? Are the fine map SNPs from mixFine functionally similar/different to/from those from Susie? What about those high confidence SNPs from Susie that do not show up in mixFine?	The fine-mapped SNPs from mixFine are functionally similar to those from the standard approach. The two methods are both intended to capture the causal variants of the cis-regulation. With allele-specific read counts being integrated, mixFine may identify signals that could be missed out by the standard approach due to the limited power. But, due to the random noise, it is possible that the signal identified in the standard approach gets missed out by mixFine.
**7)** There are invalid characters substituted for $\leq$ in supplementary materials, see the paragraph before section 14.1.	We fixed these typos, thanks!

Reviewer #1 (Remarks to the Author):

I thank the authors for the improvements and changes made to both their method and manuscript. All my concerns were addressed in the revision, and I have no further concerns.

Reviewer #2 (Remarks to the Author):

The authors successfully addressed three of my concerns regarding their study (1, 4, 5). I however have questions regarding two of the previous comments I made (2 and 3):

R2: Fine-mapping. I'd be curious to see where the causal variants spotted by each respective approach do locate in terms of functional annotations (e.g. Encode).

A: Following the reviewer's suggestion, we ran mixFine and standard eQTL approach in 26 tissues with small sample sizes (n<260) where we thought that the gains would be most apparent. And we examined the enrichment of top QTL/PIP in different functional annotations. We found that the top QTLs and top PIP variants from mixQTL and mixFine were more enriched among GWAS catalog variants and candidate cis-regulatory elements (https://www.nature.com/articles/s41586-020-2493-4) than the standard eQTL and fine-mapping methods. We did not find significant differences in enrichment differences for enhancer, promoter, and transcription factor binding sites.

R: I am a bit puzzle by these results. You do see a clear enrichment for cis regulatory elements (CREs) but not at all for enhancers and promoters. Since enhancers/promoters are the two most common types of CREs in the genome into which eQTLs are found to be enriched, how can these contradictory results can be reconciled? Some explanation seems required here.

R3: Gene expression prediction. How does mixQTL perform relative to PrediXcan? Also, I cannot really see any difference between "standard" and "mixPred" in Fig6B: the authors should support their claim with a better figure.

A: To improve visualization, we report now the median prediction performance in the supplementary table S2. We also changed figure 6B with violin plots showing the increased pearson correlation for different sample sizes.

R: You successfully edited Fig6B. However, why did you not try to compare mixpred with another widely used methods such as prediXscan? Comparing only methods you developed seems to me not convincing enough. Readers will certainly wonder how your predictions do compare to those offered by others widely used methods.

Reviewer #3 (Remarks to the Author):

The authors have adressed all my comments.

Reviewer 2

Reviewer's comments	Response
The authors successfully addressed three of my concerns regarding their study (1, 4, 5). I however have questions regarding two of the previous comments I made (2 and 3):	We thank the reviewer for the follow-up comments.
R2: Fine-mapping. I'd be curious to see where the causal variants spotted by each respective approach do locate in terms of functional annotations (e.g. Encode).<br><br>A: Following the reviewer's suggestion, we ran mixFine and standard eQTL approach in 26 tissues with small sample sizes (n<260) where we thought that the gains would be most apparent. And we examined the enrichment of top QTL/PIP in different functional annotations. We found that the top QTLs and top PIP variants from mixQTL and mixFine were more enriched among GWAS catalog variants and candidate cis-regulatory elements (https://www.nature.com/articles/s41586-020-2493-4) than the standard eQTL and fine-mapping methods. We did not find significant differences in enrichment differences for enhancer, promoter, and transcription factor binding sites.<br><br>**R: I am a bit puzzle by these results. You do see a clear enrichment for cis regulatory elements (CREs) but not at all for enhancers and promoters. Since enhancers/promoters are the two most common types of CREs in the genome into which eQTLs are found to be enriched, how can these contradictory**	We have reworded the paragraph in the results section to clarify that we do see enrichment of enhancers and promoters and that what is not significant is the difference in enrichment between mixQTL and eQTLs. Another point to clarify is that the enrichment of enhancers, promoters and TF binding sites are less extreme than we find with the cCRE probably due to the cross-tissue annotation used for these compared to tissue-specific ones used for cCRE.<br><br>We revised the section as follows:<br>"We found that the variants with the most significant mixQTL p-value or the highest mixFine PIP were more enriched in GWAS catalog variants and cCREs than the standard approach. We found enrichment of enhancer, promoter, and transcription factor binding sites but the difference in enrichment between mixQTL and standard QTL methods was not significant (Supplementary Figure S16). The reduced enrichment compared to cCREs are likely due to the fact that we used tissue-specific annotations for cCREs and cross-tissue annotations for enhancers, promoters, and TF."

**results can be reconciled? Some explanation seems required here.**	
R3: Gene expression prediction. How does mixQTL perform relative to PrediXcan? Also, I cannot really see any difference between "standard" and "mixPred" in Fig6B: the authors should support their claim with a better figure.<br><br>A: To improve visualization, we report now the median prediction performance in the supplementary table S2. We also changed figure 6B with violin plots showing the increased pearson correlation for different sample sizes.<br><br>**R: You successfully edited Fig6B. However, why did you not try to compare mixed with another widely used methods such as prediXscan? Comparing only methods you developed seems to me not convincing enough. Readers will certainly wonder how your predictions do compare to those offered by others widely used methods.**	The main prediction approach in PrediXcan is elastic net, which is the model we used to benchmark mixPred.<br><br>We clarify in the revised version (On page 16, in the caption of Figure 6 caption and in section 11) that we are comparing to the standard elastic net method as implemented in PrediXcan.
